# Interpretable self-driving sputtering epitaxy reveals human-usable growth rules for *β*-Ga_2_O_3_ films

**DOI:** 10.1038/s41467-026-76533-0

**Published:** 2026-08-13

**Authors:** Yuki K. Wakabayashi, Yui Ogawa, Franz Benedict Romero, Coleman Wagner, Takuma Otsuka, Yoshitaka Taniyasu

**Affiliations:** 1 Basic Research Laboratories, NTT, Inc., Atsugi, Kanagawa Japan; 2 Communication Science Laboratories, NTT, Inc., Soraku-gun, Kyoto, Japan

**Keywords:** Electronic devices, Computational methods

## Abstract

Self-driving laboratories are powerful tools for navigating high-dimensional process spaces, yet Bayesian-optimization decision layers optimize black-box objective functions without distilling transferable process understanding. Here, we demonstrate a mathematically interpretable self-driving laboratory framework that transforms autonomous optimization data into human-executable growth rules. As a benchmark, we apply this framework to sputtering, addressing a challenge in sputtering epitaxy: realizing high-quality *β*-Ga_2_O_3_ heteroepitaxy and single-crystalline *β*-Ga_2_O_3_ homoepitaxy. By combining Bayesian optimization with automated optical evaluation of the Urbach energy as a sub-bandgap disorder metric, the self-driving system identifies heteroepitaxial conditions yielding an Urbach energy of 182 meV, below previously reported values for sputtered *β*-Ga_2_O_3_ films. Importantly, the optimized growth window is transferable across substrates, realizing single-crystalline *β*-Ga_2_O_3_ homoepitaxy, corroborated by scanning transmission electron microscopy. To convert closed-loop data into interpretable growth rules, we train a random forest surrogate and reveal that the growth landscape is described by additive contributions from four growth parameters, with a temperature–oxygen interaction. This structure suggests a human-executable strategy with sequential one-dimensional tuning followed by focused two-dimensional refinement, validated by human-executed re-optimization, yielding a reduced Urbach energy of 163 meV. This establishes an interpretable self-driving workflow that converts autonomous optimization data into validated, human-usable growth rules.

## Abstract (web summary)

Materials growth requires navigating complex conditions. Here, the authors combine automated sputtering, Bayesian optimization, and interpretable machine learning in a self-driving laboratory to optimize *β*-Ga2O3 growth and extract practical rules.

## Introduction

Self-driving laboratories (SDLs) are rapidly transforming materials research by closing the loop between automated experimentation^1,2^ and machine-learning (ML) decision making^3–5^; with Bayesian optimization (BO)^6–11^ emerging as a particularly efficient engine for navigating high-dimensional process spaces^12–17^. Yet, despite their success at identifying optimal recipes with fewer trials, many closed-loop optimization workflows still treat the process–property relationship as a black-box objective function^18–20^: they output optimal conditions but do not reveal which process parameters govern performance, how each knob reshapes the response, or whether the system is controlled by simple additive trends versus complex parameter interactions. This lack of distilled process knowledge can hinder reproducibility, limit the reuse of growth rules across different tools, substrates, and material systems, and slow the conversion of autonomous optimization data into robust, actionable process knowledge. Addressing this gap requires self-driving frameworks that go beyond recipe discovery to expose dominant controls and parameter interactions in a human-usable form. Such interpretable SDLs are essential to bridge autonomous optimization and transferable process engineering.

A stringent benchmark for an interpretable SDL is the sputter epitaxy of *β*-Ga_2_O_3_ films, a promising ultra-wide-bandgap semiconductor for next-generation power electronics and solar-blind deep-ultraviolet photodetectors, where device performance is highly sensitive to crystalline quality and optical disorder^21–27^. High-quality epitaxial *β*-Ga_2_O_3_ has been achieved by established epitaxy techniques such as chemical vapor deposition (CVD)^28–30^ and molecular beam epitaxy (MBE)^31–34^; however, achieving comparably high-quality epitaxy by sputtering, which is an industry-standard, low-cost route for large-area deposition, remains elusive^35–37^. The energetic growth environment and limited adatom mobility, together with narrow phase-stability margins in complex oxides, make epitaxial control challenging. As a result, sputtered Ga_2_O_3_ films are often amorphous, polycrystalline, or mixed-phase (e.g., *ε*- and *κ*-Ga_2_O_3_)^35,38–40^. These characteristics make sputtered *β*-Ga_2_O_3_ an ideal and stringent testbed for evaluating whether SDLs can go beyond black-box recipe optimization: the challenge is not only to achieve high-quality epitaxy by sputtering, but also to extract interpretable, human-executable growth rules from closed-loop data.

Here, we address this challenge by realizing an interpretable SDL for the sputter epitaxy of *β*-Ga_2_O_3_ films. We construct a self-driving sputtering platform that couples automated RF magnetron sputtering with automated optical analysis and BO of the Urbach energy (*E*_U_)^41,42^, a metric of band-tail states and sub-bandgap optical disorder. Using this framework, we rapidly identify a high-quality heteroepitaxial growth window for *β*-Ga_2_O_3_ on C-plane Al_2_O_3_ (0001), achieving a minimum *E*_U_ of 182 meV by the 56th run (66 total closed-loop runs). The resulting film exhibits high crystalline quality, evidenced by a narrow *β*-Ga_2_O_3_ ($\bar{2}01$) rocking-curve full width at half maximum (FWHM) of 1.78°, as well as high optical quality, reflected in the low Urbach energy, together with a smooth surface with 1.15–1.27 nm roughness. Importantly, applying the BO-identified growth window to *β*-Ga_2_O_3_ substrates enabled single-crystalline homoepitaxy without additional optimization. Controlled homoepitaxial tests under low-*E*_U_ conditions and under a high-*E*_U_ condition outside the identified low-*E*_U_ window further support a correspondence between the heteroepitaxial *E*_U_ landscape and homoepitaxial crystalline quality within the tested process space. This cross-substrate correspondence suggests that the self-driving process identified growth-relevant trends not limited to a substrate-specific recipe. Beyond recipe discovery, we introduce an explainable machine-learning analysis workflow that interrogates a tree-based surrogate model to distill closed-loop data into human-usable growth rules. This workflow reveals a predominantly additive growth landscape with a residual temperature–oxygen coupling that defines the narrow window for high-quality growth. This structure suggests a human-executable optimization strategy: sequential one-dimensional tuning followed by a final two-dimensional refinement in the (*T*, O_2_) plane. We further validate this rule by human-executed re-optimization, which reaches *E*_U_ = 163 meV, lower than the original BO optimum. Moreover, expanding the dataset to 215 growth runs through broader sampling of the growth-parameter space improves surrogate predictive fidelity while preserving a predominantly additive structure with a temperature–oxygen interaction, supporting the robustness of the distilled rule. Together, these results establish an interpretable SDL framework that transforms autonomous experimentation into transferable process understanding beyond optimization of a black-box function.

## Results

### Self-driving sputter growth with automated optical analysis

**Fig. 1:**
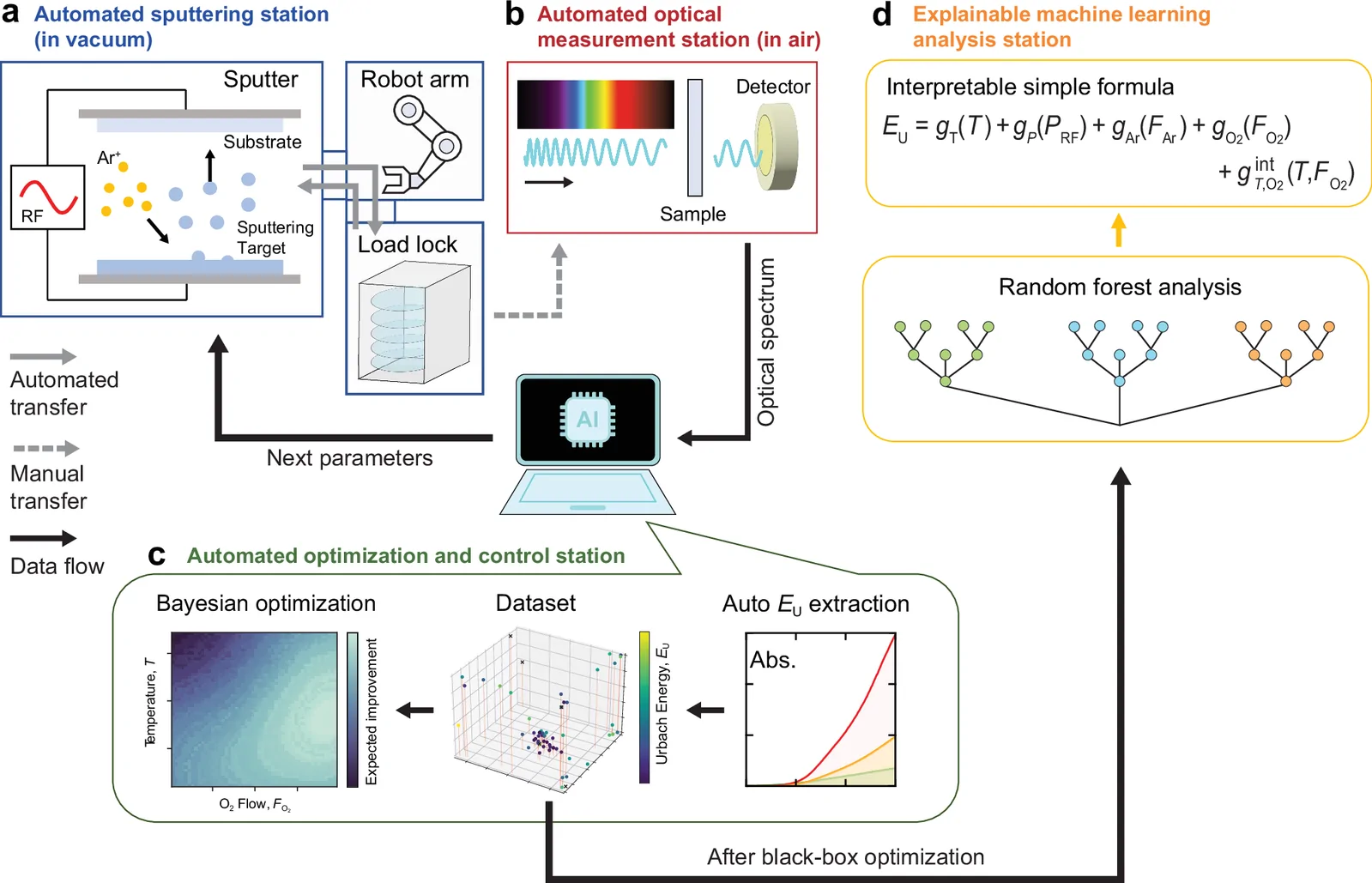
Closed-loop self-driving sputter epitaxy with explainable machine learning. **a** Automated RF sputtering station operated in vacuum for thin-film growth. **b** Automated optical measurement station operated in air to acquire optical transmittance spectra. **c** Automated optimization and control station that performs (1) auto-extraction of the Urbach energy *E*_U_ from the optical spectra, (2) dataset update, and (3) Bayesian optimization to propose the next growth conditions and automatically triggers the subsequent sputter run. **d** Explainable ML analysis station, where a random forest model is trained on the accumulated dataset and distilled into human-interpretable relations for process understanding.

We establish an interpretable self-driving sputtering workflow in which a physically meaningful disorder metric guides closed-loop exploration of a high-dimensional growth-parameter space. Figure 1 summarizes the workflow in this study. *β*-Ga_2_O_3_ films with a target nominal thickness of 55 nm were grown on a 2-inch C-plane Al_2_O_3_ wafer by RF magnetron sputtering while varying four growth parameters: substrate temperature *T*, RF power ${P}_{{{{\rm{RF}}}}}$, and Ar and O₂ flow rates (${F}_{{{{\rm{A}}}}{{{\rm{r}}}}}$, ${F}_{{{{{\rm{O}}}}}_{2}}$) (Fig. 1a) (see Methods section “Self-driving sputtering system” and Supplementary Fig. 1). After each deposition, the transmittance spectrum of the film was measured (Fig. 1b) and automatically analyzed to extract the Urbach energy *E*_U_ (Fig. 1c) (see Methods section “Automated optical analysis”), which serves as a sensitive figure of merit for band-tail states and optical disorder in ultra-wide-bandgap semiconductors^41,43^. The extracted value was fed back to a Gaussian-process (GP)-based BO algorithm tailored to thin-film growth under occasional experimental failures (see Methods section “Bayesian optimization with failures and adaptive prior mean”)^44,45^, which proposed the next set of growth parameters and automatically initiated the subsequent growth run on the next wafer in the stock, thereby forming a semi-autonomous closed loop. A pseudocode-style summary of the closed-loop BO procedure used in the self-driving sputtering system is provided in Supplementary Box 1. In the present implementation, each loop consists of (i) manual loading of up to five 2-inch wafers into the load-lock (wafer stock), (ii) fully automated, vacuum-integrated transfer between the load-lock and the sputter growth chamber by a built-in robot arm, sputter growth according to the BO-suggested parameters, and automatic return to the load-lock, (iii) manual transfer of the grown film from the load-lock to the optical setup, and (iv) fully automated optical measurement and spectral analysis, followed by BO model update and next-point selection that automatically triggers the next sputter growth. Because wafer handling inside the sputter system is already robotized, the remaining manual transfer step in (iii) could also be replaced by robotic handlers, so that the current semi-autonomous workflow can be straightforwardly extended to a fully autonomous self-driving sputter growth platform. Finally, an interpretable machine-learning analysis was applied to the BO dataset to extract a simple, human-readable rule for *E*_U_ (Fig. 1d).

**Fig. 2:**
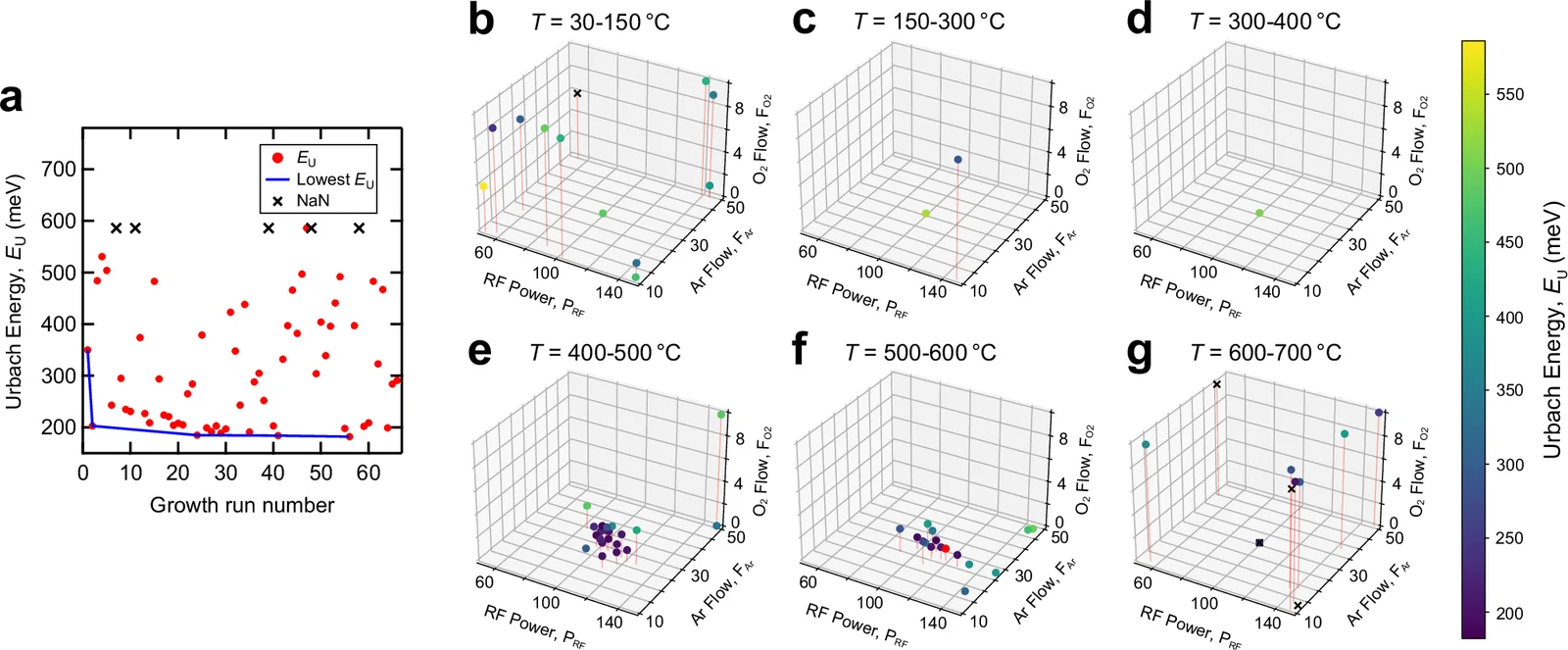
Bayesian optimization of *E*_U_ over a four-dimensional growth-parameter space. **a** The experimental *E*_U_ (red circles) as a function of growth run number. The blue line indicates the lowest *E*_U_ achieved up to each run. Black crosses indicate runs for which the *E*_U_ could not be extracted (NaN). The experimental *E*_U_ in the three-dimensional parameter space ${P}_{{{{\rm{RF}}}}}-{F}_{{{{\rm{Ar}}}}}-{F}_{{{{{\rm{O}}}}}_{2}}$ for different *T* windows of **b** 30–150 °C, **c** 150–300 °C, **d** 300–400 °C, **e** 400–500 °C, **f** 500–600 °C, and **g** 600–700 °C. Marker color represents *E*_U_ (color bar, meV), visualizing how Bayesian optimization concentrates sampling. In (**f**), the red circle represents the lowest *E*_U_ (182 meV). In (**b**–**g**), black crosses indicate runs for which the *E*_U_ could not be extracted (NaN).

Across successive self-driving runs, the system explores and refines growth conditions, resulting in a steady reduction in *E*_U_. Figure 2a shows the evolution of the *E*_U_ during the 66 runs of the self-driving sputter experiments. The first seven runs used a pragmatic baseline set of parameters (fixed ${P}_{{{{\rm{RF}}}}}$, ${F}_{{{{\rm{Ar}}}}}$ and ${F}_{{{{{\rm{O}}}}}_{2}}$) and swept the substrate temperature over 30–700 °C to confirm stable operation of the newly constructed sputtering system over a broad temperature range and to seed BO. Starting from these initial data, the BO algorithm proposed the growth parameters for the subsequent 59 runs at each iteration. As the growth run number increases, the grown films have a broad range of *E*_U_ values, reflecting exploratory proposals by BO, whereas the lowest *E*_U_ values at that time decrease and eventually reach 182 meV at (*T*, ${P}_{{{{\rm{RF}}}}}$, ${F}_{{{{\rm{Ar}}}}}$, ${F}_{{{{{\rm{O}}}}}_{2}}$) = (507 °C, 119 W, 26 cm^3^ STP min^-1^, 1 cm^3^ STP min^-1^). This is the lowest *E*_U_ reported for sputtered *β*-Ga_2_O_3_ films, improving on the previous best value of 280 meV^47^, and it is also lower than a representative metal-organic chemical vapor deposition (MOCVD)-grown report (220 meV)^46^ (Table 1)^31,46–65^, highlighting that self-driving optimization can push sputtered *β*-Ga_2_O_3_ into an improved optical-quality regime. A replication growth performed at the identified optimum conditions yielded an *E*_U_ of 184 meV, consistent with the best run (182 meV), and additional replicate growths at representative conditions confirmed that the observed *E*_U_ trends are reproducible beyond run-to-run scatter (Supplementary Note 4).

**Table 1:** Benchmark of optical, structural, and surface quality for *β*-Ga_2_O_3_ films on C-plane Al_2_O_3_ grown by representative growth methods

Method	*E*_U_ (meV)	($\bar{2}01$) RC-FWHM (°)	AFM RMS (nm)	Ref.
RF sputter (this work)	182	1.78	1.15–1.27	This work
RF sputter (literature)	280	N/A	N/A	^47^
Low-pressure CVD	150	1.18–1.50	1.82–3.50	^48–51^
MOCVD	220–345	0.60–2.25	1.28–1.69	^46,52–58^
PAMBE	150	1.0	0.21–7	^59–62^
MBE	320	0.9–1.9	0.88	^31,63,64^
halide-VPE	N/A	0.33	N/A	^65^

*E*_U_, *β*-Ga_2_O_3_ ($\bar{2}01$) rocking-curve FWHM, and RMS roughness are compared between this work (RF sputtering) and representative literature reports. Ranges indicate reported values across references; N/A denotes not reported. A more comprehensive list and details are provided in Supplementary Table 3.

Mapping the measured *E*_U_ across the multidimensional growth-parameter space reveals structured low-disorder regions interspersed with exploratory sampling. Figure 2b-g map experimental *E*_U_ onto the ${P}_{{{{\rm{RF}}}}}-{F}_{{{{\rm{Ar}}}}}-{F}_{{{{{\rm{O}}}}}_{2}}$ space across different temperature ranges. We observe dense clusters of low-*E*_U_ films around sweet spots in the 400–500 °C and 500–600 °C regions, while sparsely distributed high-*E*_U_ points in all temperature regions appear as BO explores in the four-dimensional growth space. This feature clearly reflects the characteristic balance between exploiting known favorable conditions and exploring new ones in the BO algorithm. The quantitative dependence of *E*_U_ on the growth parameters in this high-dimensional space, as well as correlations between the parameters, can be distilled into an intuitive, human-interpretable picture by post hoc explainable ML analysis of the same dataset, as described later.

### Crystallographic and optical properties of optimized films

**Fig. 3:**
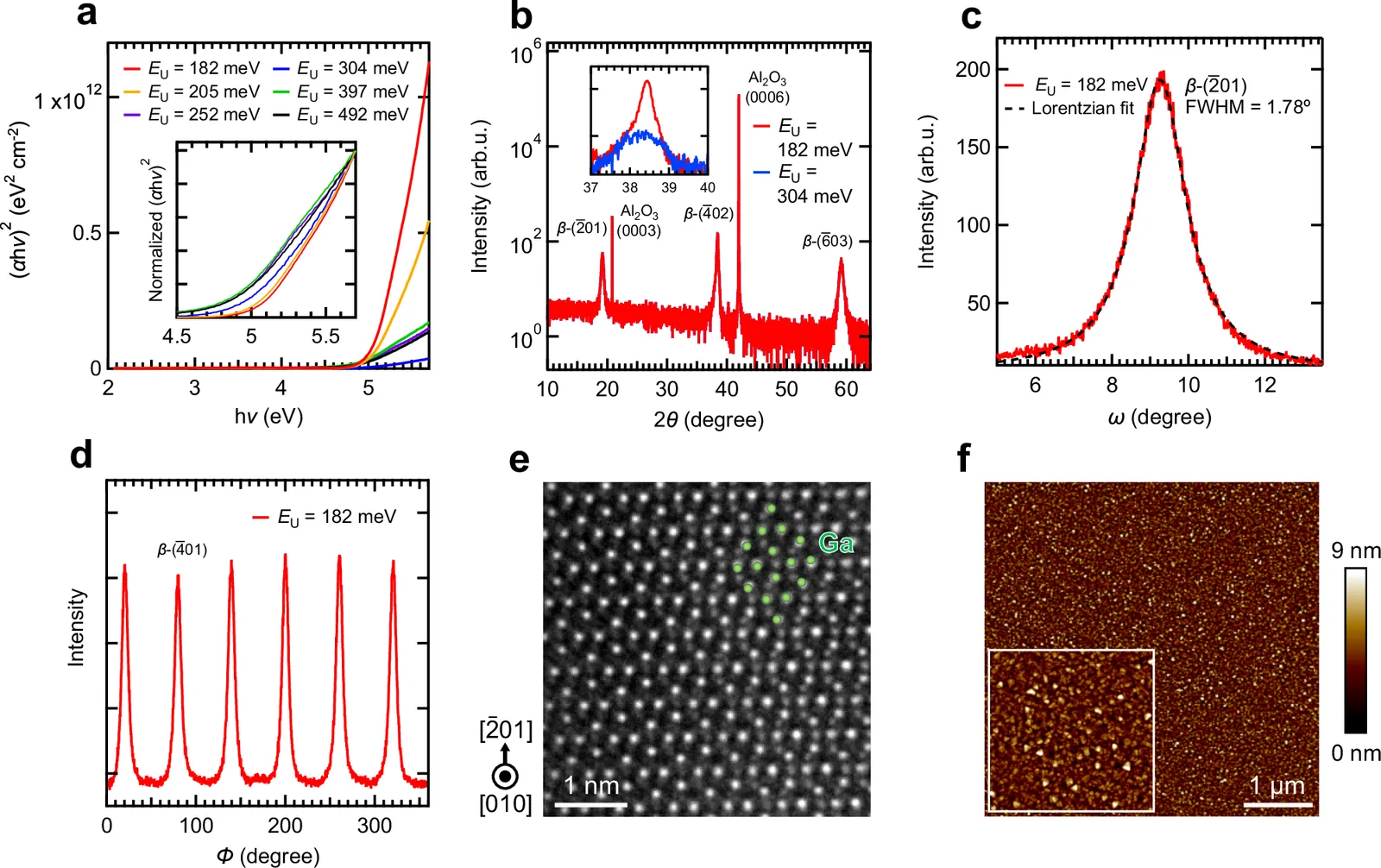
Optical and structural properties of the optimized heteroepitaxial *β*-Ga_2_O_3_ films on C-plane Al_2_O_3_. **a** Tauc plots ${\left(\alpha {{{\rm{h}}}}\nu \right)}^{2}$ derived from optical transmittance spectra for representative films with different *E*_U_ values. The inset in **a** shows normalized ${\left(\alpha {{{\rm{h}}}}\nu \right)}^{2}$ near the absorption edge. **b** XRD $\theta -2\theta$ scan for the optimized film (*E*_U_ = 182 meV). The inset in (**b**) also shows the scan for the film with *E*_U_ = 304 meV. **c** Rocking curve (*ω*-scan) of the *β*-Ga_2_O_3_ ($\bar{2}01$) reflection for the optimized film, with a Lorentzian fit (dashed line). **d** In-plane *ϕ*-scan of the *β*-($\bar{4}01$) reflection for the optimized film. **e** Atomic-resolution HAADF-STEM image of the optimized film viewed along the [010] direction of the *β*-Ga_2_O_3_ (parallel to the [$1\bar{1}00$] direction of th**e** C-plane Al_2_O_3_ substrate). In (**e**), green spheres indicate Ga-occupied columns. Similar HAADF-STEM features were observed at three spatially separated locations of the same film. **f** AFM image (5 µm × 5 µm) of the optimized film. The inset in **f** shows a magnified view (1 µm × 1 µm). Similar AFM surface morphologies were observed at three spatially separated locations of the same film. Source data are provided as a Source Data file.

Optimizing *E*_U_ is accompanied by a systematic sharpening of the absorption edge and suppression of sub-bandgap absorption in sputtered *β*-Ga_2_O_3_ films. Figure 3a shows the optical absorption behavior of *β*-Ga_2_O_3_ films on a 2-inch C-plane Al_2_O_3_ wafer spanning a wide range of *E*_U_. The optimized film (*E*_U_ = 182 meV) exhibits a steep absorption edge with a band gap energy (*E*_g_) of 5.04 eV, and a strong above-bandgap absorption. Notably, the absorption coefficient at 5.5 eV reaches *α* = 1.5 × 10^5 ^cm^−^^1^, which is higher than a representative value reported for MOCVD-grown *β*-Ga_2_O_3_ films (*α* ≈ 8.4 × 10^4 ^cm^−1^ at 5.5 eV)^66^, highlighting the high optical quality of the optimized film. In contrast, films with larger *E*_U_ show a pronounced sub-bandgap (in-gap) absorption tail. When the spectra are normalized (inset), increasing *E*_U_ systematically enhances the in-gap absorption and shifts the nominal *E*_g_ to lower photon energies (e.g., 4.86 eV for *E*_U_ = 492 meV). These results confirm that using *E*_U_ as the objective in BO directly leads to a substantial improvement in optical quality.

Consistent with the optical improvement at lower *E*_U_, the *E*_U_-optimized films also exhibit enhanced crystallographic quality. The crystalline quality of the BO-optimized *β*-Ga_2_O_3_ film was examined by X-ray diffraction (XRD) and compared with that of a non-optimized film (*E*_U_ = 304 meV) (Fig. 3b). For the optimized *β*-Ga_2_O_3_ film, intense and well-defined peaks from the ($\bar{2}01$)-oriented *β*-Ga_2_O_3_ phase are observed^60^, while no *κ*-Ga_2_O_3_ or other secondary phases are detected within the resolution of the measurement, confirming single-phase *β*-Ga_2_O_3_ growth. In contrast, for the non-optimized film, the intensities of the *β*-Ga_2_O_3_ ($\bar{4}02$) reflections are about one order of magnitude weaker and barely emerge from the background, indicating poor crystalline quality. This clear enhancement of the *β*-Ga_2_O_3_ peak intensities alongside the reduction in *E*_U_ indicates a strong correlation between optical disorder and the crystalline quality in the sputtered films. The rocking curve of the *β*-Ga_2_O_3_ ($\bar{2}01$) reflection (Fig. 3c) is well described by a Lorentzian profile with a FWHM of 1.78°. For comparison, various CVD-based and MBE-based techniques report FWHM values in the range of ≈ 0.33°–2.0° for *β*-Ga_2_O_3_ films on C-plane Al_2_O_3_ (Table 1). Our sputter-grown film with a FWHM of 1.78° thus lies well within the range of high-quality *β*-Ga_2_O_3_ grown by CVD- and MBE-based techniques, even though it is obtained by RF magnetron sputtering rather than a dedicated and complex epitaxial reactor or ultrahigh vacuum.

The optimized film exhibits well-defined heteroepitaxial registry with the C-plane Al_2_O_3_ substrate, as evidenced by in-plane XRD and atomic-resolution scanning transmission electron microscopy (STEM) analyses. To reveal the in-plane orientation of the *β*-Ga_2_O_3_ film relative to the C-plane Al_2_O_3_ substrate, the XRD-*ϕ* scan was performed using the *β*-Ga_2_O_3_ ($\bar{4}01$) reflection (Fig. 3d). The *ϕ* scan exhibits six peaks separated by 60°, indicating heteroepitaxial in-plane alignment on C-plane Al_2_O_3_. Considering the twofold symmetry of monoclinic *β*-Ga_2_O_3_, these six peaks correspond to three equivalent in-plane rotational domains of the ($\bar{2}01$)-oriented *β*-Ga_2_O_3_ film. Such multi-domain in-plane epitaxy on C-plane Al_2_O_3_ has also been reported for high-quality heteroepitaxial *β*-Ga_2_O_3_ grown by CVD-based methods, consistent with the established heteroepitaxial relationship *β*-Ga_2_O_3_ [102]||Al_2_O_3_ ($1\bar{1}20$)^49,56^. Figure 3e shows an atomic-resolution high-angle annular dark-field (HAADF)-STEM image of one of these in-plane domains, and the detailed multi-domain in-plane epitaxial structure is further confirmed in Supplementary Note 5. Whereas previous RF-sputtered Ga_2_O_3_ films on C-plane Al_2_O_3_ have largely been limited to amorphous, polycrystalline, or strongly textured films^35–37^, achieving a single-phase *β*-Ga_2_O_3_ epilayer with a low *E*_U_ and narrow rocking-curve FWHM thus marks a substantial advance in sputter-based growth.

In addition to improved optical and crystallographic quality, the *E*_U_-optimized *β*-Ga_2_O_3_ film also shows a flat surface, with a root mean square (RMS) roughness of 1.15–1.27 nm (Fig. 3f). This value is comparable to the lower end of the RMS roughness reported for heteroepitaxial *β*-Ga_2_O_3_ films grown by CVD- and MBE-based techniques (typically 0.21–7 nm, Table 1). The achieved surface smoothness is expected to be beneficial for heteroepitaxial device fabrication, where reduced interface roughness can help mitigate carrier scattering and improve breakdown and reliability.

### Homoepitaxial growth on ***β*-Ga**_**2**_**O**_**3**_ substrates

**Fig. 4:**
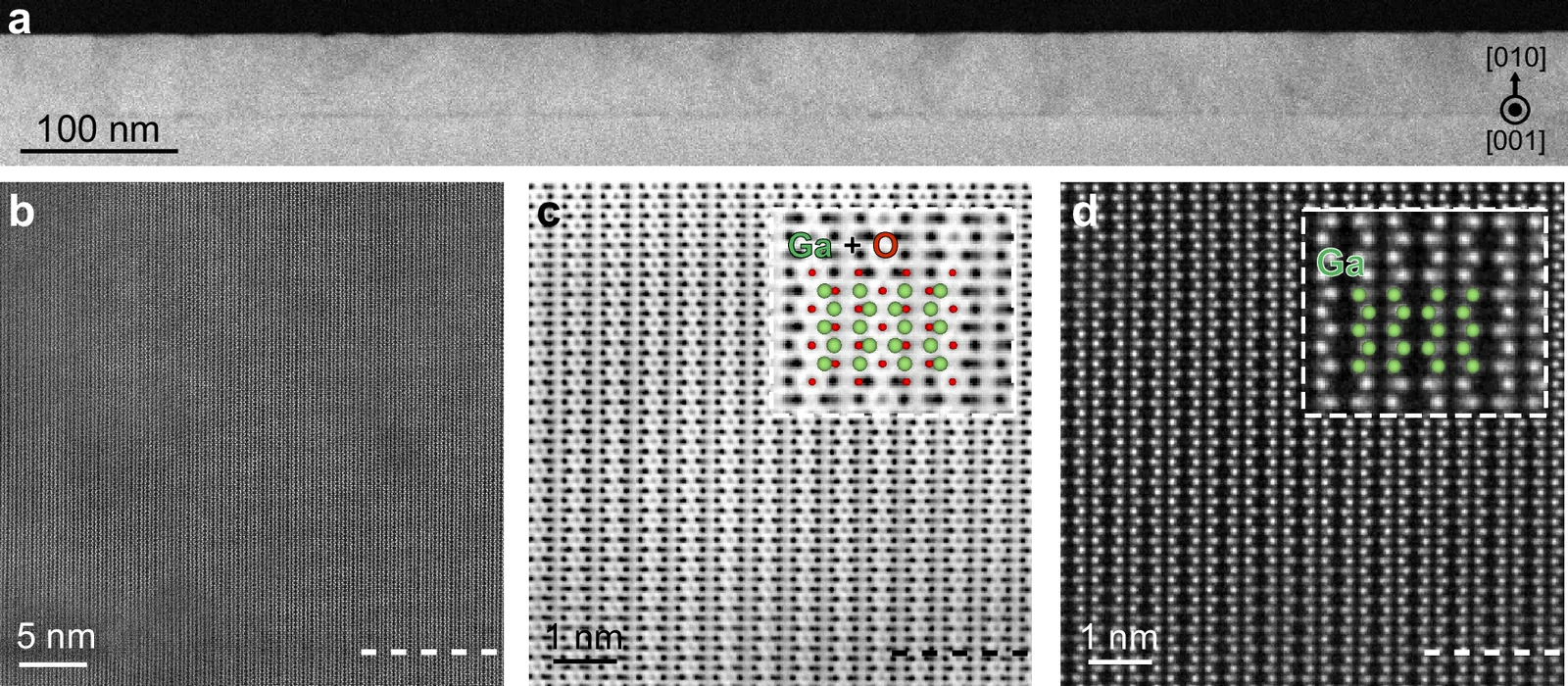
Homoepitaxial *β*-Ga_2_O_3_ grown under the optimized condition. **a** HAADF-STEM image of the homoepitaxial *β*-Ga_2_O_3_ film viewed along the [001] direction. **b** Magnified HAADF-STEM image near the interface in (**a**). Magnified **c** ABF- and **d** HAADF-STEM images near the interface in (**b**). In (**b**-**d**), dashed lines indicate the interface. The insets in (**c**) and (**d**) represent an atomic-resolution magnified image with Ga-occupied (green spheres) and O-occupied (red spheres) columns. Similar homoepitaxial structures were observed by STEM at three spatially separated locations of the same film.

To examine whether the BO-optimized window identified for heteroepitaxy also captures growth trends relevant to homoepitaxy, we applied the same window to *β*-Ga_2_O_3_ substrates and demonstrated single-crystal *β*-Ga_2_O_3_ homoepitaxy by sputtering. A low-magnification cross-sectional HAADF-STEM image (Fig. 4a) reveals a laterally uniform *β*-Ga_2_O_3_ film. A higher-magnification HAADF-STEM image (Fig. 4b) shows a continuous array of well-ordered atomic columns without grain boundaries or rotational domain boundaries, indicating single-crystal homoepitaxial growth. Atomic-resolution STEM images in annular bright-field (ABF) (Fig. 4c) and HAADF (Fig. 4d) modes further resolve the *β*-Ga_2_O_3_ cation and anion sublattices with excellent ordering: in the ABF image, both Ga and O columns are visible, whereas in the HAADF image, the heavier Ga columns dominate the contrast, with the projected lattice matching the expected crystal structure of *β*-Ga_2_O_3_^67^. These observations show that the self-driving optimization performed on C-plane Al_2_O_3_ directly identifies a growth window that also enables single-crystal *β*-Ga_2_O_3_ homoepitaxy by sputtering without any additional optimization, establishing a new capability for sputter epitaxy.

AFM observations further confirm a smooth, pit-free surface for the homoepitaxial film: the RMS roughness is 1.10–1.25 nm (Supplementary Fig. 6), which is favorable for subsequent doping/contact processing and for minimizing interface scattering in device heterostructures. The electrical properties of the homoepitaxial *β*-Ga_2_O_3_ films were evaluated using simple two-terminal test structures. Unintentionally doped *β*-Ga_2_O_3_ layers with a thickness of 55 nm were grown under the optimized conditions, and Ti (20 nm)/Au (150 nm) contacts were deposited on the film surface, followed by rapid thermal annealing at 500 °C for 1 min in N_2_, which is a standard technique to form Ohmic contacts to *β*-Ga_2_O_3_^68,69^. However, the device resistance remained beyond the measurement range (10 GΩ or higher) over the applied voltage range up to 10 V, indicating that the optimized sputtered films are very lightly doped. This low unintentional doping level, consistent with the absence of extended defects (e.g., grain boundaries or secondary phases) in the STEM observations, is promising for applications where semi-insulating behavior is desired.

### Interpretable random-forest analysis

**Fig. 5:**
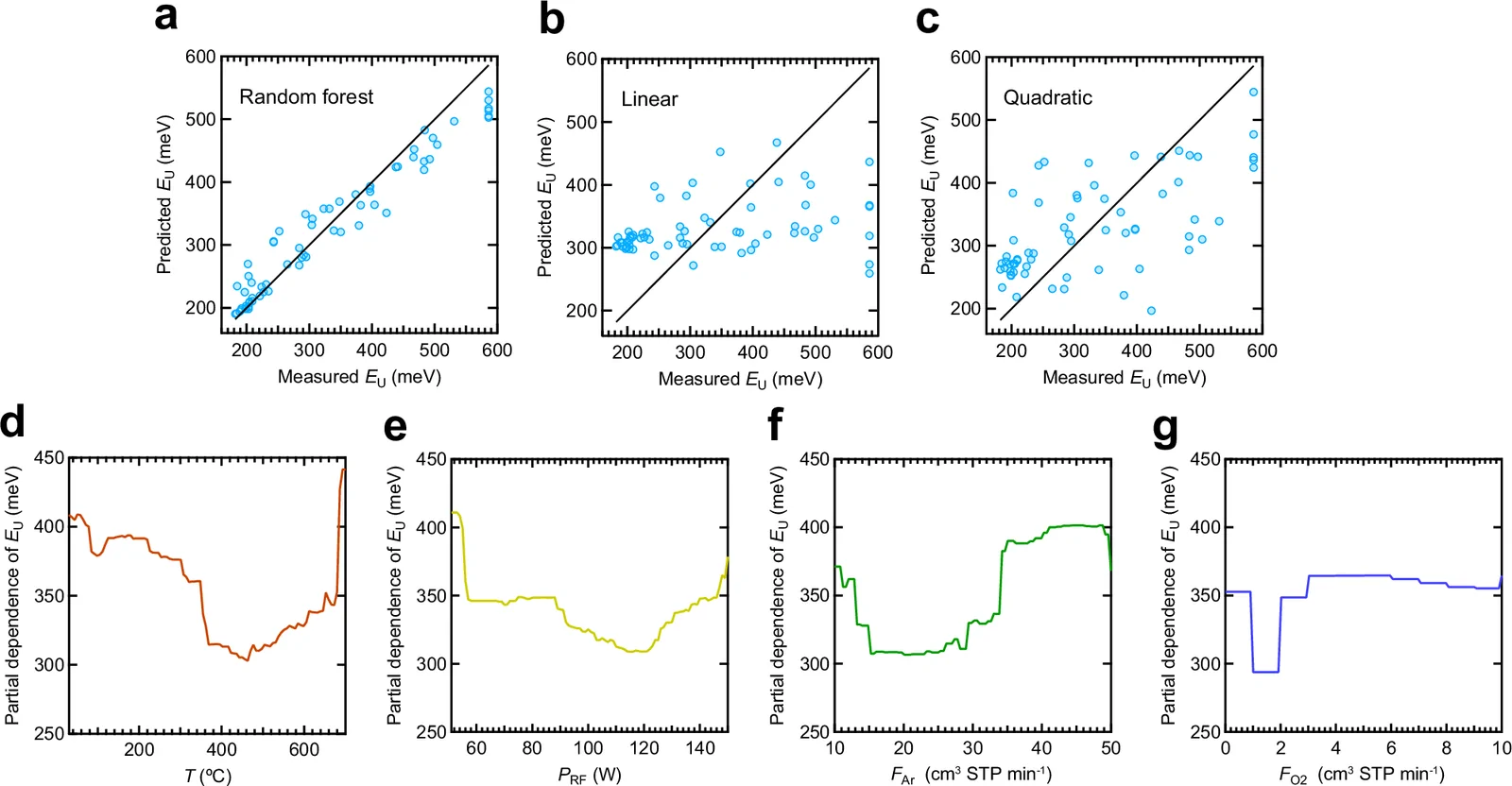
Explainable random forest analysis for single-parameter effects on *E*_U_. **a**–**c** Predicted versus measured *E*_U_ for three regression models trained on the experimental 66 sample dataset: **a** random forest, **b** linear, and **c** quadratic. The diagonal line indicates perfect agreement. 1D PDPs from the random-forest model showing the marginal effect of **d** *T*, **e** ${P}_{{{{\rm{RF}}}}}$, **f** ${F}_{{{{\rm{Ar}}}}}$, and **g** ${F}_{{{{{\rm{O}}}}}_{2}}$ on *E*_U_.

To move beyond black-box optimization and identify which growth parameters dominate the *E*_U_ landscape and how they interact, we next analyze the growth-parameter space explored by the self-driving campaign using an explainable surrogate model. Specifically, we constructed a random-forest surrogate^70–73^ that reproduces the measured *E*_U_ landscape over the BO-explored parameter region and performed a suite of analyses to extract salient structure and distill the model into a compact, explainable representation (see Methods section “Explainable surrogate decomposition using random forests”). The random forest surrogate trained on all 66 growth runs (including the optimum at the 56th run) effectively captures the observed *E*_U_ variations across the four-dimensional growth-parameter space, achieving an in-sample coefficient of determination *R*^2^ = 0.92 with a root-mean-square error (RMSE) of 36 meV (Fig. 5a), whereas linear and quadratic polynomial regressions yield much poorer performance [*R*^2^ = 0.11, RMSE = 121 meV for the linear model (Fig. 5b); *R*^2^ = 0.41, RMSE = 98.9 meV for the quadratic model (Fig. 5c)]. Taken together, these comparisons show that classical linear or quadratic regression is insufficient to capture the complex dependence of *E*_U_ on the sputter-growth parameters, whereas the BO-discovered behavior can be summarized for interpretation by a compact, data-driven surrogate that we can interrogate to reveal the underlying growth trends.

To visualize how each parameter influences *E*_U_ more intuitively, we computed one-dimensional (1D) partial dependence plots (PDPs) of the random forest model (Fig. 5d–g). A PDP provides a regime-averaged *E*_U_-parameter response curve predicted by the surrogate model, obtained by sweeping one parameter across its range while averaging over the remaining high-dimensional parameter space^70,74^ (see Methods section “Explainable surrogate decomposition using random forests”). These PDPs reveal that *E*_U_ exhibits a pronounced minimum in an intermediate range for each knob: intermediate *T* around 360–540 °C, ${P}_{{{{\rm{RF}}}}}$ near 100–125 W, ${F}_{{{{\rm{Ar}}}}}$ around 15–30 cm^3^ STP min^−1^, and a small but finite ${F}_{{{{{\rm{O}}}}}_{2}}$ of 1–2 cm^3^ STP min^−1^. In contrast to ${F}_{{{{{\rm{O}}}}}_{2}}$, which requires fine tuning at the 1 cm^3^ STP min^−1^ level, the PDP for ${F}_{{{{\rm{Ar}}}}}$ shows a broad low-*E*_U_ plateau spanning ≈ 15–30 cm^3^ STP min^−1^. This indicates that precise adjustment of the Ar flow is not critical as long as extreme under- or over-supply is avoided. Moving too far away from these intermediate values in any direction leads to an increase in *E*_U_. In this sense, the random forest analysis provides a compact, human-interpretable summary of the trends learned implicitly by BO during the closed-loop optimization.

**Fig. 6:**
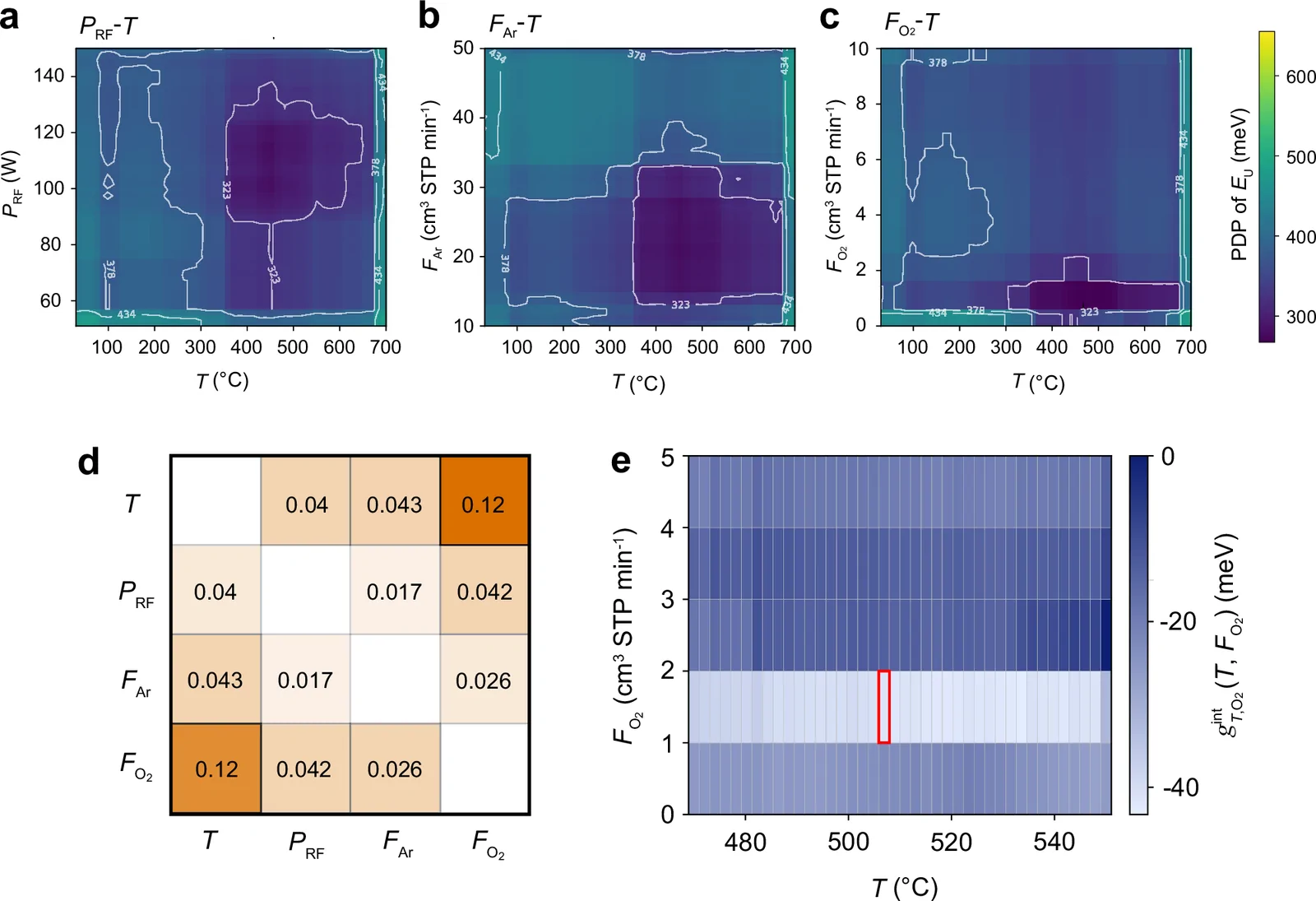
Visualizing and quantifying pairwise interactions in the *E*_U_ landscape. 2D PDPs of *E*_U_ as a function of *T* and each other parameter: **a** ${P}_{{{{\rm{RF}}}}}$, **b** ${F}_{{{{\rm{Ar}}}}}$, and **c** ${F}_{{{{{\rm{O}}}}}_{2}}$. **d** Friedman’s *H*^2^-statistic pairwise matrix, quantifying the magnitude of non-additive effects between parameters. **e** ${g}_{T,{{{{\rm{O}}}}}_{2}}^{{{\mathrm{int}}}}(T,{F}_{{{{{\rm{O}}}}}_{2}})$ in the restricted window around the optimum (*T* = 470–550 °C, ${F}_{{{{{\rm{O}}}}}_{2}}=$0−5 cm^3^ STP min^−1^). In (**e**), the red rectangle marks the region corresponding to the optimized condition.

To further examine pairwise interactions between growth parameters, we computed two-dimensional (2D) PDPs as functions of substrate temperature and one additional parameter (*T*-*P*_RF_, *T*-*F*_Ar_, and *T*-*F*_O2_) (Fig. 6a–c). In these 2D PDPs, the *E*_U_　response is largely described by the superposition of the corresponding 1D trends, with a low-*E*_U_ region appearing at intermediate *T* (360–540 °C). Varying RF power or gas flow mainly shifts the response without strongly reshaping this low-*E*_U_ region, indicating weak interactions and an approximately additive response. We quantify this near-additive behavior by constructing an explicitly additive approximation of the random forest surrogate, where the predicted *E*_U_ is expressed as the sum of four 1D PDPs;

1 $${E}_{{{{\rm{U}}}}}^{{{{\rm{add}}}}}(T,{P}_{{{{\rm{RF}}}}},{F}_{{{{\rm{Ar}}}}},{F}_{{{{{\rm{O}}}}}_{2}})\approx {g}_{T}(T)+{g}_{P}({P}_{{{{\rm{RF}}}}})+{g}_{{{{\rm{Ar}}}}}({F}_{{{{\rm{Ar}}}}})+{g}_{{{{{\rm{O}}}}}_{2}}({F}_{{{{{\rm{O}}}}}_{2}})+{c}_{0}$$

where ${g}_{T}(T)$, ${g}_{P}({P}_{{{{\rm{RF}}}}})$, ${g}_{{{{\rm{Ar}}}}}({F}_{{{{\rm{Ar}}}}})$, and ${g}_{{{{{\rm{O}}}}}_{2}}({F}_{{{{{\rm{O}}}}}_{2}})$ are the 1D PDPs of the random forest surrogate with respect to each growth parameter. The constant ${c}_{0}$ was chosen to minimize the mean-squared error of Eq. (1) against the full random-forest predictions over the *N* = 66 BO-acquired samples. This additive PDP approximation [Eq. (1)] preserves much of the structure learned by the full random forest model, reproducing its outputs with *R*^2^ = 0.84 (and explaining *R*^2^ = 0.69 of the variance in the measured *E*_U_). These results indicate that most of the four-dimensional *E*_U_ landscape is governed by approximately independent, additive contributions of the four knobs (*T*, ${P}_{{{{\rm{RF}}}}}$, ${F}_{{{{\rm{Ar}}}}}$, and ${F}_{{{{{\rm{O}}}}}_{2}}$), while the remaining discrepancy reflects non-additive interactions and/or higher-order structure.

To quantify the remaining non-additive component beyond this largely additive structure, we computed pairwise interaction strengths using Friedman’s *H*^2^-statistic^75^ from the random forest surrogate (see Methods section “Explainable surrogate decomposition using random forests”). For a given pair of parameters, *H*^2^ quantifies the fraction of the 2D-PDPs’ variation that cannot be explained by the sum of the two corresponding 1D PDPs; *H*^2^ = 0 corresponds to a purely additive dependence with no interaction, whereas *H*^2^ = 1 indicates that non-additive interactions dominate. In our case, all interaction fractions *H*^2^ were found to be 0.12 or lower (Fig. 6d), confirming that interaction effects are generally weak across most parameter pairs. The strongest interactions occur for the $T-{F}_{{{{{\rm{O}}}}}_{2}}$ pair (*H*^2^ = 0.12), while pairs involving RF power and Ar gas flow show weaker interactions (*H*^2^ = 0.017–0.043). Taken together with the additive approximation in Eq. (1), these results motivate a compact and interpretable representation of the random forest surrogate in which the response is predominantly additive, with the dominant residual non-additivity confined to the *T*-${F}_{{{{{\rm{O}}}}}_{2}}$ pair:

2 $${E}_{{{{\rm{U}}}}}^{{{{\rm{total}}}}}\left(T,{P}_{{{{\rm{RF}}}}},{F}_{{{{\rm{Ar}}}}},{F}_{{{{{\rm{O}}}}}_{2}}\right)=	{g}_{T}\left(T\right)+{g}_{P}\left({P}_{{{{\rm{RF}}}}}\right)+{g}_{{{{\rm{Ar}}}}}\left({F}_{{{{\rm{Ar}}}}}\right)+{g}_{{{{{\rm{O}}}}}_{2}}\left({F}_{{{{{\rm{O}}}}}_{2}}\right) \\ 	+{g}_{T,{O}_{2}}^{{{\mathrm{int}}}}(T,{F}_{{O}_{2}})+{c}_{1}$$

where *c*_1_ is an offset that absorbs the arbitrary baselines. The interaction term ${g}_{T,{{{{\rm{O}}}}}_{2}}^{{{\mathrm{int}}}}(T,{F}_{{{{{\rm{O}}}}}_{2}})$ is defined as the pure two-dimensional interaction component extracted from the 2D PDP as follows:

3 $${g}_{T,{{{{\rm{O}}}}}_{2}}^{{{\mathrm{int}}}}(T,{F}_{{{{{\rm{O}}}}}_{2}})={g}_{T,{{{{\rm{O}}}}}_{2}}(T,{F}_{{{{{\rm{O}}}}}_{2}})-{g}_{{{{{\rm{O}}}}}_{2}}({F}_{{{{{\rm{O}}}}}_{2}})-{g}_{T}\left(T\right)+{c}_{T,{F}_{{{{{\rm{O}}}}}_{2}}}$$

where ${g}_{T,{{{{\rm{O}}}}}_{2}}(T,{F}_{{{{{\rm{O}}}}}_{2}})$ denotes the 2D PDP of the random forest surrogate with respect to *T* and ${F}_{{{{{\rm{O}}}}}_{2}}$, and ${c}_{T,{F}_{{{{{\rm{O}}}}}_{2}}}$ is the constant chosen such that $\left\langle {g}_{T,{{{{\rm{O}}}}}_{2}}^{{{\mathrm{int}}}}(T,{F}_{{{{{\rm{O}}}}}_{2}})\right\rangle=0$ over the 2D PDP grid. Incorporating this single interaction term increases the fidelity to the full random forest predictions, evaluated at the *N* = 66 BO-acquired sample points, to *R*^2^ = 0.90 and yields *R*^2^ = 0.75 against the measured *E*_U_, highlighting that the *E*_U_ landscape admits an interpretable form: the effects of the parameters are largely additive, with only weak pairwise couplings for most parameter pairs (*H*^2^ = 0.017–0.043). The only appreciable deviation from additivity is a modest $T-{F}_{{{{{\rm{O}}}}}_{2}}$ interaction term (*H*^2^ = 0.12), consistent with coupled thermodynamic/kinetic effects (e.g., changes in oxygen chemical potential, surface stoichiometry, and defect incorporation)^76,77^ that are not captured by one-dimensional trends alone.

Figure 6e visualizes the role of the $T-{F}_{{{{{\rm{O}}}}}_{2}}$ interaction around the optimum by plotting ${g}_{T,{{{{\rm{O}}}}}_{2}}^{{{\mathrm{int}}}}(T,{F}_{{{{{\rm{O}}}}}_{2}})$ in the restricted window. The interaction term reduces *E*_U_ along the same $T-{F}_{{{{{\rm{O}}}}}_{2}}$ ridge where *E*_U_ attains its minimum. Thus, in addition to the nearly additive 1D contributions, a modest $T-{F}_{{{{{\rm{O}}}}}_{2}}$ interaction further reduces *E*_U_ by ≈ 40 meV near the optimal growth conditions, suggesting a possible coupled thermodynamic/kinetic contribution beyond independent one-dimensional trends^76,77^.

These insights suggest a simple, human-executable optimization strategy for sputter epitaxy of *β*-Ga_2_O_3_ that mirrors the behavior learned by the self-driving system. Starting from an arbitrary but reasonable set of growth conditions, the largely additive structure of the growth-parameter space indicates that optimization can proceed efficiently through sequential one-dimensional tuning of individual parameters. In practice, one parameter is varied at a time to minimize *E*_U_, while the parameters optimized in the preceding steps are fixed at their current optimized values. This corresponds to first adjusting the substrate temperature, followed by RF power, Ar flow, and O_2_ flow. Because each parameter exhibits a largely independent, well-behaved optimum, this sequential procedure brings the process close to the low-*E*_U_ optimum within the explored parameter space. From this neighborhood, a focused two-dimensional optimization in the $T-{F}_{{{{{\rm{O}}}}}_{2}}$ plane is sufficient to capture the residual non-additive contribution. In this way, the BO-driven search of a black-box process–property response, together with the random forest-based analysis, is translated into a concrete, human-executable optimization strategy that can be implemented without an autonomous platform. More broadly, these results demonstrate that the self-driving sputtering framework not only identifies high-quality growth conditions, but also generates a dataset rich enough to infer an interpretable process landscape and actionable growth rules.

### Rule-guided human re-optimization

**Fig. 7:**
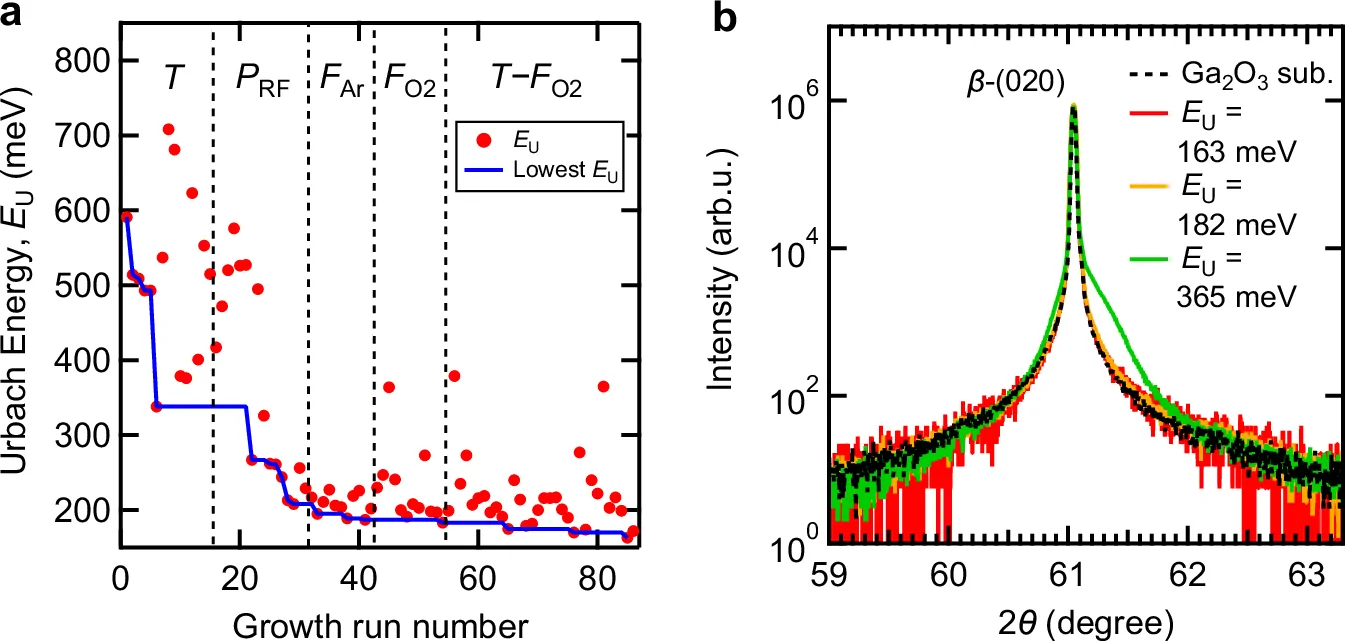
Human-executed re-optimization and XRD characterization of homoepitaxial films. **a** A human experimenter performed sequential 1D optimization of *T*, *P*_RF_, *F*_Ar_, and *F*_O2_, followed by 2D fine-tuning in the *T*-O_2_ plane. The vertical dashed lines indicate the boundaries between the optimization stages for *T*, *P*_RF_, *F*_Ar_, *F*_O2_, and *T*-*F*_O2_, respectively. Red circles represent the experimental *E*_U_ as a function of growth run number. The blue line indicates the lowest *E*_U_ achieved up to each run. **b** XRD *θ*-2*θ* scans of the *β*-(020) reflection for the *β*-Ga_2_O_3_ homoepitaxial films grown under low-*E*_U_ (163 and 182 meV) and high-*E*_U_ (365 meV) conditions, together with the substrate. Source data are provided as a Source Data file.

To examine whether the extracted growth rule can practically guide process optimization, we performed human-executed re-optimization following the optimization strategy described above. This re-optimization was performed manually by a human experimenter without using BO or another machine-learning optimizer. The initial one-dimensional searches were carried out within the same four-dimensional parameter bounds as those used in the original BO campaign. Specifically, ${P}_{{{{\rm{RF}}}}}$, ${F}_{{{{\rm{Ar}}}}}$, and ${F}_{{{{{\rm{O}}}}}_{2}}$ were initially fixed at 100 W, 30 cm^3^ STP min^−1^, and 5 cm^3^ STP min^−1^, respectively, and *T* was swept over the allowed range. The experimenter then sequentially swept ${P}_{{{{\rm{RF}}}}}$, ${F}_{{{{\rm{Ar}}}}}$, and ${F}_{{{{{\rm{O}}}}}_{2}}$ while fixing the previously optimized parameters. The number of trials in each one-dimensional sweep was determined empirically by the experimenter based on the observed *E*_U_ trends, rather than by a machine-learning model. This sequential 1D procedure reached *E*_U_ = 183 meV at (*T*, ${P}_{{{{\rm{RF}}}}}$, ${F}_{{{{\rm{Ar}}}}}$, ${F}_{{{{{\rm{O}}}}}_{2}}$) = (512 °C, 97 W, 26.5 cm^3^ STP min^−1^, 0.7 cm^3^ STP min^−1^) after 58 runs (Fig. 7a), nearly reproducing the original BO optimum level. Starting from this low-*E*_U_ condition, we then performed focused two-dimensional refinement in the *T*–*F*_O2_ plane, as suggested by the residual interaction identified in the surrogate analysis. In this final step, *T* and *F*_O2_ were explored within restricted ranges of 462–612 °C and 0–1.5 cm^3^ STP min^−1^, respectively. This focused refinement reached the original BO optimum level of *E*_U_ ≤ 182 meV after 65 runs and eventually achieved a minimum *E*_U_ of 163 meV after 85 runs (Fig. 7a), which is 19 meV lower than the original BO optimum. Thus, the extracted human-interpretable strategy did not merely describe correlations in the BO-acquired dataset, but provided an experimentally executable protocol that guided targeted optimization beyond the original BO optimum.

This human-executed re-optimization also provides a practical benchmark for evaluating the efficiency of the self-driving workflow. The original BO campaign reached the original optimum level of *E*_U_ = 182 meV by the 56th run, corresponding to ≈ 8 working days under the throughput of the present SDL workflow (7–8 runs per day) (Supplementary Note 1). By contrast, the human-executed strategy required 65 runs to reach the same threshold of *E*_U_ ≤ 182 meV. Under a conventional non-automated workflow, in which sputter growth and optical characterization are performed manually at a throughput of ≈ 3 runs per day, this would correspond to ≈ 22 working days. Thus, relative to this rule-guided but non-automated benchmark, the present SDL workflow reduces the working time required to reach the same *E*_U_ threshold by a factor of ≈ 2.7, with the gain arising primarily from automation-enabled throughput rather than from a large reduction in the number of experimental runs. This comparison is conservative, because the human-executed strategy already benefits from the growth rule distilled from the BO-acquired dataset; human-executed unguided trial-and-error would likely require substantially more experimental effort.

Having obtained a second, independently identified low-*E*_U_ condition through human-executed re-optimization, we next tested whether the correspondence between the heteroepitaxial *E*_U_ landscape and homoepitaxial film quality extends beyond a single BO-optimized recipe. For this purpose, we compared homoepitaxial films grown under two independently identified low-*E*_U_ conditions on C-plane Al_2_O_3_: the BO-identified condition, (507 °C, 119 W, 26 cm^3^ STP min^−1^, 1 cm^3^ STP min^−1^), which yielded *E*_U_ = 182 meV, and the human-reoptimized condition, (562 °C, 97 W, 26.5 cm^3^ STP min^−1^, 1 cm^3^ STP min^−1^), which yielded *E*_U_ = 163 meV. XRD *θ*–2*θ* scans show that homoepitaxial films grown under both low-*E*_U_ conditions exhibit sharp *β*-Ga_2_O_3_ (020) reflections that are indistinguishable from the substrate peak, indicating high-quality homoepitaxial growth (Fig. 7b). In contrast, a homoepitaxial film grown under an off-window condition, (612 °C, 97 W, 26.5 cm^3^ STP min^−1^, 0 cm^3^ STP min^−1^), which corresponded to a much larger *E*_U_ = 365 meV on C-plane Al_2_O_3_, exhibited a broad *β*-Ga_2_O_3_ (020) peak (Fig. 7b), indicating degraded crystalline quality. These results show a correspondence between the heteroepitaxial *E*_U_ landscape and homoepitaxial crystalline quality: low-*E*_U_ conditions on C-plane Al_2_O_3_ yield high-quality homoepitaxial films, whereas an off-window high-*E*_U_ condition leads to degraded homoepitaxial growth, thereby supporting the cross-substrate relevance of the extracted growth rule within the tested process space.

### Higher-fidelity surrogate retains the growth-rule structure

**Fig. 8:**
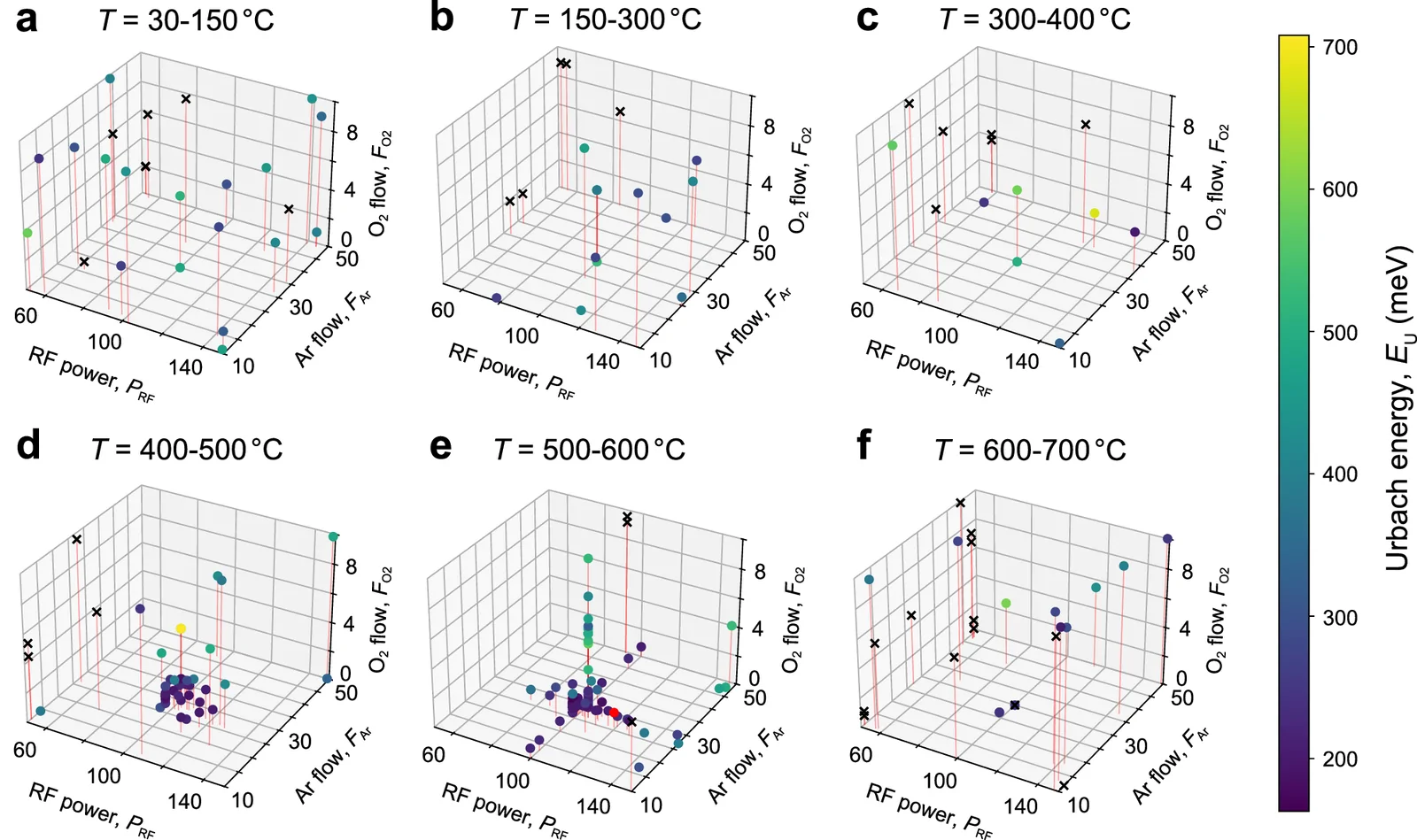
Experimental *E*_U_ distribution in the expanded 215-run dataset. The experimental *E*_U_ in the three-dimensional parameter space ${P}_{{{{\rm{RF}}}}}-{F}_{{{{\rm{Ar}}}}}-{F}_{{{{{\rm{O}}}}}_{2}}$ for different *T* windows of **a** 30−150 °C, **b** 150–300 °C, **c** 300–400 °C, **d** 400–500 °C, **e** 500-600 °C, and **f** 600–700 °C. Marker color represents *E*_U_ (color bar, meV), visualizing the distribution of the BO-acquired, additional sampling, and rule-guided re-optimization data. In (**e**), the red circle represents the lowest *E*_U_ (163 meV) obtained in the rule-guided re-optimization. Black crosses indicate runs for which the *E*_U_ could not be extracted (NaN).

Finally, we examined whether the growth-rule structure extracted from the original BO-acquired dataset is retained when the response-surface analysis is performed using a higher-fidelity surrogate trained on an expanded dataset. The original random-forest surrogate was based on 66 samples acquired during the closed-loop BO campaign. Because BO samples the growth-parameter space non-uniformly by design, we expanded the dataset to 215 growth runs to reduce the dependence of the analysis on the original BO acquisition path. The expanded dataset consisted of the original 66 BO-acquired runs, 86 human-executed rule-guided re-optimization runs, and 63 additional space-filling runs selected to maximize the minimum distance from the existing data points in the normalized four-dimensional parameter space. Compared with the BO-acquired dataset shown in Fig. 2b–g, the expanded dataset more broadly covers the growth-parameter space across the temperature windows, providing a denser basis for response-surface analysis (Fig. 8).

Training a random-forest surrogate on the expanded 215-run dataset improved the predictive fidelity of the response-surface model, yielding an in-sample *R*^2^ of 0.96 and a 5-fold cross-validated *R*^2^ of 0.65. This cross-validated performance is substantially higher than that obtained for the original 66-run BO-acquired dataset (*R*^2^ ≈ 0.43), reflecting the denser and less acquisition-biased sampling of the expanded dataset. We first evaluated the impurity-based feature importance of the expanded-dataset random forest, which quantifies how strongly each input parameter contributes to reducing the prediction error across the ensemble of decision trees^70,72^. This analysis showed that *F*_O2_ has the largest importance (0.36), indicating that introducing and finely adjusting a small amount of oxygen is critical for accessing the low-*E*_U_ region (Supplementary Fig. 8a). The corresponding 1D PDPs show trends similar to those inferred from the original BO-acquired dataset (Fig. 5d–g): low-*E*_U_ regions appear at intermediate *T*, intermediate *P*_RF_, moderate *F*_Ar_, and low but finite *F*_O2_, whereas deviations from these ranges increase *E*_U_ (Supplementary Fig. 8b-e).

**Fig. 9:**
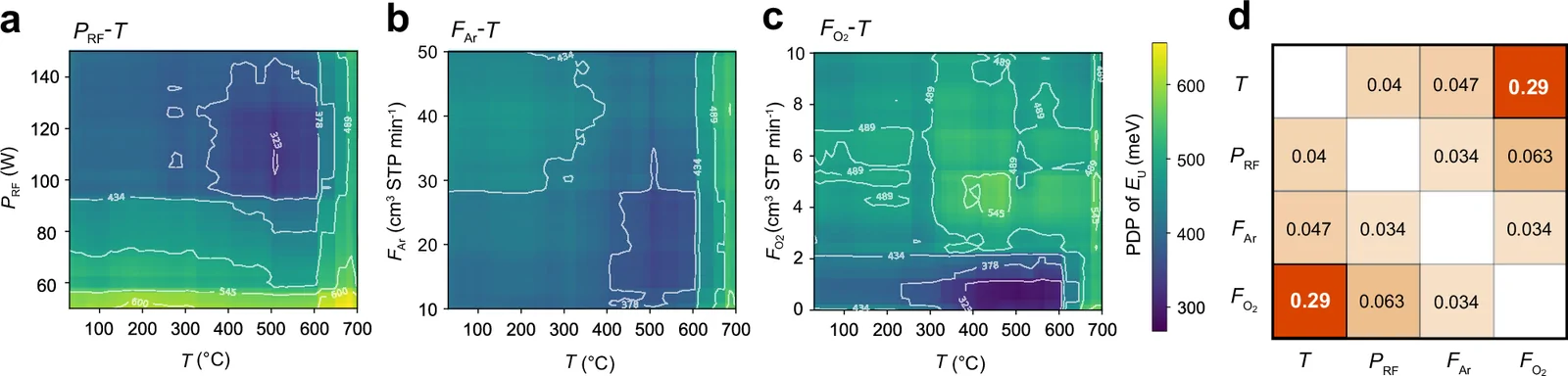
Pairwise interactions in the higher-fidelity expanded-dataset surrogate. 2D PDPs of *E*_U_ obtained from the random-forest surrogate trained on the expanded 215-run dataset as a function of *T* and each other parameter: **a** ${P}_{{{{\rm{RF}}}}}$, **b** ${F}_{{{{\rm{Ar}}}}}$, and **c** ${F}_{{{{{\rm{O}}}}}_{2}}$. **d** Friedman’s *H*^2^-statistic pairwise matrix obtained from the expanded-dataset surrogate, quantifying the magnitude of non-additive effects between parameters.

We then repeated the same partial-dependence-based decomposition and interaction analysis using the expanded-dataset surrogate. Compared with the 2D PDPs obtained from the original 66-run BO-acquired dataset, the *T*-*P*_RF_ and *T*-*F*_Ar_ PDPs of the expanded-dataset surrogate remain largely additive, consistent with a superposition of the corresponding 1D PDPs (Fig. 9a, b). By contrast, the *T-F*_O2_ PDP more clearly resolves localized low- and high-*E*_U_ structures, revealing a more pronounced residual non-additive structure in the higher-fidelity surrogate (Fig. 9c). Quantitatively, the additive-only approximation constructed from the 1D PDPs using Eq. (1) reproduced a substantial fraction of the full random-forest predictions but still left a residual discrepancy (*R*^2^ = 0.72). Adding a single *T*-*F*_O2_ interaction term using Eq. (2) improved the approximation to *R*^2^ = 0.85. Consistently, Friedman’s *H*^2^ interaction analysis identified the *T*-*F*_O2_ pair as the dominant interaction, with *H*^2^ = 0.29, whereas all other pairwise interactions were much weaker, with *H*^2^ ≤ 0.063 (Fig. 9d). Taken together, the analysis of a higher-fidelity surrogate using the additional dataset consistently supports that the response-surface structure has predominantly additive single-parameter contributions with a notable residual *T*-*F*_O2_ interaction. Combined with the human-executed re-optimization and controlled homoepitaxial validation described above, these results demonstrate that the self-driving workflow converts autonomous optimization data into validated, human-usable process knowledge, thereby moving self-driving thin-film synthesis beyond recipe discovery toward interpretable process engineering.

## Discussion

In this work, we demonstrate that RF magnetron sputtering, when combined with an interpretable self-driving growth framework and automated optical analyses, can produce single-phase heteroepitaxial *β*-Ga_2_O_3_ films with high crystallographic and optical quality approaching that typically associated with CVD- and MBE-based epitaxy. Using the *E*_U_ as a quantitative objective, the closed-loop campaign achieved *E*_U_ = 182 meV, below the lowest previously reported value for sputtered *β*-Ga_2_O_3_ films (280 meV)^47^ and below a representative MOCVD-grown report (220 meV)^46^ (Table 1). The rule-guided human re-optimization further reduced *E*_U_ to 163 meV. While this performance remains above the best reported epitaxial films (~150 meV)^48,59^ and bulk single crystals (60–140 meV)^42^, the residual gap provides a clear roadmap for further improvement, namely, suppressing sub-gap disorder/defect states that are still detectable optically even in single-crystal films. Importantly, the same BO-identified growth window discovered on C-plane Al_2_O_3_ was also sufficient to enable single-crystal *β*-Ga_2_O_3_ homoepitaxy. Given that RF sputtering is a mature, low-operating-cost technology already used for large-area oxide deposition in semiconductor and display manufacturing, these results point to self-driving sputter epitaxy as a promising, industry-compatible route for ultra-wide-bandgap *β*-Ga_2_O_3_ epitaxial layers and devices.

Although the initial dataset in our campaign happened to include a sample with a moderately low *E*_U_ (<350 meV), BO is not predicated on starting from a near-optimal recipe. Instead, the exploration-exploitation trade-off encourages broad sampling early on while progressively refining around promising regions. Consistent with this behavior, the *E*_U_ values span a wide range across runs while the best-achieved *E*_U_ decreases steadily. In our self-driving sputtering system, we further improved robustness against local trapping by adopting an adaptive prior-mean strategy, in which the GP prior mean is resampled at each iteration from a uniform distribution bounded by the minimum and maximum observed *E*_U_ values^45^. This design promotes exploration of previously unseen regions and helps the search escape locally optimal regimes, and its effectiveness has been validated in benchmark simulations^45^.

A distinctive aspect of this study is that the self-driving workflow does not simply identify an optimized recipe, but also converts the closed-loop data into human-usable growth rules. While BO efficiently discovers growth conditions that minimize *E*_U_, process development ultimately requires an interpretable understanding of which parameters matter and how they should be adjusted. To bridge autonomous search and human-intuitive tuning, we analyzed the growth-parameter landscape explored by BO and distilled the closed-loop dataset into actionable process knowledge. Using a random forest as an explainable local surrogate, we identified a near-additive *E*_U_ landscape with a dominant residual interaction between temperature and O_2_ flow, rather than a fully entangled four-dimensional response. This structure yields a practical tuning logic consisting of sequential one-dimensional adjustments followed by a focused two-dimensional refinement in temperature and O_2_ flow. The validity of this rule as an optimization strategy was supported by human-executed re-optimization, and its relevance to homoepitaxy was further examined by controlled cross-substrate tests within the tested process space.

The generality of these results should be considered at two levels: the specific growth rule extracted for *β*-Ga_2_O_3_ sputtering and the broader workflow for converting closed-loop data into process knowledge. The numerical optimum identified here is specific to the present material and tool. However, the extracted rule structure, a predominantly additive response with a residual temperature–oxygen interaction, may provide a useful working hypothesis for other oxide thin films in which film quality is governed by a coupled balance of adatom kinetics, oxygen chemical potential, and defect formation. In such systems, mostly independent single-parameter tuning followed by focused temperature–oxygen refinement may be a practical route for process optimization. We emphasize that this surrogate analysis is not intended to establish a complete microscopic growth mechanism. Rather, it provides a data-driven basis for extracting mathematically interpretable, human-executable optimization rules. In terms of workflow generality, the same approach should be adaptable to other material systems and synthesis methods, provided that an appropriate automated quality metric can be defined. For insulating and semiconducting films with optical band gaps, automated optical spectroscopy offers a relatively direct route to such a metric, whereas metals, superconductors, or other systems without an analogous optical disorder metric would require alternative automated evaluation modules, such as X-ray diffraction or electrical transport measurements. In all cases, the reliability of the extracted rules will ultimately depend on the reproducibility of the underlying experiments and the relevance of the chosen objective metric.

Importantly, these conclusions are not specific to the choice of surrogate model. For acquisition-driven exploration, we used a GP surrogate to leverage sample efficiency and predictive uncertainty, whereas for interpretable surrogate decomposition, we used a random forest because it provided higher predictive fidelity for the experimentally sampled response surface in the present datasets (Supplementary Note 7), while also offering robustness to non-smooth responses and experimental noise together with mature explainability tools. In the original 66-run BO-acquired dataset, the random forest was used primarily as an explainable local surrogate to expose dominant trends and residual interactions supported by the closed-loop trajectory, rather than as a universal predictive model for arbitrary growth conditions. Consistent with the random forest analysis, fitting a GP with the prior mean set to the data mean produced the same qualitative picture: additive trends alone were insufficient, whereas including a single temperature and O_2_ flow interaction term substantially improved the approximation (Supplementary Note 7). The expanded 215-run dataset further strengthened this interpretation by improving the fidelity and cross-validated performance of the response-surface surrogate. Together, these results outline a general path toward interpretable SDLs, in which autonomous experimentation and surrogate-based distillation transform closed-loop optimization of black-box objective functions beyond recipe discovery into experimentally grounded, human-interpretable process knowledge.

## Methods

### Self-driving sputtering system

*β*-Ga_2_O_3_ thin films were deposited using a custom-ordered sputtering platform (ULVAC, Inc., Japan) equipped with a Ga_2_O_3_ sputtering target. 2-inch C-plane Al_2_O_3_ wafers (K&R Co., Ltd.) were used as received for heteroepitaxy, and Fe-doped semi-insulating *β*-Ga_2_O_3_ substrates (Novel Crystal Technology, Inc.) were used as received for homoepitaxy. The system was operated in a closed-loop, self-driving mode, where deposition parameters were programmatically updated between consecutive runs based on an optimization algorithm (see Methods section“Bayesian optimization with failures and adaptive prior mean”). The sputtering chamber was evacuated to a base pressure below 2 × 10^−4 ^Pa before deposition, and films were grown in an Ar/O_2_ mixture, whose flow rates were controlled by mass flow controllers. The substrate temperature was calibrated by pyrometry, and the thermocouple reading was used for temperature monitoring during growth. The target-to-substrate distance was 150 mm. Before each growth run, a target conditioning was performed by pre-sputtering for 1 min with the shutter closed. During this pre-sputtering step, *T*, *P*_RF_, *F*_Ar_, and *F*_O2_ were set to the same values as those used for the subsequent growth run. After target conditioning, the shutter was opened, and film growth was initiated under the same conditions.

The deposition time was determined for each run so as to target a nominal thickness of 55 nm, using pre-calibrated deposition-rate data measured at room temperature. Supplementary Table 1 summarizes the deposition rates calculated from the film thicknesses measured by a stylus profiler after deposition under various *P*_RF_, *F*_Ar_, and *F*_O2_ conditions. At *P*_RF_ = 100 W, the deposition rate does not depend strongly on *F*_Ar_ or *F*_O2_ and remains in the range of 0.80-0.89 nm min^−1^. By contrast, as *P*_RF_ increases from 60 to 130 W, the deposition rate increases systematically from 0.64 to 1.42 nm min^−1^. Therefore, we estimated the deposition rate from an empirical quadratic fit to the rate-versus-*P*_RF_ calibration curve and automatically determined the growth time accordingly for each run (Supplementary Fig. 2). In addition, regarding the effect of *T*, the actual thickness of the film grown under the condition (*T*, ${P}_{{{{\rm{RF}}}}}$, ${F}_{{{{\rm{Ar}}}}}$, ${F}_{{{{{\rm{O}}}}}_{2}}$) = (507 °C, 119 W, 26 cm^3^ STP min^−1^, 1 cm^3^ STP min^−1^) was estimated from STEM observation (Fig. 4) to be 56 ± 2 nm, which is in good agreement with the target nominal thickness of 55 nm. This agreement suggests that temperature-dependent changes in the effective deposition rate do not substantially affect the nominal thickness control near the optimized growth window.

The platform is equipped with a load-lock chamber capable of holding up to five 2-inch wafers. Substrate transfer between the load-lock and the deposition chamber, including transfer after film growth back to the load-lock, was automatically performed using a robotic arm.

Ex situ optical characterization was performed using a UV-Vis-NIR spectrophotometer (V-770; JASCO Corporation, Japan). After each deposition run, the sample was manually transferred from the load-lock to the optical measurement stage (in air). Once optical measurement was initiated, subsequent steps, including automated extraction of the Urbach energy (*E*_U_) from the transmittance spectra, programmatic updating of the deposition parameters, and initiation of the next growth run, were executed in an automated manner (see Methods section “Automated optical analysis”). In the present configuration, manual handling is required only for transferring the sample from the load-lock to the optical measurement stage; automating this step would enable fully autonomous operation for up to five consecutive runs per load-lock loading.

### Automated optical analysis

Optical bandgap energy (*E*_g_) and *E*_U_ were automatically extracted from the measured optical transmittance spectra ${{{\rm{Tr}}}}\left(E\right)$ using a custom analysis script. The extracted *E*_U_ was then forwarded to an automated dataset update routine used for subsequent Bayesian optimization (BO). The absorption coefficient $\alpha \left(E\right)$ was estimated from the Beer–Lambert relation^78^. In the simplest approximation (neglecting reflectance and interference fringes),

4 $$\alpha \left(E\right)=-\frac{1}{d}{{\mathrm{ln}}}\left[{{{\rm{Tr}}}}\left(E\right)+\varepsilon \right]$$

where *d* is the film thickness and *ε* is a small constant to avoid singularities at ${{{\rm{Tr}}}}\to 0$. *E*_g_ was then obtained from a Tauc plot assuming a direct bandgap structure^79^,

5 $${\left(\alpha {{{\rm{h}}}}\nu \right)}^{2}\propto {{{\rm{h}}}}\nu -{E}_{{{{\rm{g}}}}},$$

by linear fitting in the appropriate region and taking the intercept with the ${{{\rm{h}}}}\nu$ axis (see Supplementary Fig. 7a). Although *β*-Ga_2_O_3_ is commonly regarded as an indirect-bandgap semiconductor, first-principles calculations show that the minimum direct bandgap is only tens of meV above the indirect fundamental bandgap^80^, which explains the strong near-edge absorption. Consistent with this, Ricci et al. pointed out that the near-edge optical behavior is effectively that of a Γ direct-like^81^, and direct-allowed Tauc analyses are therefore widely used in practice for *β*-Ga_2_O_3_ thin films^42^. *E*_U_ was extracted from the exponential absorption Urbach tail^43^ just below the estimated ${E}_{{{{\rm{g}}}}}$ by fitting

6 $${ln}\left[\alpha \left(E\right)\right]=\frac{1}{{E}_{{{{\rm{U}}}}}}E+C,$$

where C is a constant (see Supplementary Fig. 7b). Details of the automated fitting procedure (including window selection criteria and parameter settings) are available via the Code availability section.

The extracted *E*_U_ is expected to be only weakly sensitive to the film thickness, since a constant error in the assumed film thickness only produces a vertical offset in ln[$\alpha \left(E\right)$], while leaving the fitted slope, and hence *E*_U_, unchanged in principle. To experimentally verify that the *E*_U_ values used in this study are not dominated by thickness variation, we additionally grew a thinner film under a representative low-*E*_U_ condition. For the condition (520 °C, 97 W, 26.5 cm^3^ STP min^−1^, 0.4 cm^3^ STP min^−1^), the nominal 55-nm film yielded *E*_U_ = 216 meV. When the deposition time was intentionally reduced to obtain an ≈ 10% thinner film with a nominal thickness of 50 nm, the extracted *E*_U_ was 220 meV, which is close to the value obtained for the nominal 55-nm film. This result supports that a thickness difference of this magnitude does not significantly alter the extracted *E*_U_.

To prevent noisy or physically invalid spectra from biasing the optimization, the automated fitting procedure used predefined quality criteria. Both the Tauc fit for *E*_g_ and the Urbach-tail fit for *E*_U_ were required to satisfy *R*^2^ ≥ 0.995. In addition, spectra that did not correspond to successful *β*-Ga_2_O_3_ film growth, identified by a transmittance below 0.8 at 500 nm, were treated as failed/not a number (NaN) evaluations rather than valid objective values. These NaN evaluations were handled in the BO workflow by worst-value imputation, as described below.

### Bayesian optimization with failures and adaptive prior mean

The BO objective was to minimize the *E*_U_. To address practical stagnation in self-driving sputtering, namely, missing/NaN evaluations caused by growth failures under off-optimum conditions and trapping in locally optimal regions, we developed a Gaussian-process (GP)-based BO loop featuring two key innovations: NaN handling by worst-value imputation^44^ and adaptive modification of the GP prior mean^45^. After each growth, the measured transmittance spectrum was analyzed to obtain *E*_U_, and the resulting value was fed back to a BO algorithm to determine the next set of growth parameters. The parameter vector was defined as ${{{\bf{x}}}}=({T}{\space}({\scriptstyle{{\circ}}\atop} \! {{{\rm{C}}}}),{P}_{{{{\rm{RF}}}}}{\space}({{{\rm{W}}}}),{F}_{{{{\rm{Ar}}}}}{\space}({{{{\rm{cm}}}}}^{3}{{\space}{{\rm{STP}}{\space}}}{\min }^{-1}),{F}_{{{{{\rm{O}}}}}_{2}}{\space}({{{{\rm{cm}}}}}^{3}{{\space}{{\rm{STP}}{\space}}}{\min }^{-1}))$, with bounds $T{\space}\epsilon{\space}[{{\mathrm{30,700}}}],{P}_{{{{\rm{RF}}}}}{\space}\epsilon{\space} [{{\mathrm{50,150}}}],{F}_{{{{\rm{Ar}}}}}{\space}\epsilon{\space}[{{\mathrm{10,50}}}],{{{\rm{and\,}}}}{\space}{F}_{{{{{\rm{O}}}}}_{2}}{\space}\epsilon{\space}[{{\mathrm{0,10}}}].$ At iteration *n*, a GP surrogate model was fitted to the dataset ${{D}_{n-1}}=\left\{\left({{{{\bf{x}}}}}_{i},{{{{\bf{y}}}}}_{i}\right)\right\}_{i=1}^{n-1}$, where ${y}_{i}$ denotes the observed *E*_U_, i.e., ${y}_{i}={E}_{{{{\rm{U}}}}}({{{{\bf{x}}}}}_{{{{\bf{i}}}}})$, obtained from the previous iterations of the self-driving loop. Although BO is commonly formulated as a maximization problem, minimization was handled by negating the observations when evaluating the acquisition function.

To improve robustness in closed-loop experiments, we implemented the two mechanisms described above. First, when a sputtering growth failed and *E*_U_ was unavailable (NaN) due to growth parameters that are far from optimal, the trial was not discarded; instead, we imputed the objective by assigning the worst (largest) experimental *E*_U_ value at that time^44^:

7 $${\widetilde{y}}_{{n}^{{\prime} }}=\left\{\begin{array}{cc}{y}_{n} & {{{\rm{if}}}}\,{y}_{n}\, \ne \, {{{\rm{NaN}}}},\\ {\max }_{1\le i < n}{\widetilde{y}}_{i} & {{{\rm{if}}}}\,{y} \,_{n} \,={{{\rm{NaN}}}}.\end{array}\right.$$

This imputation of the dataset ${{D}_{n}=\left\{\left({{{{\bf{x}}}}}_{i},{{{{\bf{y}}}}}_{i}\right)\right\}}_{i=1}^{n}$ by ${{\widetilde{D}}_{n}=\left\{\left({{{{\bf{x}}}}}_{i},{\widetilde{{{{\bf{y}}}}}}_{i}\right)\right\}}_{i=1}^{n}$ enables direct exploration of a wide parameter space while avoiding experimental failure. Second, we used an adaptive prior mean strategy for the GP mean function^45^. In the GP, the predictive mean depends on the prior mean function $\eta ({{{\bf{x}}}})$ (here taken as a constant $\eta ({{{\bf{x}}}})={m}_{0}$). The prior mean hyperparameter was resampled at each iteration from a uniform distribution bounded by the minimum and maximum of the observed values:

8 $${m}_{0}^{n}={{{\mathcal{U}}}}\left({\min }_{1\le i < n}{\widetilde{y}}_{i},{\max }_{1\le i < n}{\widetilde{y}}_{i}\right)$$

which helps the search escape locally optimal regions and promotes exploration of previously unseen regions.

The next growth condition ${{{{\bf{x}}}}}_{{n}^{{\prime} }+1}$ was chosen by maximizing the expected improvement (EI) acquisition function computed from the GP predictive distribution. As a practical GP configuration, we adopted a Matérn 5/2 kernel (commonly used for functions with relatively steep gradients)^82^ and included a constant observation-noise variance term to account for run-to-run variability.

### Explainable surrogate decomposition using random forests

Random forest regression was implemented using scikit-learn’s RandomForestRegressor^83^ and trained on *N* = 66 samples acquired by the self-driving sputtering. Hyperparameters were tuned using 5-fold cross-validation on the BO-acquired dataset. The final model used for interpretability employed 300 trees with unlimited depth, a minimum of one sample per leaf, and the square root of the number of available features considered at each split. This model achieved an in-sample *R*^2^ = 0.92, while a 5-fold cross-validation yielded a more conservative *R*^2^ ≈ 0.43, reflecting the limited number and BO-biased distribution of samples in parameter space. Accordingly, for the original *N* = 66 BO-acquired dataset, the random forest is used in this work primarily as an interpretable surrogate to summarize the *E*_U_ landscape within the explored growth window, rather than as a high-accuracy predictive model for arbitrary unseen conditions.

Let $\hat{f}\left(T,{P}_{{{{\rm{RF}}}}},{F}_{{{{\rm{Ar}}}}},{F}_{{{{{\rm{O}}}}}_{2}}\right)$ denote the trained random forest regression function. Feature importance and partial dependence analyses were performed on $\hat{f}$. Feature importances were obtained from the impurity-based importance scores, where the decrease in mean squared error at each split is accumulated for the corresponding parameter and averaged over all trees, followed by normalization so that the importances of all features sum to one^70,71^.

1D partial dependence functions ${g}_{s}\left({z}_{s}\right)$ were defined for each input parameter $s\in \{T,{P}_{{{{\rm{RF}}}}},{F}_{{{{\rm{Ar}}}}},{F}_{{{{{\rm{O}}}}}_{2}}\}$ and parameter value ${z}_{s}$ as

9 $${g}_{s}\left({z}_{s}\right)=\frac{1}{N}{\sum}_{i=1}^{N}\hat{f}({z}_{s},{x}_{i,-s})$$

where ${x}_{i,-s}$ denotes all parameter values of sample *i* (from 1 to *N*) except ${x}_{i,s}$. Here, ${x}_{i,s}$ is the value of parameter *s* in sample *i*. Similarly, for a pair of parameters (*s*, *t*) and parameter values ${z}_{s}$ and ${z}_{t}$, the 2D partial dependence function was defined as

10 $${g}_{s,t}\left({z}_{s},{z}_{t}\right)=\frac{1}{N}{\sum}_{i=1}^{N}\hat{f}({z}_{s},{z}_{t}{,x}_{i,-s,-t})$$

where $t\in \{T,{P}_{{{{\rm{RF}}}}},{F}_{{{{\rm{Ar}}}}},{F}_{{{{{\rm{O}}}}}_{2}}\}$ and ${x}_{i,-s,-t}$ denotes all parameter values of sample *i* except ${x}_{i,s}$ and ${x}_{i,t}$. Following Eq. (9), each 1D PDP was evaluated on a grid spanning the observed range of *s*. 2D PDPs were also calculated in the same manner for selected pairs of parameters, such as (*T*, ${P}_{{{{\rm{RF}}}}}$).

Pairwise interaction strength between input parameters was quantified using Friedman’s *H*^2^ interaction statistic^75^ based on the above partial dependence functions. For a given parameter pair (*s*, *t*), the interaction component was defined as

11 $${g}_{s,t}^{{{\mathrm{int}}}}({z}_{s},{z}_{t})\,=\,{g}_{s,t}\left({z}_{s},{z}_{t}\right)-{g}_{s}\left({z}_{s}\right)-{g}_{t}\left({z}_{t}\right)+{c}_{s,t}$$

where ${c}_{s,t}$ is the constant chosen such that $\left\langle {g}_{s,t}^{{{\mathrm{int}}}}({z}_{s},{z}_{t})\right\rangle=0$ over the 2D PDP grid. Friedman’s *H*^2^ statistic for the parameter pair (*s*, *t*) was then calculated as

12 $${H}_{s,t}^{2}=\frac{\sum {{g}_{s,t}^{{{\mathrm{int}}}}({z}_{s},{z}_{t})}^{2}}{\sum {({g}_{s,t}\left({z}_{s},{z}_{t}\right)-{c}_{s,t})}^{2}}$$

where the sums over (${z}_{s},{z}_{t}$) approximate the corresponding integrals using the same parameter grid as that employed for computing the 2D PDPs.

### Reporting summary

Further information on research design is available in the Nature Portfolio Reporting Summary linked to this article.

## Supplementary information

Supplementary Information

Description of Additional Supplementary Files

Supplementary Data

Supplementary Movie

Reporting Summary

Transparent Peer Review file

## Source data

Source data

## Data Availability

The data that support the findings of this study are available as Supplementary Data 1 (on GitHub^84^) and from the corresponding authors upon request. Source data are provided with this paper.

## Code availability

The code used in this study is available as Supplementary Data 1 (on GitHub^84^) and from the corresponding authors upon request.
