# Supplementary material for: Interpretable self-driving sputtering epitaxy reveals human-usable growth rules for β-Ga2O3 films
Source: Nat Commun. 2026 Aug 13;17:9714. doi: 10.1038/s41467-026-76533-0 (PMC13562525; doi:10.1038/s41467-026-76533-0)
Supplement: Supplementary file 1 [file 41467_2026_76533_MOESM1_ESM.pdf]

Supplementary Information

Interpretable self-driving sputtering epitaxy reveals human-usable growth rules for β-Ga_2O_3 films

Yuki K. Wakabayashi,[1,*] Yui Ogawa,[1,*] Franz Benedict Romero,[1] Coleman Wagner,[1] Takuma Otsuka,[2] and Yoshitaka Taniyasu[1]

[1]*Basic Research Laboratories, NTT, Inc., Atsugi, Kanagawa 243-0198, Japan*

[2]*Communication Science Laboratories, NTT, Inc., Soraku-gun, Kyoto 619-0237, Japan*

[*]Correspondence: yuuki.wakabayashi@ntt.com, yui.ogawa@ntt.com

Supplementary Note 1. Automated sputtering station.

Supplementary Fig. 1 shows a photograph of the automated sputtering station. A fully automated sequence, from wafer transfer from the load lock to the sputtering chamber, sputter growth, and subsequent transfer back to the load lock, typically completes within ≈2 h per run (Supplementary Video). The load lock can store up to five wafers, enabling up to ~10 h of continuous unattended operation for five consecutive growth runs. After this batch, an operator can replenish wafers in the load lock in ≈20 min. With this workflow, the system can achieve a throughput of ≈12 sputtered wafers per 24 h. All process steps during each run (transfer, growth, and return) are executed automatically under the control of the scheduling software. In the present study, wafers were manually transferred from the load lock to a separate optical setup between runs; the optical measurement and analysis were automated, and the resulting metrics were used to automatically select the next growth conditions and automatically trigger the subsequent growth run without further user input (see the main text).

The practical closed-loop throughput in the present study was ≈ 7–8 completed growth/evaluation cycles per working day because each sputter growth run was followed by manual transfer from the load-lock to the ex situ optical measurement setup. If this remaining transfer step is automated using robotic handlers connecting the load-lock and optical measurement setup, the closed-loop workflow could approach the intrinsic sputtering-station throughput of ≈12 wafers per 24 h.

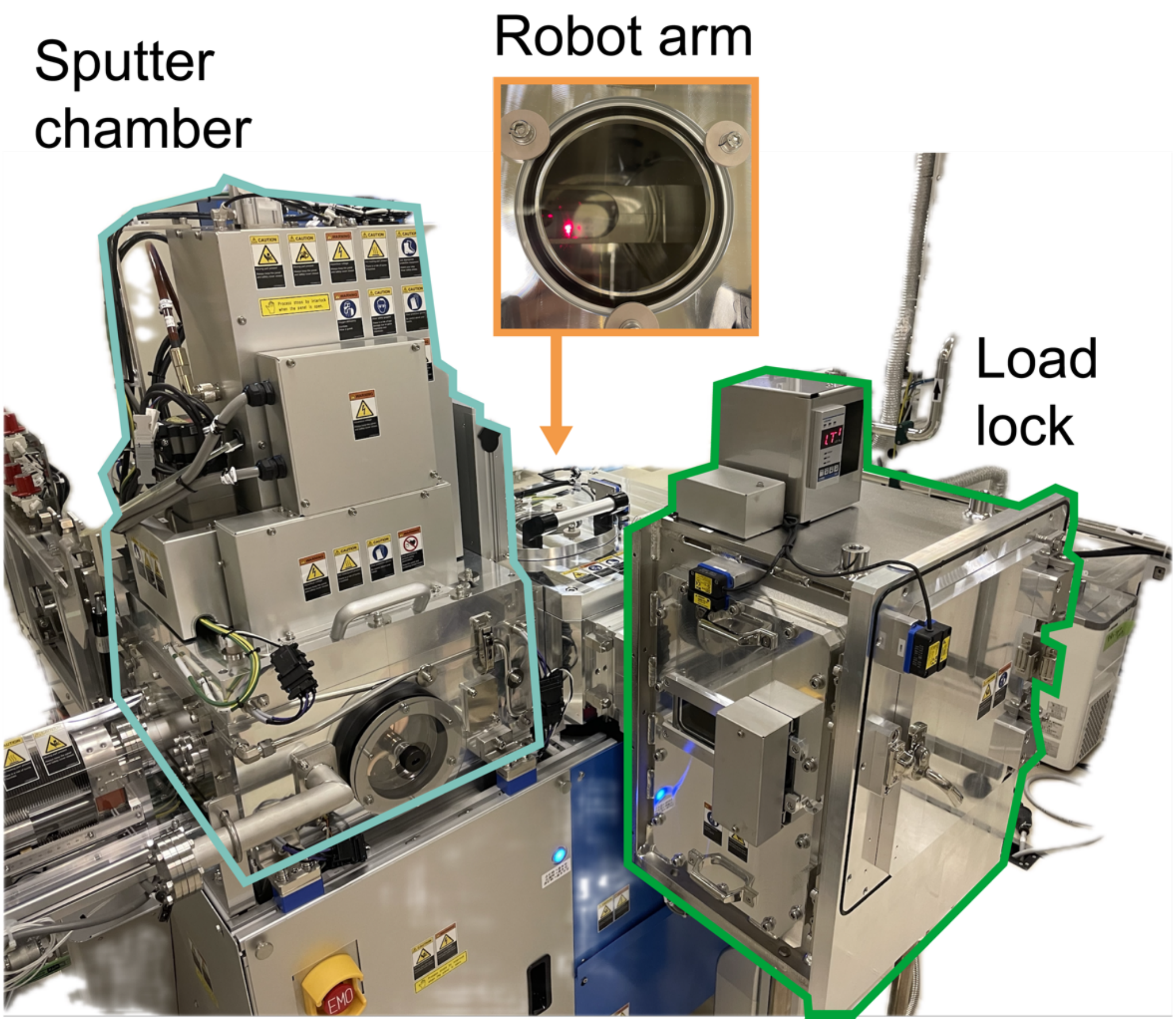

Supplementary Fig. 1. Photograph of the automated sputtering station. Photograph of the sputter chamber, the robot arm for sample handling in vacuum, and the load-lock that stocks up to five wafers.

Supplementary Note 2. Pseudocode-style summary of the closed-loop Bayesian-optimization (BO) procedure.

The BO procedure used to select sputter-growth parameters in the self-driving sputtering system is summarized in Supplementary Box 1. The optimization objective was to minimize the Urbach energy E_U. The four-dimensional input vector was defined as $\mathbf{x} = (T,\ P_{\mathrm{RF}},\ F_{\mathrm{Ar}},\ F_{\mathrm{O_2}})$, where T is the substrate temperature, P_{RF} is the RF power, and F_{Ar} and $F_{\mathrm{O_2}}$ are the Ar and O_2 flow rates, respectively. The first seven runs were used as seed experiments by sweeping T while fixing P_{RF}, F_{Ar}, and $F_{\mathrm{O_2}}$ at a pragmatic baseline condition. After these seed experiments, all subsequent conditions were selected as four-dimensional recipes by the BO loop. This procedure clarifies that, from run 8 onward, the BO loop selected T, P_{RF}, F_{Ar}, and $F_{\mathrm{O_2}}$ simultaneously as a four-dimensional recipe. The random-forest surrogate used for partial-dependence and interaction analyses was not part of this self-driving decision-making loop; it was trained after the BO campaign to interpret the accumulated dataset and extract human-executable growth rules.

Supplementary Box 1. Bayesian-optimization procedure used in the self-driving sputtering system.

Input: initial dataset $D_n = \{(\mathbf{x}_i, E_{\mathrm{U,i}})\}_{i=1}^{n=N_{\mathrm{init}}}$, total experimental budget N

Output: best parameter $\mathbf{x}_{i^*}$ such that $i^* = \arg\min_{1\le i\le N} E_{\mathrm{U},i}$

$n \leftarrow N_{\mathrm{init}}$
while $n < N$ **do**
Convert data D_n into $\widetilde{D}_n = \{(\mathbf{x}_i, \tilde{\mathbf{y}}_i)\}_{i=1}^{n}$ by applying worst-value imputation by Eq. (7)
fit Gaussian process using $\widetilde{D}$
calculate the expected improvement $\mathrm{EI}(\tilde{\mathbf{x}})$ over the normalized 4D parameter space $\tilde{\mathbf{x}}$
$n \leftarrow n + 1$
find next parameter $\tilde{\mathbf{x}}_n = \arg\max_{\tilde{\mathbf{x}}} \mathrm{EI}(\tilde{\mathbf{x}})$
rescale to original parameter $\mathbf{x}_n$ from $\tilde{\mathbf{x}}_n$
send $\mathbf{x}_n$ to the sputtering-control workflow
grow the film under $\mathbf{x}_n$
measure the optical transmittance spectrum
extract the Urbach energy $E_{\mathrm{U},n}$ from the measured spectrum
add the new result to raw dataset $D_n \leftarrow D_{n-1} \cup \{(\mathbf{x}_n, E_{\mathrm{U},n})\}$
end while

Supplementary Note 3. Deposition-rate calibration for nominal thickness control

Supplementary Table 1 summarizes the deposition rates estimated from stylus profilometry measurements of films grown under various conditions. Supplementary Figure 2 shows the deposition rate as a function of P_{RF}.

P_{RF} (W)	F_{Ar} (cm^3 STP min^{-1})	F_{O_2} (cm^3 STP min^{-1})	Rate (nm min^{-1})
60	9	0	0.64
80	9	0	0.72
100	6	0	0.84
100	9	0	0.89
100	12	0	0.86
100	30	0	0.83
100	30	1	0.80
100	30	5	0.81
119	26	1	1.18
130	30	0	1.42

Supplementary Table 1. Pre-calibrated deposition-rate data used for nominal thickness control. The table summarizes the deposition rates estimated from film thicknesses measured by a stylus profiler after deposition under representative P_{RF}, F_{Ar}, and F_{O2} conditions.

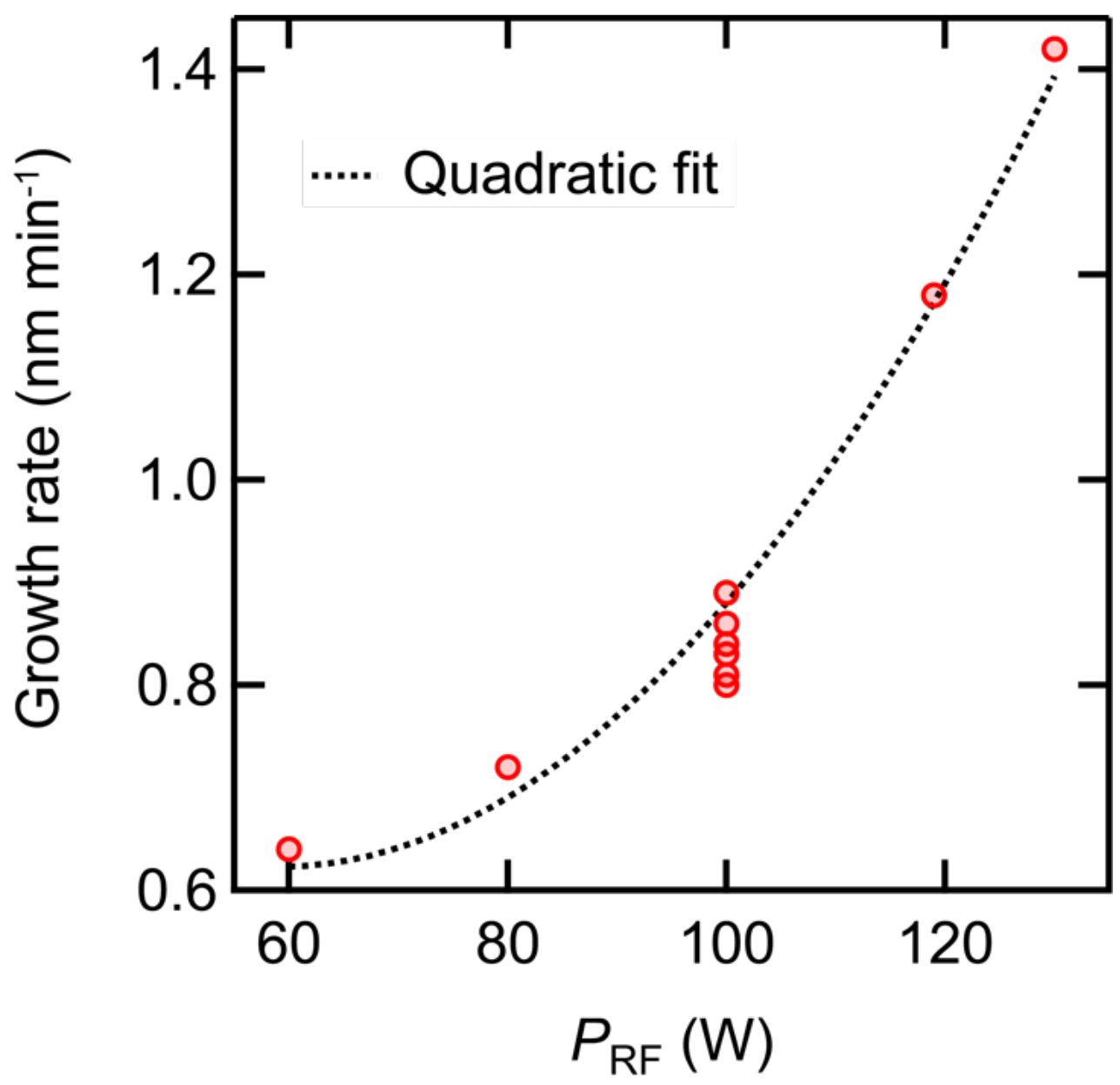

Supplementary Fig. 2. Deposition-rate calibration used for nominal thickness control. Deposition rate as a function of P_{RF}, using the data in Supplementary Table 1. The solid curve is the empirical quadratic fit.

Supplementary Note 4. Replicate growth experiments for evaluating reproducibility

To evaluate the reproducibility of the present study, we carried out replicate experiments under several representative conditions yielding different E_U values. These experiments show that the measured E_U values are reproducible across low- and high-E_U growth regimes (Supplementary Table 2). For example, the BO-identified optimum condition yielded E_U = 182 and 184 meV in two independent runs, representative nearby low-E_U conditions gave E_U values in the range of 197–219 meV, with a combined mean of 205 ± 9 meV across several nearby conditions, and an off-optimal condition reproducibly yielded much larger E_U values of 338 and 376 meV, confirming that the observed differences in E_U reflect systematic growth-condition dependence rather than incidental run-to-run scatter.

Growth condition	T (°C)	P_{RF} (W)	F_{Ar} (cm^3 STP min^{-1})	F_{O_2} (cm^3 STP min^{-1})	E_U (meV)
BO-identified optimum	507	119	26	1	182, 184
Nearby low-E_U condition A	510	97	26.5	0.6	203, 199
Nearby low-E_U condition B	520	97	26.5	0.4	216, 219, 197
Nearby low-E_U condition C	512	105	26.5	0.7	198, 203
Off-optimal condition	512	100	30	5	338, 376

Supplementary Table 2. Replicate growth runs used to evaluate the reproducibility of E_U across representative growth regimes. The table includes repeated runs for the BO-identified optimum, several nearby low-E_U conditions, and a clearly off-optimal condition. The nearby low-E_U conditions A-C represent multiple distinct conditions close to the optimum.

Supplementary Note 5. Multi-domain in-plane epitaxy of heteroepitaxial β-Ga_2O_3 on C-plane Al_2O_3 (0001).

Systematic cross-sectional high-angle annular dark-field scanning transmission electron microscopy (HAADF-STEM) measurements confirm that the Bayesian optimization (BO)-optimized β-Ga_2O_3 film grown on C-plane Al_2O_3 (0001) consists of multiple equivalent in-plane rotational domains while maintaining a common out-of-plane orientation. This multi-domain in-plane epitaxial structure is consistent with the in-plane X-ray diffraction (XRD) ϕ-scan discussed in the main text, where the sixfold peak set corresponds to three equivalent in-plane domains of the ($\bar{2}01$)-oriented β-Ga_2O_3. Supplementary Fig. 3 summarizes the epitaxial orientation relationship using schematic atomic models. The shaded planes indicate the interface/out-of-plane plane of the film, and representative zone-axis views of the β-Ga_2O_3 lattice are provided for reference. This schematic representation is intended to clarify the in-plane registry that gives rise to multiple rotational variants on the hexagonal C-plane Al_2O_3 surface.

Supplementary Fig. 4 shows cross-sectional HAADF-STEM images acquired along the [$11\bar{2}0$] direction of the C-plane Al_2O_3 substrate. As illustrated in Supplementary Fig. 4d, the projected atomic arrangements of the rotational domains appear very similar in this viewing direction. Therefore, atomic-resolution images do not provide a straightforward contrast signature to unambiguously distinguish adjacent in-plane rotated domains (Supplementary Fig. 4b,c). In contrast, when viewed along the [$1\bar{1}00$] direction of the C-plane Al_2O_3 substrate (Supplementary Fig. 5), the projected β-Ga_2O_3 lattice differs markedly between rotational domains, enabling clear identification of the in-plane rotational variants. Taken together, the observations from these two orthogonal viewing directions confirm the multi-domain in-plane epitaxial structure summarized in Supplementary Fig. 3.

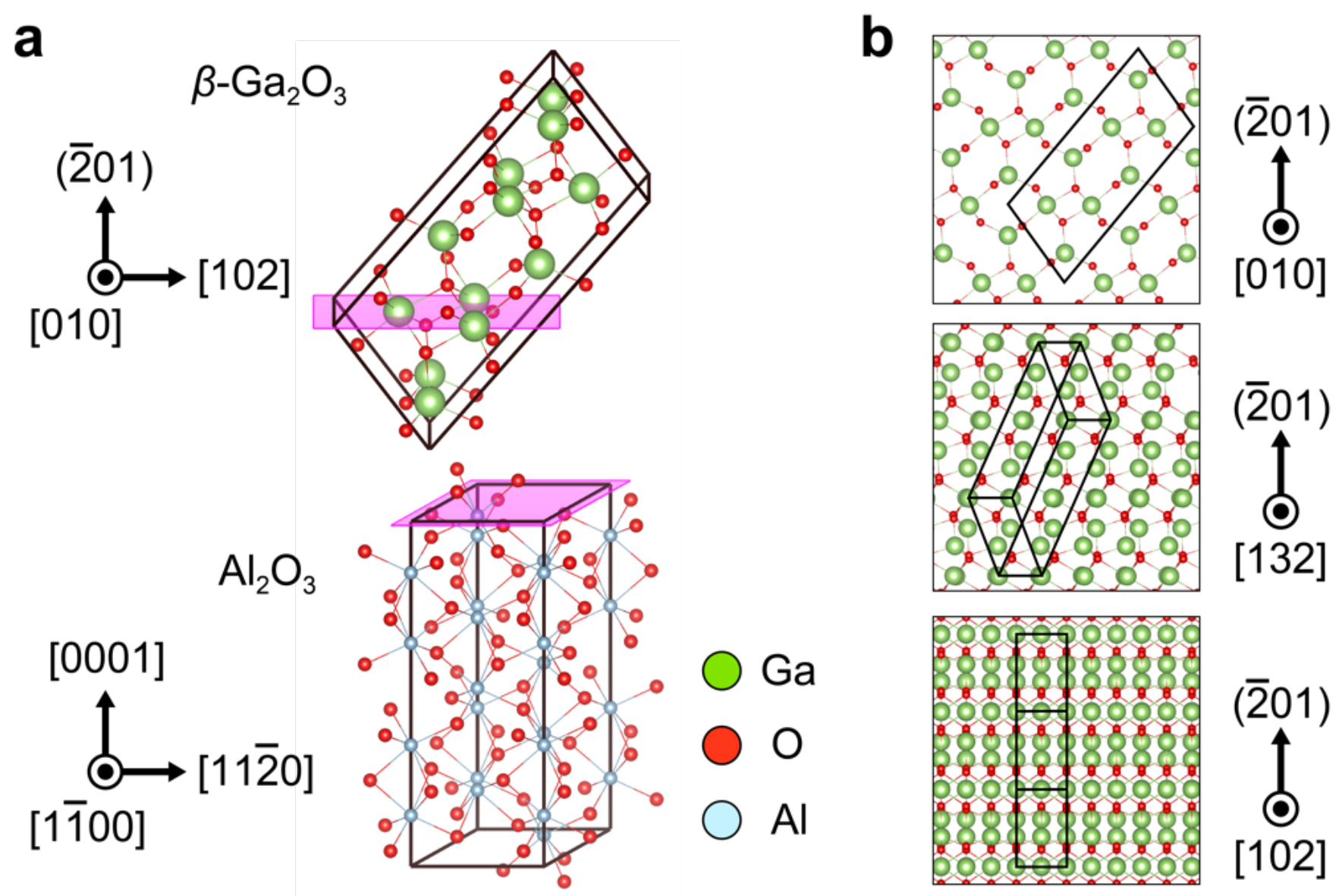

Supplementary Fig. 3. Crystallographic relationship for β-Ga_2O_3 heteroepitaxy on C-plane Al_2O_3. a Schematic atomic models illustrating the epitaxial orientation relationship. The shaded planes indicate the corresponding interface/out-of-plane plane of the film. **b** Schematic atomic models of the β-Ga_2O_3 lattice viewed along representative zone axes (as labeled).

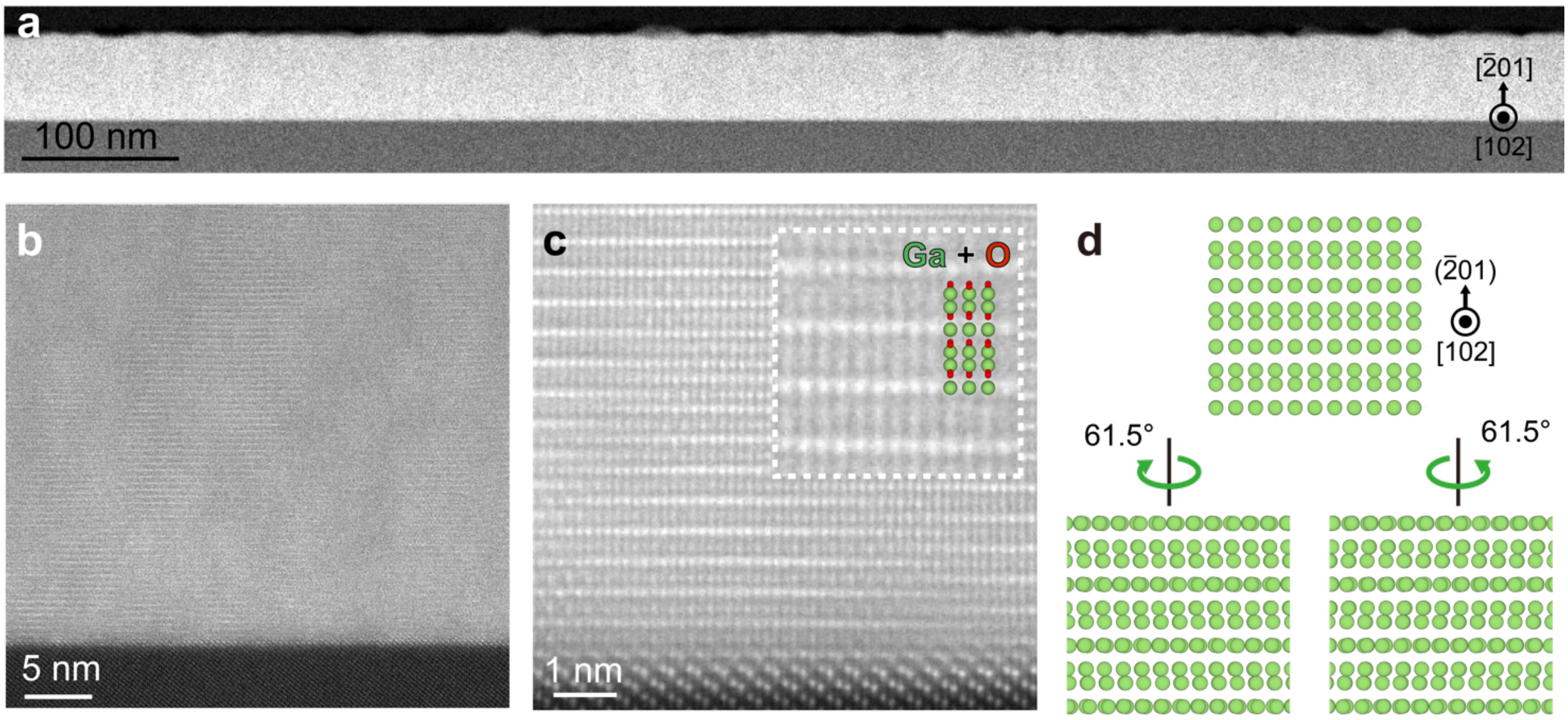

Supplementary Fig. 4. Heteroepitaxial *β*-Ga_2O_3 on Al_2O_3 grown under the optimized condition. **a** HAADF-STEM image of the heteroepitaxial *β*-Ga_2O_3 film viewed along the [11$\bar{2}$0] direction of the C-plane Al_2O_3 substrate. **b** Magnified image near the interface in **a**. **c** Magnified image near the interface in **b**. The inset in **c** represents an atomic-resolution magnified image with Ga-occupied (green spheres) and O-occupied (red spheres) columns. **d** Schematic illustration of rotational domains in the *β*-Ga_2O_3 lattice (Ga sublattice shown).

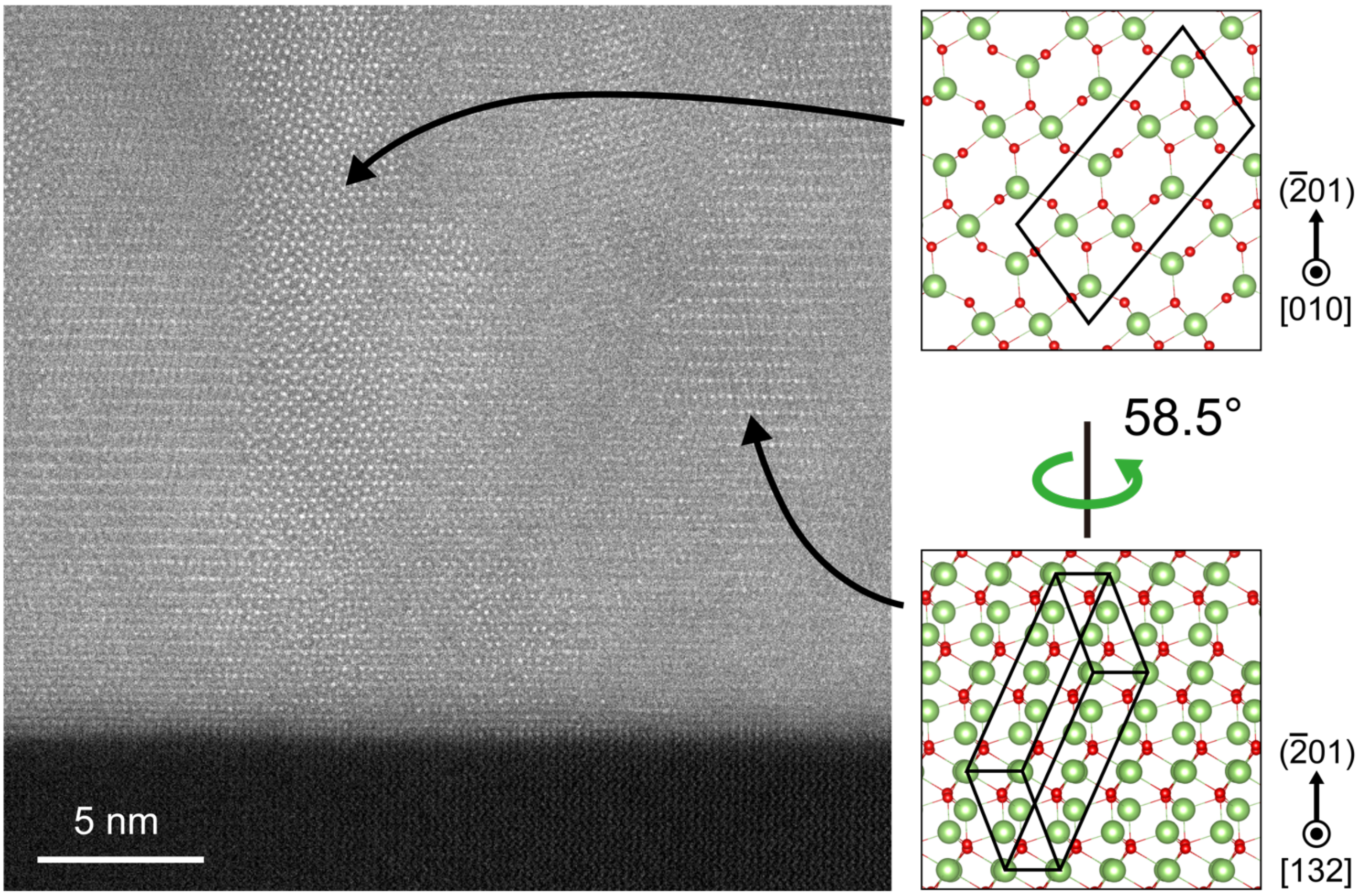

Supplementary Fig. 5. Atomic-scale view of in-plane rotational domains. Atomic-resolution HAADF-STEM image, showing rotational domains in the heteroepitaxial β-Ga_2O_3 film viewed along the [1$\bar{1}$00] direction of the C-plane Al_2O_3 substrate. The right panels show the schematic illustrations of rotational domains, depicting the two viewing directions and the in-plane rotation angle (58.5°) between domains while maintaining the same out-of-plane orientation, in the β-Ga_2O_3 lattice with Ga-occupied (green spheres) and O-occupied (red spheres) columns.

Supplementary Note 6. Atomic force microscopy (AFM) surface morphology of the homoepitaxial β-Ga_2O_3 film.

Supplementary Fig. 6 shows the AFM surface morphology of the homoepitaxial β-Ga_2O_3 film grown under the BO-identified optimized condition. The surface exhibits a smooth, laterally uniform morphology with no observable pits. The root-mean-square roughness is 1.10-1.25 nm for 500 × 500 nm^2 , 1 × 1 μm^2 and 3 × 3 μm^2 scans, indicating that the measured roughness is not strongly dependent on the scan size over the micrometer length scale. This smooth surface morphology is consistent with the laterally uniform, single-crystal homoepitaxial film observed by cross-sectional HAADF-STEM in Fig. 4 in the main text.

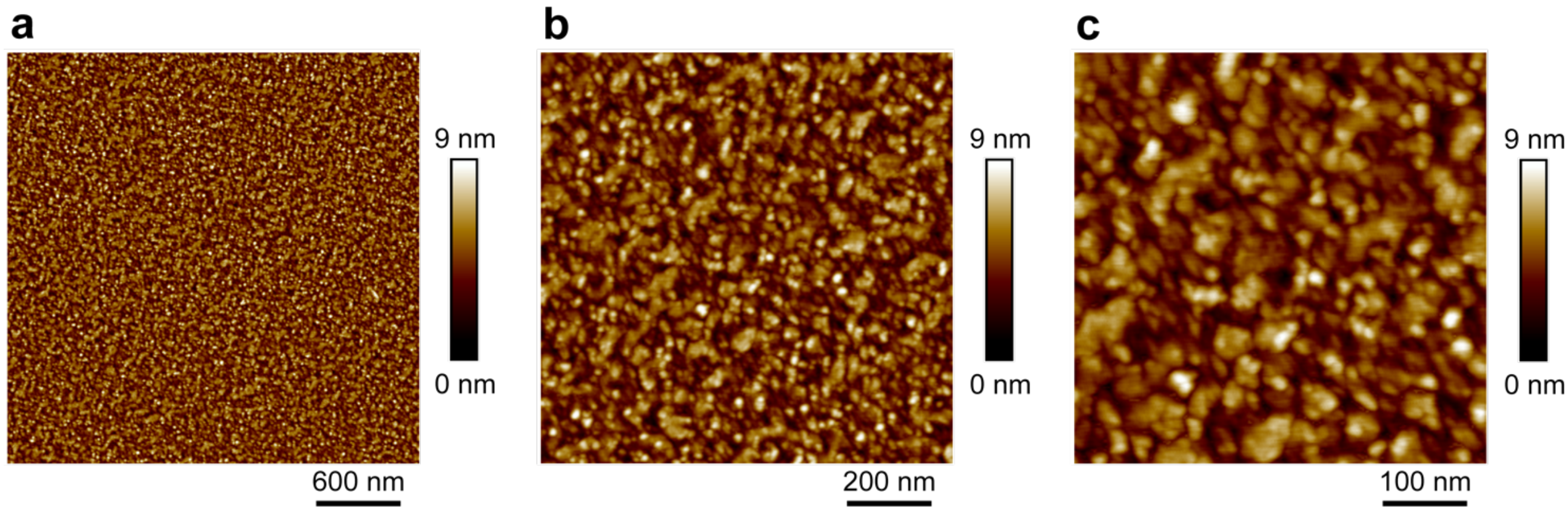

Supplementary Fig. 6. AFM images of the homoepitaxial β-Ga_2O_3 film. a 3 µm × 3 µm, **b** 1 µm ×1 µm, and **c** 500 nm × 500 nm AFM images for the homoepitaxial β-Ga_2O_3 film grown under the BO-identified optimized condition.

Supplementary Note 7. Gaussian-process (GP) surrogate analysis for interpretable growth-rule distillation.

To verify that the near-additive E_U landscape with a modest T-F_{O_2} interaction structure obtained from the random forest surrogate is not specific to random forest, we re-fitted a GP surrogate to the same dataset (66 samples) acquired by the self-driving sputtering and repeated the same post hoc interpretability analyses (one-dimensional (1D) and two-dimensional (2D) partial dependence plots (PDPs) with interpretable decomposition and Friedman's H^2-statistic analysis). All GP settings were identical to those used in the BO loop (kernel form, noise model, and training settings) (see Methods section "Bayesian optimization with experimental failures and adaptive GP prior mean"), with one exception: we set the GP prior mean to a constant function equal to the empirical mean of the observed data so that predictions in unobserved regions revert to a neutral value, avoiding overly optimistic or pessimistic extrapolation. A representative optimized GP fit yielded Matérn length scales (in normalized x space) of [0.236, 0.665, 10.0, 0.149] for (T, P_{RF}, F_{Ar}, F_{O_2}), with an effective observation-noise level of ~0.025.

This GP model achieves an in-sample R^2 = 0.76. Although this indicates a reasonable fit to the BO dataset, it is lower than the random forest surrogate (R^2 = 0.92). The GP surrogate also yielded a lower 5-fold cross-validated performance (R^2 = 0.20) than the random-forest surrogate trained on the same 66-run dataset (R^2 = 0.43), indicating that the GP did not provide a stronger predictive basis for post hoc response-surface decomposition in the original BO-acquired dataset. These results support our choice of the random forest surrogate for extracting interpretable growth rules, providing both higher predictive fidelity on the closed-loop dataset and a mature interpretability toolbox. From the GP surrogate over the dataset, we evaluated: an additive approximation constructed from the 1D PDPs of each parameter, and an additive + (T-F_{O_2}) interaction approximation constructed by adding the (T-F_{O_2}) interaction component extracted from the 2D PDP (same procedures as in the main text). The additive-only model was insufficient to reproduce the GP predictions (R^2 = 0.51), whereas including a single (T-F_{O_2}) interaction term markedly improved the approximation (R^2 = 0.75). To quantify interaction strength, we also computed the Friedman H^2 interaction index from PDPs. The dominant interaction was obtained for the T-F_{O_2} pair (H^2 = 0.334), while other pairwise interactions were substantially smaller (≤ 0.046). These results corroborate the qualitative conclusion from the random forest surrogate: within the BO-explored growth window, the E_U landscape is largely near-additive, with only a physically plausible coupling primarily between temperature and O_2 flow.

Supplementary Note 8. Representative optical extraction of the band gap energy (Eg) and Urbach energy (E_U).

Supplementary Fig. 7 shows a representative analysis procedure used to extract Eg and E_U from the measured transmittance spectra of a β-Ga_2O_3 film (E_g = 5.00 eV and E_U = 227 meV) grown on C-plane Al_2O_3. Eg is estimated from a linear fit in the appropriate region of the Tauc plot (Supplementary Fig. 7a). Supplementary Fig. 7b shows the corresponding Urbach analysis, where E_U is obtained from a linear fit to $\ln[\alpha(E)]$ in the exponential absorption-tail region, where $\alpha(E)$ is the absorption coefficient. The formalization of the procedure is described in the main text (Methods section "Automated optical analysis") and was applied uniformly to all samples in the closed-loop experiments.

The influence of thin-film interference fringes on the extracted E_U is expected to be negligible under the present experimental conditions. For a nominal film thickness of 55 nm and a representative refractive index of $n \approx 1.9$–2.0[1,2], the optical thickness is $2nd \approx 210$–220 nm, corresponding to less than one interference oscillation over the measured wavelength range of 190–600 nm. Within the narrower Urbach-tail fitting window just below E_g, such interference would therefore appear only as a slowly varying background rather than as pronounced fringes. The representative $\ln[\alpha(E)]$ spectrum in Supplementary Fig. 7b exhibits an approximately linear Urbach-tail region, supporting that thin-film interference does not significantly affect the extracted E_U.

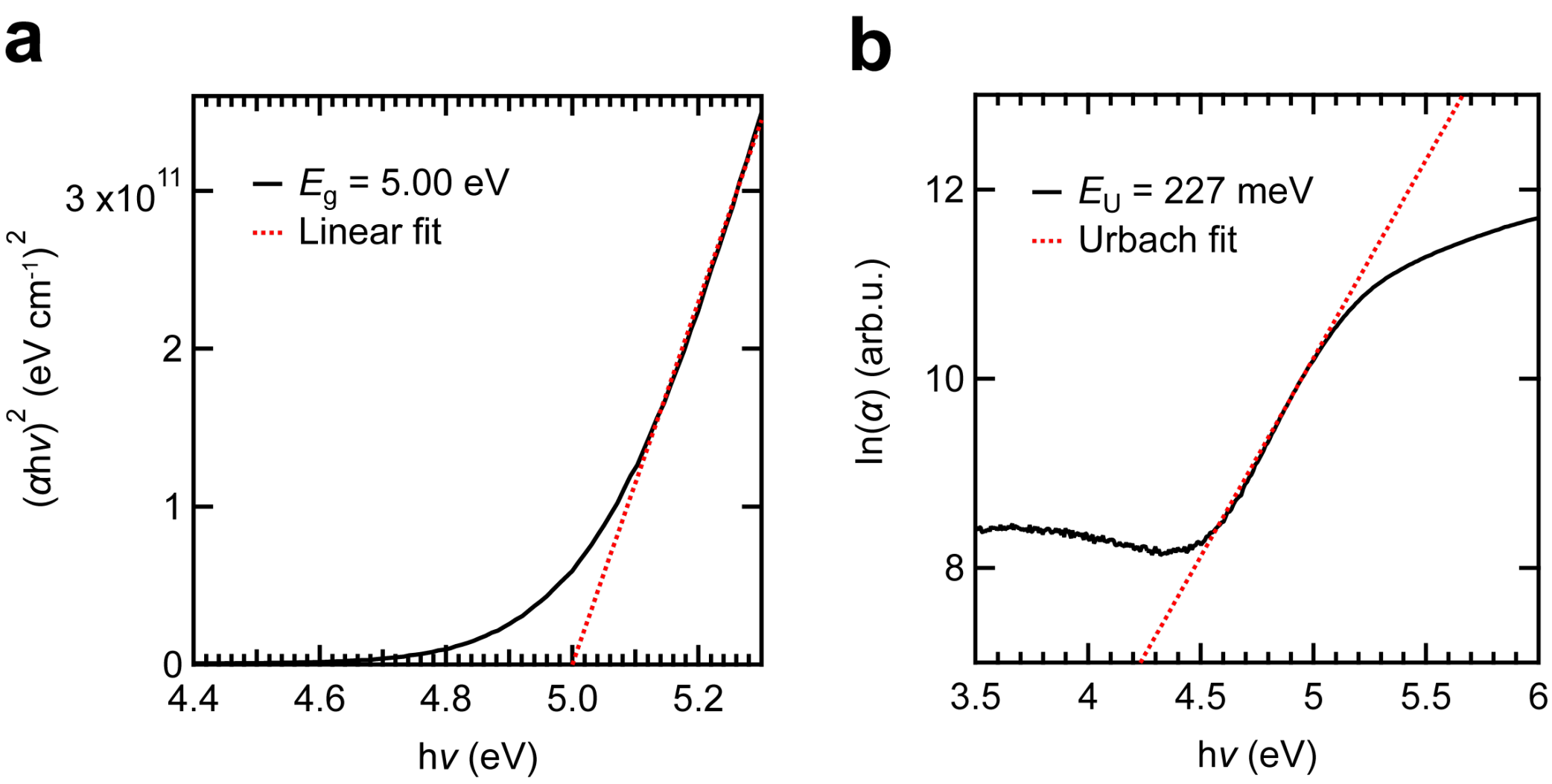

Supplementary Fig. 7. Representative procedure for optical extraction of E_g and E_U of the β-Ga_2O_3 film with E_g = 5.00 eV and E_U = 227 meV on C-plane Al_2O_3. a Tauc plot $(\alpha h\nu)^2$ as a function of photon energy $h\nu$, with a linear fit used to estimate E_g. **b** Urbach energy analysis shown as $\ln(\alpha)$ versus $h\nu$ with a linear fit in the exponential tail region used to extract E_U.

Supplementary Note 9. Expanded literature benchmark.

Supplementary Table 3 provides an expanded literature survey of the optical, structural, and surface quality of β-Ga_2O_3 films grown on C-plane Al_2O_3 (0001) across representative growth techniques. The table compares the E_U, the rocking-curve full width at half maximum (RC-FWHM) of the β-Ga_2O_3 ($\bar{2}01$) reflection, and the atomic-force-microscopy (AFM) root-mean-square roughness (RMS). Supplementary Table 3 corresponds to the expanded version of the benchmark summarized in Table 1 of the main text.

Abbreviations used in Supplementary Table 3 are as follows: RF sputter, radio-frequency magnetron sputtering; PLD, pulsed laser deposition; MBE, molecular beam epitaxy; PAMBE, plasma-assisted molecular beam epitaxy; MOCVD, metal–organic chemical vapor deposition; PECVD, plasma-enhanced chemical vapor deposition; LPCVD, low-pressure chemical vapor deposition; and halide-VPE, halide vapor-phase epitaxy.

Method	E_U (meV)	($\bar{2}01$) RC-FWHM (°)	AFM RMS (nm)	Ref.
RF sputter (this work)	182	1.78	1.15−1.27	This work
RF sputter (literature)	280	N/A	N/A	3
Bulk	60−140	N/A	N/A	4
PLD	396	N/A	1.57−4.70	5-7
MBE	320	0.9−1.9	0.88	8-10
PAMBE	150	1.0	0.21−7	11-14
MOCVD	220–345	0.60−2.25	1.28−1.69	15-22
PECVD	N/A	0.8	4.0	23,24
LPCVD	150	1.18-1.50	1.82−3.50	25-28
mist CVD	N/A	1.131	N/A	29
halide-VPE	N/A	0.33	N/A	30

Supplementary Table 3. Expanded literature survey of optical, structural, and surface quality for β-Ga_2O_3 films across growth techniques. E_U, β-Ga_2O_3 ($\bar{2}01$) RC-FWHM, and RMS roughness are compared between this work (RF sputtering) and representative literature reports. Ranges indicate reported values across references; N/A denotes not reported.

Supplementary Note 10. Feature importance and 1D PDPs of the expanded dataset.

To examine the dominant single-parameter trends in the expanded dataset, we trained a random-forest surrogate using the 215-run dataset and evaluated impurity-based feature importance and 1D PDPs (Supplementary Fig. 8).

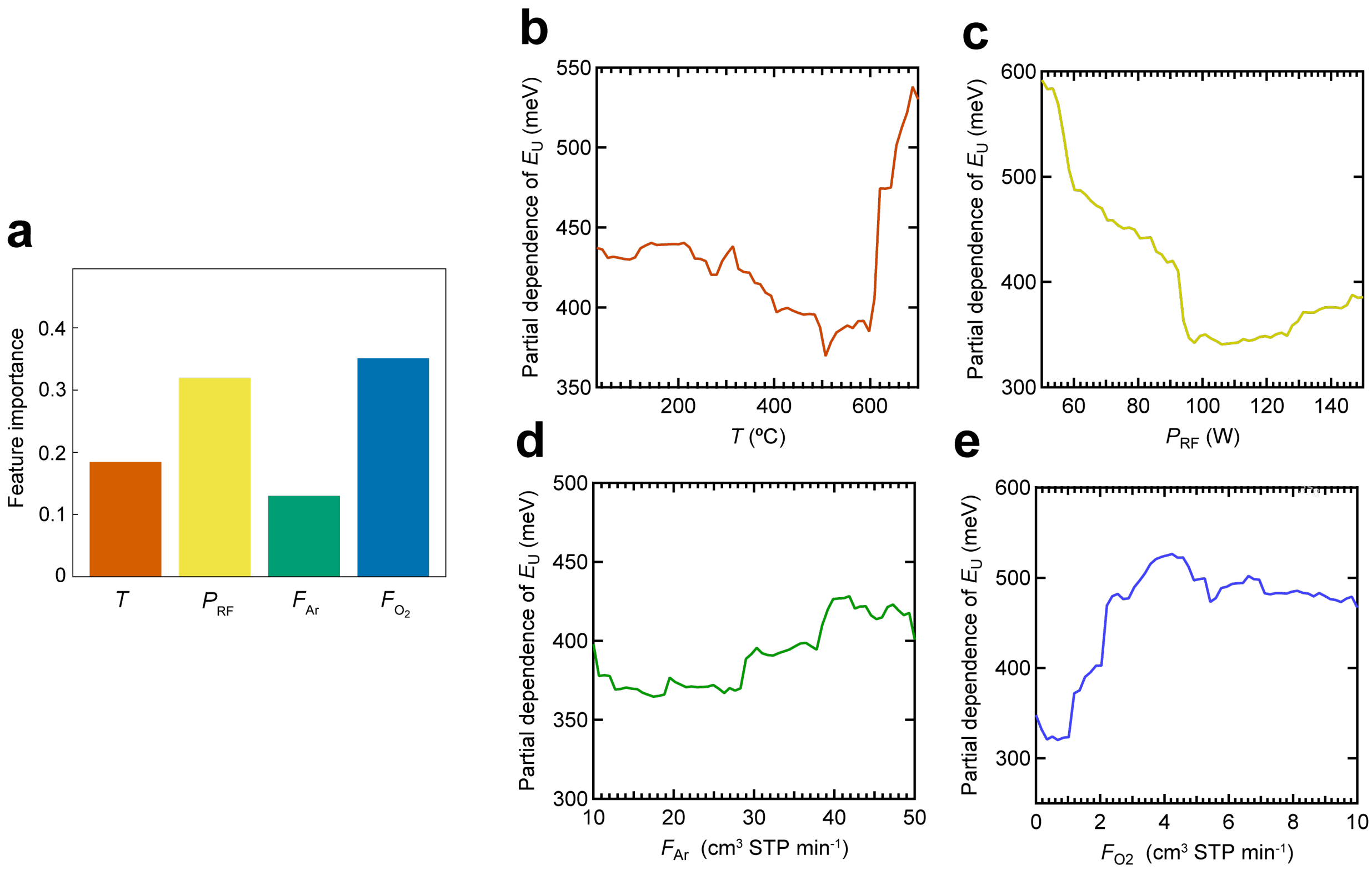

Supplementary Fig. 8. Feature importance and 1D PDPs of the expanded dataset. a Feature importance obtained from the random forest surrogate trained on the expanded 215-run dataset. **b-e** 1D PDPs from the random-forest model showing the marginal effect of **b** T, **c** P_{RF}, **d** F_{Ar}, and **e** F_{O2} on E_U.

Supplementary references

[1]Carrasco, D. et al. Temperature-Dependent Anisotropic Refractive Index in β-Ga_2O_3: Application in Interferometric Thermometers. *Nanomaterials* **13**, 1126 (2023).

[2]Penman, L. T. et al. Comparative Study of the Optical Properties of α-, β-, and κ-Ga_2O_3. Phys. *Status Solidi B* **262**, 2400615 (2025).

[3]Thapa, D., Lapp, J., Lukman, I. & Bergman, L. Ultra-wide bandgap β-Ga_2O_3 films: Optical, phonon, and temperature response properties. AIP Adv. **11**, 125022 (2021).

[4]Islam, A., Rock, N. D. & Scarpulla, M. A. Quantification of the strong, phonon-induced Urbach tails in β-Ga_2O_3 and their implications on electrical breakdown. J. Appl. Phys. **136**, 035701 (2024).

[5]Zheng, T., He, W., Wang, L., Li, J. & Zheng, S. Effect of different substrates on Si and Ta co-doped Ga_2O_3 films prepared by pulsed laser deposition. J. Cryst. Growth **533**, 125455 (2020).

[6]Leedy, K. D. et al. Highly conductive homoepitaxial Si-doped Ga_2O_3 films on (010) β-Ga_2O_3 by pulsed laser deposition. Appl. Phys. Lett. **111**, 012103 (2017).

[7]Shen, H. et al. Effect of thickness on the performance of solar blind photodetectors fabricated using PLD grown β-Ga_2O_3 thin films. J. Alloys Compd. **822**, 153419 (2020).
[8]Cai, W. et al. Effect of Growth Pressure on Properties of β-Ga_2O_3 Thin Films Grown by Molecular Beam Epitaxy. Journal of Synthetic Crystals **51**, 1152–1157 (2022).
[9]Liu, X., Guo, P., Sheng, T., Qian, L., Zhang, W. G. & Li, Y. C. β-Ga_2O_3 thin films on sapphire pre-seeded by homo-self-templated buffer layer for solar-blind UV photodetector. Opt. Mater. **51**, 203–207 (2016).
[10]Ghose, S. et al. Growth and characterization of β-Ga_2O_3 thin films by molecular beam epitaxy for deep-UV photodetectors. J. Appl. Phys. **122**, 095302 (2017).
[11]Hao, S. J., Hetzl, M., Kunzelmann, V. F., Matich, S., Sai, Q. L., Xia, C. T., Sharp, I. D. & Stutzmann, M. Sub-bandgap optical spectroscopy of epitaxial β-Ga_2O_3 thin films. Appl. Phys. Lett. **116**, 092102 (2020).
[12]Tsai, M.-Y., Bierwagen, O., White, M. E. & Speck, J. S. β-Ga_2O_3 growth by plasma-assisted molecular beam epitaxy. J. Vac. Sci. Technol. A **28**, 354–359 (2010).
[13]Qian, L.-X., Zhang, H.-F., Lai, P. T., Wu, Z.-H. & Liu, X.-Z. High-sensitivity β-Ga_2O_3 solar-blind photodetector on high-temperature pretreated c-plane sapphire substrate. Opt. Mater. Express **7**, 3643–3653 (2017).
[14]Hao, S. J., Hetzl, M., Schuster, F., Danielewicz, K., Bergmaier, A., Dollinger, G., Sai, Q. L., Xia, C. T., Hoffmann, T., Wiesinger, M., Matich, S., Aigner, W. & Stutzmann, M. Growth and characterization of β-Ga_2O_3 thin films on different substrates. J. Appl. Phys. **125**, 105701 (2019).
[15]Mun, S. J., Lee, J.-B., Lee, D. H., Ahn, H. S. & Yang, M. The influence of amorphous-to-crystalline transition on the structural and optical properties, and wet etching behavior of MOCVD-grown Ga_2O_3 thin films. Micro and Nanostructures **209**, 208403 (2026).
[16]Zhang, T. et al. Influence of O_2 pulse on the β-Ga_2O_3 films deposited by pulsed MOCVD. Ceram. Int. **48**, 8268–8275 (2022).
[17]Horng, R.-H. et al. Ion implantation effects on the characteristics of β-Ga_2O_3 epilayers grown on sapphire by MOCVD. Ceram. Int. **48**, 36425–36432 (2022).
[18]Zhuo, Y., Chen, Z., Tu, W., Ma, X., Pei, Y. & Wang, G. β-Ga_2O_3 versus ε-Ga_2O_3: Control of the crystal phase composition of gallium oxide thin film prepared by metal-organic chemical vapor deposition. Appl. Surf. Sci. **420**, 802–807 (2017).
[19]Ma, Y. et al. Effect of off-axis substrate angles on β-Ga_2O_3 thin films and solar-blind ultraviolet photodetectors grown on sapphire by MOCVD. Mater. Sci. Semicond. Process. **131**, 105856 (2021).
[20]Chen, Y. et al. The lattice distortion of β-Ga_2O_3 film grown on c-plane sapphire. J. Mater. Sci. Mater. Electron. **26**, 3231–3235 (2015).
[21]Zhang, D., Zheng, W., Lin, R. C., Li, T. T., Zhang, Z. J. & Huang, F. High quality β-Ga_2O_3 film grown with N_2O for high sensitivity solar-blind-ultraviolet photodetector with fast response speed. J. Alloys Compd. **735**, 150–154 (2018).
[22]He, T. et al. Metalorganic chemical vapor deposition heteroepitaxial β-Ga_2O_3 and black phosphorus Pn heterojunction for solar-blind ultraviolet and infrared dual-band photodetector. phys. status solidi (a) **217**, 1900861 (2020).
[23]Wu, C. et al. Systematic investigation of the growth kinetics of β-Ga_2O_3 epilayer by plasma enhanced

chemical vapor deposition. Appl. Phys. Lett. **116**, 072102 (2020).

[24]Chen, Y.-C. et al. Ga_2O_3 photodetector arrays for solar-blind imaging. J. Mater. Chem. C **7**, 2557–2562 (2019).

[25]Anwer, M. & Verma, A. LPCVD grown n-type doped β-Ga_2O_3 films on c-plane sapphire using TEOS precursor. Mater. Sci. Semicond. Process. **197**, 109697 (2025).

[26]Jiao, Y., Jiang, Q., Meng, J., Zhao, J., Yin, Z., Gao, H., Zhang, J., Deng, J. & Zhang, X. Growth and characteristics of β-Ga_2O_3 thin films on sapphire (0001) by low pressure chemical vapour deposition. Vacuum **189**, 110253 (2021).

[27]Rafique, S., Han, L., Neal, A. T., Mou, S., Tadjer, M. J., French, R. H. & Zhao, H. Heteroepitaxy of N-type β-Ga_2O_3 thin films on sapphire substrate by low pressure chemical vapor deposition. Appl. Phys. Lett. **109**, 132103 (2016).

[28]Joshi, G., Chauhan, Y. S. & Verma, A. Temperature dependence of β-Ga_2O_3 heteroepitaxy on c-plane sapphire using low pressure chemical vapor deposition. J. Alloys Compd. **883**, 160799 (2021).

[29]Cheng, Y., Zhang, C., Xu, Y., Li, Z., Chen, D., Zhu, W., Feng, Q., Xu, S., Zhang, J. & Hao, Y. Heteroepitaxial growth of β-Ga2O3 thin films on c-plane sapphire substrates with β-$(Al_xGa_{1-x})_2O_3$ intermediate buffer layer by mist-CVD method. Mater. Today Commun. **29**, 102766 (2021).

[30]Nikolaev, V. I. et al. Epitaxial growth of $(\bar{2}01)$ β-Ga_2O_3 on (0001) sapphire substrates by halide vapour phase epitaxy. Mater. Sci. Semicond. Process. **47**, 16–19 (2016).
