# Supplementary material for: Interpretable self-driving sputtering epitaxy reveals human-usable growth rules for β-Ga2O3 films
Source: Nat Commun. 2026 Aug 13;17:9714. doi: 10.1038/s41467-026-76533-0 (PMC13562525; doi:10.1038/s41467-026-76533-0)
Supplement: Supplementary file 2 [file 41467_2026_76533_MOESM2_ESM.pdf]

Description of Additional Supplementary Files.

Supplementary Movie 1. Automated sputtering sequence.
The movie shows automated wafer transfer from the load-lock to the sputtering chamber, film growth, and subsequent return of the wafer to the load-lock.

Supplementary Data 1.
Experimental datasets, including optical measurement and other materials-characterization data, together with code for automated optical analysis, Bayesian optimization, and interpretable machine-learning analyses.
