# Supplementary material for: Interpretable self-driving sputtering epitaxy reveals human-usable growth rules for β-Ga2O3 films
Source: Nat Commun. 2026 Aug 13;17:9714. doi: 10.1038/s41467-026-76533-0 (PMC13562525; doi:10.1038/s41467-026-76533-0)
Supplement: Supplementary file 4 [file 41467_2026_76533_MOESM6_ESM.pdf]

nature portfolio

Peer Review File

Interpretable self-driving sputtering epitaxy reveals human-usable growth rules for β-Ga2O3 films

Corresponding Author: Dr Yuki Wakabayashi

This file contains all reviewer reports in order by version, followed by all author rebuttals in order by version.

Version 0:

Reviewer comments:

Reviewer #1

(Remarks to the Author)
The manuscript presents an innovative and well-executed demonstration of an interpretable self-driving laboratory framework applied to RF magnetron sputtering of β-Ga_2O_3, achieving a record-low Urbach energy for sputtered films and demonstrating transferability to homoepitaxy. The work is technically sound, timely, and highly relevant to the materials science and autonomous experimentation communities. The following issues should be addressed to further strengthen the manuscript before acceptance.
(1) Both the Abstract and Discussion claim to establish a "general route," yet the manuscript does not discuss the framework's generalizability to other complex oxides or material systems, nor its potential limitations; it is recommended to add a short subsection in the Discussion briefly analyzing its applicability and constraints.
(2) In the Introduction, the statement following "most SDL implementations still function as black-box optimizers" that "This interpretability gap directly undermines reproducibility" presents an overly absolute causal claim, and the manuscript provides only limited repetition data; additional experimental evidence should be included to substantiate the reproducibility of the present study.
(3) The Introduction mentions that the optimum was reached at the 56th run (66 total closed-loop runs) but lacks quantification of the SDL's efficiency advantage in four-dimensional space; it is suggested to supplement in the Results or Discussion section the total number of experimental cycles and a comparison with the efficiency of traditional trial-and-error approaches.
(4) The main text states that all films were fixed at a thickness of 55 nm, yet the sputtering rate varies significantly with substrate temperature T, RF power PRF, and gas flows FAr/FO_2; the Methods section should clearly describe how the system automatically adjusts growth time or other parameters to achieve consistent thickness control.
(5) It is unclear how the automated optical analysis eliminates the influence of thin-film interference fringes on the Urbach energy EU fitting.
(6) It is unclear whether a pre-sputtering step is included before the automated closed-loop triggers the next growth run; the Methods section should clarify this and provide more detailed experimental procedures and parameters.
(7) Please provide the video of the experiment process.
(8) Please provide the code of the modeling and controlling program; without it, it is difficult to evaluate.

(Remarks on code availability)
The authors only provide the code for analyzing data; the code for modeling and controlling is even more important. It is necessary to provide.

Reviewer #2

(Remarks to the Author)

1) The work is “workflow novelty” rather than “scientific novelty.”
This manuscript follows a now-common template: run a standard sputtering experiment, wrap it with Bayesian optimization, then apply a post-hoc “interpretable AI” step (random forest + PDP/interaction metrics) to produce human-readable trends. The main “contribution” is essentially a change in lab habit (automation + optimization + model explanation), not a new growth mechanism, new physics, or a new materials concept. The paper reads as an implementation report of established tools on a chosen case study rather than a discovery-driven scientific advance.

2) The “interpretable growth rules” are largely trivial and not convincingly mechanistic.
The extracted “rules” reduce to expected statements: temperature is the dominant knob; other parameters behave mostly additively; only a modest T–O_2 coupling appears. This is not surprising for sputtering/epitaxy and is not demonstrated to yield new mechanistic understanding (e.g., validated defect chemistry, phase stability arguments, kinetic/thermodynamic model, or independent experiments designed to test causal hypotheses). In other words, the interpretability layer summarizes correlations in a BO-biased dataset rather than producing genuinely new, physics-grounded insights.

3) Generalization/transfer claims are not strong enough to justify publication as a methodological advance.
The “transferability” demonstrated is narrow (heteroepitaxy window applied to homoepitaxy) and still within the same material system and tool. There is no convincing evidence that the derived rules are robust across chambers, targets, thickness ranges, substrate miscut/polarity, or broader process windows. Additionally, the surrogate interpretability is trained on only 66 BO-selected samples (non-uniform sampling), which limits confidence that the reported additivity/interaction structure is stable rather than an artifact of the acquisition path. Overall, the manuscript does not establish broadly reusable methodology beyond what is already standard practice in SDL/BO literature, nor does it deliver a clear new scientific result commensurate with a high-impact publication.

(Remarks on code availability)

Reviewer #3

(Remarks to the Author)
I suggest - Major revision.
The manuscript reports a closed-loop sputter epitaxy workflow for β-Ga_2O_3 that couples Bayesian optimization (BO) with automated optical analysis (Urbach energy, EU) and post hoc surrogate-based interpretability. The experimental results appear technically plausible and the study has potential significance for autonomous thin-film process development. However, several framing and transferability claims are currently overstated or insufficiently supported, and key experimental controls/metadata required for reproducibility and for validating the optical metric across a wide process space are not yet documented at the level expected for *Nature Communications*.

1) Noteworthy results

1. Closed-loop optimization in a 4D sputter parameter space (substrate temperature, RF power, Ar flow, O_2 flow) achieving a low-disorder optical metric (EU ~182 meV) on sapphire within a moderate campaign size (tens of runs).
2. Integration of interpretability through surrogate decomposition (e.g., partial dependence/feature importance/interaction analyses) to propose a human-usable “growth window,” rather than reporting only the final optimum.
3. Attempted cross-substrate transfer from heteroepitaxy on sapphire to homoepitaxy on β-Ga_2O_3 substrates using the identified window, positioning the workflow as a practical route to high-quality epitaxial films.

These are meaningful technical outputs that could be valuable to both the sputter epitaxy community and the broader self-driving laboratory (SDL)/autonomous process optimization community.

2) Significance to the field and positioning vs literature

The work is relevant to two active areas: (i) accelerated discovery/optimization of growth conditions for complex oxides and wide-bandgap semiconductors, and (ii) closed-loop experimentation for materials synthesis and processing. The manuscript is likely of interest to researchers in thin film growth (sputtering/oxide epitaxy), autonomous experimentation, and data-driven process control.
That said, the manuscript’s broader framing around “SDLs as black boxes” should be tightened. Many SDL efforts incorporate physics-informed modeling, constrained/structured BO, and interpretability-by-design. The authors should more precisely locate the “black-box” issue in the decision/optimization layer (and/or lack of distilled transferable rules), rather than implying SDLs as a system concept are intrinsically black-box.
3) Do the data support the conclusions and claims?
Partially. The dataset and demonstrated improvement in EU support the claim that the closed-loop workflow can efficiently identify high-quality growth conditions within the explored bounds. The interpretability analysis plausibly supports qualitative growth trends (e.g., which parameters matter most within the tested region).
However, additional evidence is needed to support two central claims:
(A) “Acceleration/efficiency” beyond run count
The manuscript reports reaching a low EU after a certain number of runs, but does not provide a quantitative baseline comparison (time/experiments avoided) against conventional optimization practice. If “acceleration” is a major motivation, it should be explicitly quantified (e.g., implied size of a comparable grid/OFAT campaign within the same bounds; or a realistic expert-guided workflow) and reported as an acceleration factor or experiment/time reduction to achieve EU ≤ a defined threshold.
(B) “Transferability” to homoepitaxy
The claim that the BO-identified window is “directly transferable” from sapphire to β-Ga_2O_3 substrates is potentially high-impact but currently under-controlled. Substrate identity is not an input variable in the model; therefore, successful growth on a different substrate does not automatically demonstrate that the learned “rules” are substrate-insensitive or mechanistically transferable. At minimum, the manuscript should include a small comparative set on β-Ga_2O_3 (e.g., perturbations around the

claimed optimum in temperature and O_2 flow and/or one other knob) to demonstrate the robustness and local optimality of the window on the new substrate. Alternatively, the authors should soften the claim to an empirical observation ("the identified window was sufficient to enable homoepitaxy under our tool conditions") rather than a general statement of transferability. (Personal opinion is that the transferability is from the workflow Not from the conditions/windows).
4) Concerns about data analysis, interpretation, and conclusions
4.1 Overgeneralized "black-box SDL" statements
The manuscript would benefit from a careful rewrite of the SDL discussion. SDLs include automation, sensing, data infrastructure, and decision-making; the "black-box" critique is more appropriately applied to the BO/surrogate decision layer and to the lack of interpretable, transferable process knowledge in many reports. This distinction matters for technical accuracy and for fair positioning of the contribution.
4.2 Optical metric robustness vs thickness/optics artifacts
EU is extracted from optical data, and the manuscript indicates a nominal constant thickness (e.g., 55 nm) using pre-calibrated deposition rate to set growth time. But deposition rate can change with RF power and gas composition/flows. If thickness varies significantly across the BO campaign, EU extraction and cross-run comparability may be biased (e.g., interference fringes, absorption path length changes, or analysis assumptions). The authors should:
Provide thickness measurements for a representative subset across the explored parameter space (or ideally all runs) using profilometry/ellipsometry/XRR/cross-sectional microscopy;
Quantify thickness variation and evaluate whether EU correlates with thickness;
Clarify whether "55 nm" is measured or nominal, and how uncertainties propagate into EU.
4.3 Interpretability vs "physics-informed" wording
The interpretability in this manuscript appears to come from post hoc surrogate interrogation (e.g., random forest feature importance and PDP analyses). That is valuable, but it is distinct from "physics-informed ML" (constraints/priors/structure embedded in the model). The authors should clearly state what is physics-informed (if anything) versus what is post hoc interpretability, and avoid conflating the two.
5) Methodology soundness and expected standards
The overall methodology—closed-loop BO with an automated objective and subsequent interpretability analysis—is sound and aligned with current best practices. The experiment design and the multi-parameter search are appropriate.
However, the manuscript should strengthen:
Uncertainty quantification(replicate measurements and run-to-run variability, especially near the optimum);
Objective validity (EU extraction details and robustness checks);
Comparability controls (thickness verification, instrument calibration stability, and handling of measurement noise/outliers).
we require revision to meet the standard of evidence for broad claims (efficiency + transferability).

(Remarks on code availability)
Supporting code is provided with the submission; however, it is relatively general and does not substantially impact my evaluation of the experimental results or the main claims.

Reviewer #4

(Remarks to the Author)
Authors demonstrate an SDL that optimizes the growth of very high quality (as measured by the Urbach energy) β-Ga2O3 films, achieving an E_U as low as ~180 meV. Authors also show that the optimal growth conditions for heteroepitaxial growth can be transferred to homoepitaxial conditions. These achievements are notable because the films are grown by RF sputtering, which is a much more scalable method compared to conventional CVD or MBE.

Overall, I find this manuscript to be of high quality, and commend the authors for its clarity. However, there are a few areas which could be improved.

1. Authors repeatedly state that BO models are "black boxes" to support their explainability work. This is misleading. Both RFS and GPs can be used in BO algorithms, and both ML algorithms are explainable: RFs through impurity, Gini, or SHAP scores; and GPs through lengthscales. In fact, the authors perform both these analyses in the manuscript.

2. Authors state that their approach constitutes "interpretable" ML. This is inaccurate. Interpretable models are those that are inherently understandable (e.g., linear regression, decision trees). These statements should be changed to "explainable" ML: post hoc analysis of models.

3. I think that the transferability of growth conditions from heteroepitaxial to homoepitaxial should be explained more thoroughly, especially for a paper in a more generalist journal like Nat Comms. How likely is it that the transferability is simply coincidental, rather than the BO algorithm finding the best growth conditions regardless of substrate?

4. How novel is the discovery that T is the most important parameter for growth of high-quality β-Ga2O3 films? I believe this is reasonably well-understood across different fields since higher temperatures provide more reorganization energy. Furthermore, the choice to do the first seven runs with fixed P_RF, F_Ar and F_O2 while varying T suggests that this may have been known ahead of time. I would imagine that if the response surface was truly unknown, then choosing a seed dataset by DOE would have been a stronger approach.

5. I don't fully understand why the authors chose to use a RF model for the explainability section of the paper. A GP can be

"explained" just as well as an RF, and the GP is already what's used in the BO algorithm. So explaining what the GP learned would be more informative as to the BO process anyway. Authors justify the selection of the RF because it has a higher in-sample (i.e. training) R^2 score (0.92 for RF vs 0.76 for GP). However, the RF's 5-fold CV test score is much work (0.43). This suggests that the RF is overfitting to the data, which is more likely given that the tree depth hyperparameter is set to unlimited depth... What is the 5-fold CV test score of the GP? It may provide a stronger basis for explainability.

Minor note:
The plots in Fig 6a-c are difficult to interpret in 3D. I suggest replacing them with 2D plots where PDP of E_U is represented by a perceptually uniform color scale.

(Remarks on code availability)
The data and code for the explainable ML portion of the paper is clear and easy to follow. However, only the optical analysis code and data from the SDL portion of the paper is provided. I strongly encourage the authors to share at least their Bayesian optimization code. Also sharing the code for running their SDL would make it much easier to repeat/reproduce such work in the future, and continue to help the community grow.

Version 1:

Reviewer comments:

Reviewer #1

(Remarks to the Author)
The authors demonstrated an interpretable self-driving laboratory framework that transforms autonomous optimization data into human-executable growth rules. I have read the updated manuscript, and I still have the following major concerns for the paper:
1. Is it still not clear how substrate temperature T, RF power PRF, and gas flows and F_{Ar}/F_{O_2} were decided by the machine learning model.
2. I agree with Reviewer 2 that the interpretability layer summarizes correlations in a BO-biased dataset rather than producing genuinely new, physics-grounded insights. It is not physically interpretable but mathematically interpretable.
3. The “transferability” demonstrated is indeed narrow. No other material system or deposition tools were involved.
4. The authors choose to do the first seven runs with fixed P_{RF}, F_{Ar}, and F_{O2} while varying T, which already suggests that T is the most important parameter for growth.

Given these major concerns, I think the paper is not yet at the level expected by Nature Communications.

(Remarks on code availability)
I have run the codes myself, the results can fit the ones mentioned in the manuscript.

Reviewer #2

(Remarks to the Author)
I think the authors has well responded , I have no further questions.

(Remarks on code availability)

Reviewer #3

(Remarks to the Author)
The authors have significantly improved the manuscript by adding new experiments, expanded analysis, and clearer methodological details. I think the revised work is technically sound, but a few claims should be tightened before acceptance.

First, the “long-standing challenge” should be framed more specifically. High-quality β-Ga_2O_3 epitaxy is already well established by other methods, so the novelty should be stated as achieving low-disorder, single-crystalline β-Ga_2O_3 epitaxy by scalable RF magnetron sputtering.

Second, the manuscript should better separate automation throughput from algorithmic acceleration. The BO workflow reaches $E_U \leq 182$ meV at run 56, while the human-executed strategy reaches a similar level at about run 65. This suggests that the run-count advantage is modest, and the main practical gain may come from automation-enabled throughput.

Finally, the wording around “self-driving laboratories” and “black-box” optimization should be more precise. SDLs are not inherently black boxes; the black-box aspect mainly comes from the BO/surrogate decision layer. I suggest revising the abstract and related text to focus on closed-loop BO workflows rather than SDLs in general. especially the first sentence is difficult to understand and very confusing.

After these relatively minor wording and framing revisions, I would support publication.

(Remarks on code availability)

Reviewer #4

(Remarks to the Author)
In my opinion, authors have adequately addressed reviewer comments.

(Remarks on code availability)

Version 2:

Reviewer comments:

Reviewer #1

(Remarks to the Author)
The authors have clarified most issues. I only have one comment: to be accurate, I think the authors need to update the sentence in the abstract "we demonstrate an interpretable self-driving laboratory framework" to "we demonstrate a mathematically interpretable self-driving laboratory framework" .

With this, I think the manuscript is ready to publish.

(Remarks on code availability)

Reviewer #3

(Remarks to the Author)
I appreciate the authors' careful and comprehensive responses to the reviewers' comments. The manuscript has been substantially improved. The revised version now clearly distinguishes the Bayesian optimization workflow from the post hoc interpretability analysis, appropriately limits the claims regarding transferability and physical interpretation, and more accurately frames the contribution of the work. The clarification separating automation-enabled throughput from algorithmic acceleration is also helpful.

I have only two minor editorial suggestions. First, the repeated phrase "mathematically interpretable and human-executable process rules" could be simplified to improve readability. Second, in a few places where the manuscript discusses additive parameter contributions, slightly softer wording (e.g., "the surrogate indicates" or "is well approximated by") would better distinguish empirical observations from mechanistic conclusions.

These are minor wording suggestions only and do not affect the scientific conclusions. I believe the manuscript is suitable for publication after minor editorial revision.

(Remarks on code availability)

Open Access This Peer Review File is licensed under a Creative Commons Attribution 4.0 International License, which permits use, sharing, adaptation, distribution and reproduction in any medium or format, as long as you give appropriate credit to the original author(s) and the source, provide a link to the Creative Commons license, and indicate if changes were made.
In cases where reviewers are anonymous, credit should be given to 'Anonymous Referee' and the source.
The images or other third party material in this Peer Review File are included in the article's Creative Commons license, unless indicated otherwise in a credit line to the material. If material is not included in the article's Creative Commons license and your intended use is not permitted by statutory regulation or exceeds the permitted use, you will need to obtain permission directly from the copyright holder.
To view a copy of this license, visit https://creativecommons.org/licenses/by/4.0/

Response letter

We would like to express our gratitude for considering our manuscript (#NCOMMS-26-010832) for publication in Nature Communications. We are also thankful to the reviewers for the careful reading of our manuscript and the valuable comments that helped us improve its quality. We have revised the manuscript in accordance with the reviewers' comments; the revised parts are indicated in red letters in the revised manuscript. The following are our point-by-point responses to the reviewers' comments.

Response to the comments of the reviewer #1

Comment 1:

The manuscript presents an innovative and well-executed demonstration of an interpretable self-driving laboratory framework applied to RF magnetron sputtering of β-Ga_2O_3, achieving a record-low Urbach energy for sputtered films and demonstrating transferability to homoepitaxy. The work is technically sound, timely, and highly relevant to the materials science and autonomous experimentation communities. The following issues should be addressed to further strengthen the manuscript before acceptance.

Response:

We thank the reviewer for the positive and encouraging assessment of our work. We appreciate the reviewer's recognition of the significance, timeliness, and technical soundness of the study. In response to the reviewer's constructive suggestions, we have substantially revised the manuscript by adding new experimental data, expanded analyses, and additional methodological details to improve the clarity, reproducibility, and robustness of the work. We address each of the reviewer's specific comments in detail below.

Comment 2:

(1) Both the Abstract and Discussion claim to establish a "general route," yet the manuscript does not discuss the framework's generalizability to other complex oxides or material systems, nor its potential limitations; it is recommended to add a short subsection in the Discussion briefly analyzing its applicability and constraints.

Response:

Following the reviewer's suggestion, in the revised Discussion, we added a short subsection to clarify the applicability and limitations of the present framework [(page 16, line 24) in the revised manuscript]. We clarify that the overall workflow, which combines autonomous synthesis with post hoc explainable machine-learning analysis to distill human-usable growth rules, can in principle be adapted to other material systems and synthesis methods. In particular, for insulating materials with an optical band gap, where film quality can be evaluated through automated optical spectroscopy and analysis as in the present study, the framework can be transferred rather directly. By contrast, for material systems such as metals or superconductors, for which an analogous optical quality metric is not readily available, the autonomous loop would need to incorporate alternative automated evaluation modules, such as crystallographic characterization by X-ray diffraction or electrical transport characterization. A key practical constraint for such a framework is experimental reproducibility, because the prediction accuracy of both the Bayesian optimization (BO) surrogate and the post hoc explainable model is ultimately limited by the reproducibility of the underlying experimental data. These points have now been added to the Discussion to better position the generality and limitations of the present approach [(page 16, line 24) in the revised manuscript].

Comment 3:

(2) In the Introduction, the statement following "most SDL implementations still function as black-box optimizers" that "This interpretability gap directly undermines reproducibility" presents an overly absolute causal claim, and the manuscript provides only limited repetition data; additional experimental evidence should be included to substantiate the reproducibility of the present study.

Response:

To strengthen the evidence for reproducibility, we added both replicate growth experiments across representative Urbach energy (E_U) regimes and an independent human-executed re-optimization based on the extracted growth rule, as described below. The former additional experiments validate the experimental reproducibility of the measured optical metric, whereas the latter demonstrates that the extracted strategy is practically executable

and can reproducibly reach the low-E_U region identified by the original BO campaign. In addition, the original causal claim was overly strong, and we therefore removed the word "directly" from the relevant sentence in the Introduction [(page 2, line 10) in the revised manuscript].

To substantiate the reproducibility of the present study, we added replicate experiments not only near the optimum but also at several representative conditions yielding different E_U values. These additional experiments show that the measured E_U values are reproducible across low- and high-E_U growth regimes (Table R1). For example, the BO-identified optimum condition yielded E_U = 182 and 184 meV in two independent runs, representative nearby low-E_U conditions gave E_U values in the range of 197-219 meV, with a combined mean of 205 ± 9 meV across several nearby conditions, and an off-optimal condition reproducibly yielded much larger E_U values of 338 and 376 meV, confirming that the observed differences in E_U reflect systematic growth-condition dependence rather than incidental run-to-run scatter. We have described this point in the revised manuscript (page 5, line 13) and in the revised Supplementary Information (page 4, line 1).

Separately, a human experimenter independently carried out sequential 1D optimization followed by 2D fine-tuning based on the extracted growth rule already presented in the original manuscript, and confirmed that this human-executable strategy can reproduce the low-E_U region originally reached by the original BO campaign. In the original manuscript, the interpretable analysis of the random forest model trained on the $N = 66$ BO-acquired dataset had revealed a simple structure in which the effects of the four growth parameters are largely additive, with the T-O_2 coupling defining the narrow window for high-quality growth. This structure suggested a human-usable optimization strategy consisting of sequential one-dimensional tuning of the individual parameters, followed by a final two-dimensional refinement in the T-O_2 plane around the then-current optimum obtained from the 1D steps. Following this strategy, we performed sequential 1D optimization in the order of T, P_{RF}, F_{Ar}, and F_{O2}, and finally carried out optimization in the T-O_2 plane around the optimized T and F_{O_2} values obtained after the 1D steps (Fig. R1). Using this procedure, we reached the BO-identified optimum level of $E_U \leq 182$ meV in 65 runs, and finally obtained a minimum E_U of 163 meV after 85 runs, which is 19 meV lower than the original BO optimum. This result indicates that the extracted strategy, distilled from the interpretable analysis, can guide targeted experiments beyond merely

reproducing the BO result and can even surpass the performance reached in the original BO campaign. We have described this point in the revised manuscript (page 12, line 8).

Growth condition	T (°C)	P_{RF} (W)	F_{Ar} (sccm)	F_{O_2} (sccm)	E_U (meV)
BO-identified optimum	507	119	26	1	182, 184
Nearby low-E_U condition A	510	97	26.5	0.6	203, 199
Nearby low-E_U condition B	520	97	26.5	0.4	216, 219, 197
Nearby low-E_U condition C	512	105	26.5	0.7	198, 203
Off-optimal condition	512	100	30	5	338, 376

Table R1. Replicate growth runs used to evaluate the reproducibility of E_U across representative growth regimes. The table includes repeated runs for the BO-identified optimum, several nearby low-E_U conditions, and a clearly off-optimal condition. The nearby low-E_U conditions A-C represent multiple distinct conditions close to the optimum.

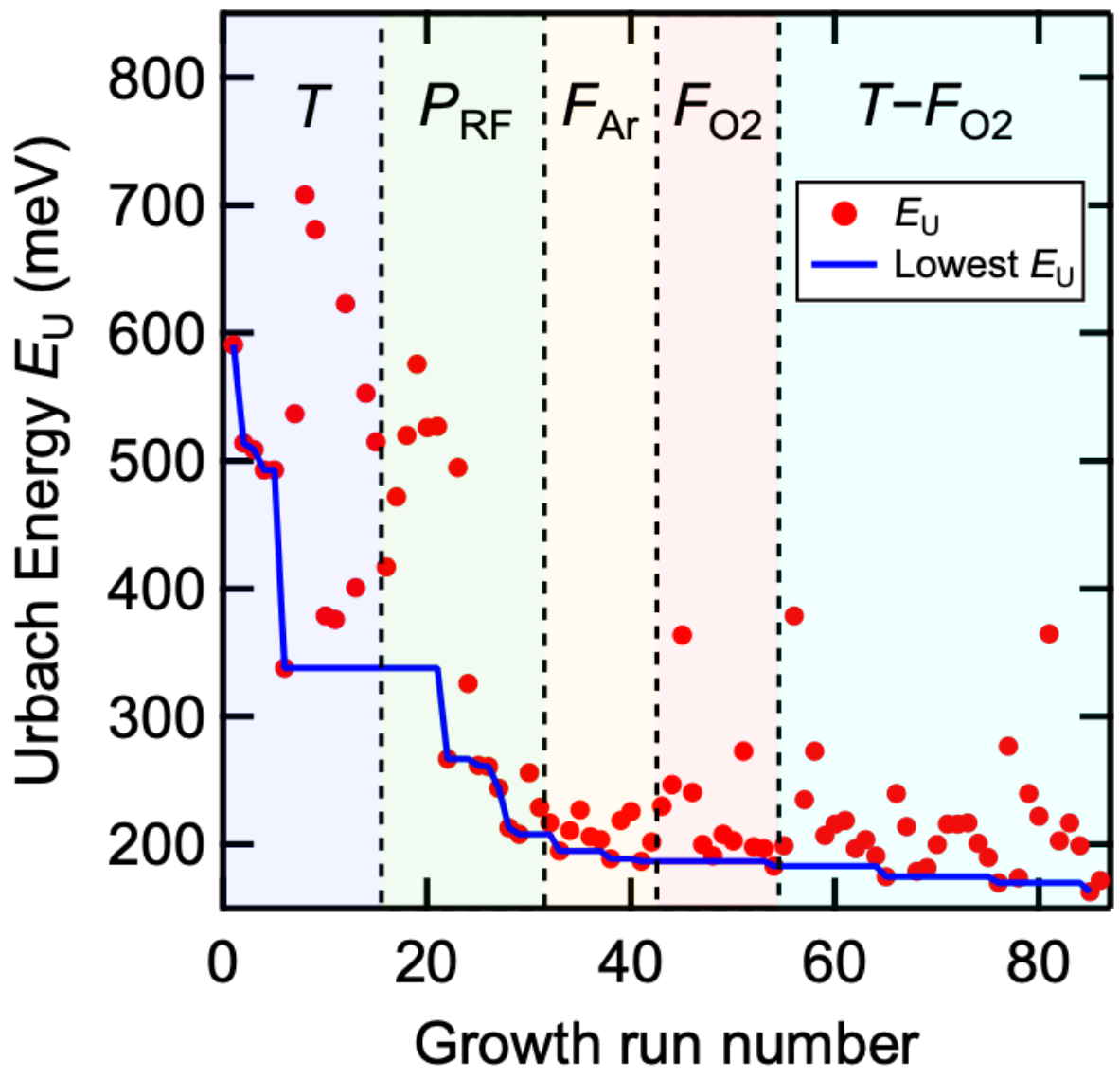

Fig. R1. Human-executed re-optimization following the extracted optimization strategy. A human experimenter performed sequential 1D optimization of T, P_{RF}, F_{Ar}, and F_{O2}, followed by 2D fine-tuning in the T-O_2 plane. The blue, green, yellow, red, and cyan shaded regions indicate the optimization stages for T, P_{RF}, F_{Ar}, F_{O2}, and T-O_2, respectively. Red circles represent the experimental E_U as a function of growth run number. The blue line indicates the lowest E_U achieved up to each run.

Comment 4:

(3) The Introduction mentions that the optimum was reached at the 56th run (66 total closed-loop runs) but lacks quantification of the SDL's efficiency advantage in four-dimensional space; it is suggested to supplement in the Results or Discussion section the total number of experimental cycles and a comparison with the efficiency of traditional trial-and-error approaches.

Response:

We agree that reporting only the run number does not sufficiently convey the efficiency advantage of the SDL in a four-dimensional search space. In the revised manuscript, we therefore added a more explicit quantification of optimization efficiency in terms of both experimental cycles and elapsed working time. Specifically, the original BO campaign reached the optimum by the 56th run, which corresponds to approximately 8 working days under the throughput of our SDL workflow (7-8 runs per day).

We also added a comparison based on the previously described human-executable optimization strategy shown in Fig. R1. This provides a more practical benchmark than a vague comparison with conventional trial-and-error, while still allowing us to quantify the reduction in experimental effort enabled by the closed-loop SDL framework. This human-executable strategy required 65 runs to reach the BO-identified optimum level of $E_U \leq 182$ meV. Under a conventional non-automated workflow, with manual sputter growth and optical characterization at a throughput of about 3 runs per day, this would correspond to approximately 22 working days, highlighting the practical efficiency advantage of the present SDL approach. We note that this is a conservative and practically defined benchmark, because the human strategy already benefits from the distilled rule; unguided trial-and-error in the same four-dimensional space would likely require substantially more trials. We have described these points in the revised manuscript (page 12, line 25) and in the revised Supplementary Information (page 2, line 14).

Comment 5:

(4) The main text states that all films were fixed at a thickness of 55 nm, yet the sputtering rate varies significantly with substrate temperature T, RF power PRF, and gas flows FAr/FO_2; the Methods section should clearly describe how the system automatically adjusts growth time or other parameters to achieve consistent thickness control.

Response:

Following the reviewer's suggestion, we clarified in the revised manuscript how the growth time was automatically adjusted to achieve consistent thickness control. Specifically, the deposition time was determined for each run so as to target a nominal thickness of 55 nm, using pre-calibrated deposition-rate data measured at room temperature. Table R2 summarizes the deposition rates calculated from the film thicknesses measured by a stylus profiler after deposition under various P_{RF}, F_{Ar}, and F_{O2} conditions. At P_{RF} = 100 W, the deposition rate does not depend strongly on F_{Ar} or F_{O2} and remains in the range of 0.80-0.89 nm/min. By contrast, as P_{RF} increases from 60 to 130 W, the deposition rate increases systematically from 0.64 to 1.42 nm/min. Therefore, we estimated the deposition rate from an empirical quadratic fit to the rate-versus-P_{RF} calibration curve and automatically determined the growth time accordingly for each run (Fig. R2). In addition,

regarding the effect of T, the actual thickness of the film grown under the condition (T, P_{RF}, F_{Ar}, $F_{\mathrm{O_2}}$) = (507°C, 119 W, 26 sccm, 1 sccm) was estimated from STEM observation (Fig. 4 in the revised manuscript) to be 56 ± 2 nm, which is in good agreement with the target nominal thickness of 55 nm. This agreement suggests that, at least near the optimized high-quality growth window, temperature-dependent changes in the effective deposition rate do not significantly affect the nominal thickness control. We have described this point in the revised manuscript (page 18, line 17) and in the revised Supplementary Information (page 3, line 1).

P_{RF} (W)	F_{Ar} (sccm)	$F_{\mathrm{O_2}}$ (sccm)	Rate (nm/min)
60	9	0	0.64
80	9	0	0.72
100	6	0	0.84
100	9	0	0.89
100	12	0	0.86
100	30	0	0.83
100	30	1	0.8
100	30	5	0.81
119	26	1	1.18
130	30	0	1.42

Table R2. Pre-calibrated deposition-rate data used for nominal thickness control. The table summarizes the deposition rates estimated from film thicknesses measured by a stylus profiler after deposition under representative P_{RF}, F_{Ar}, and F_{O2} conditions.

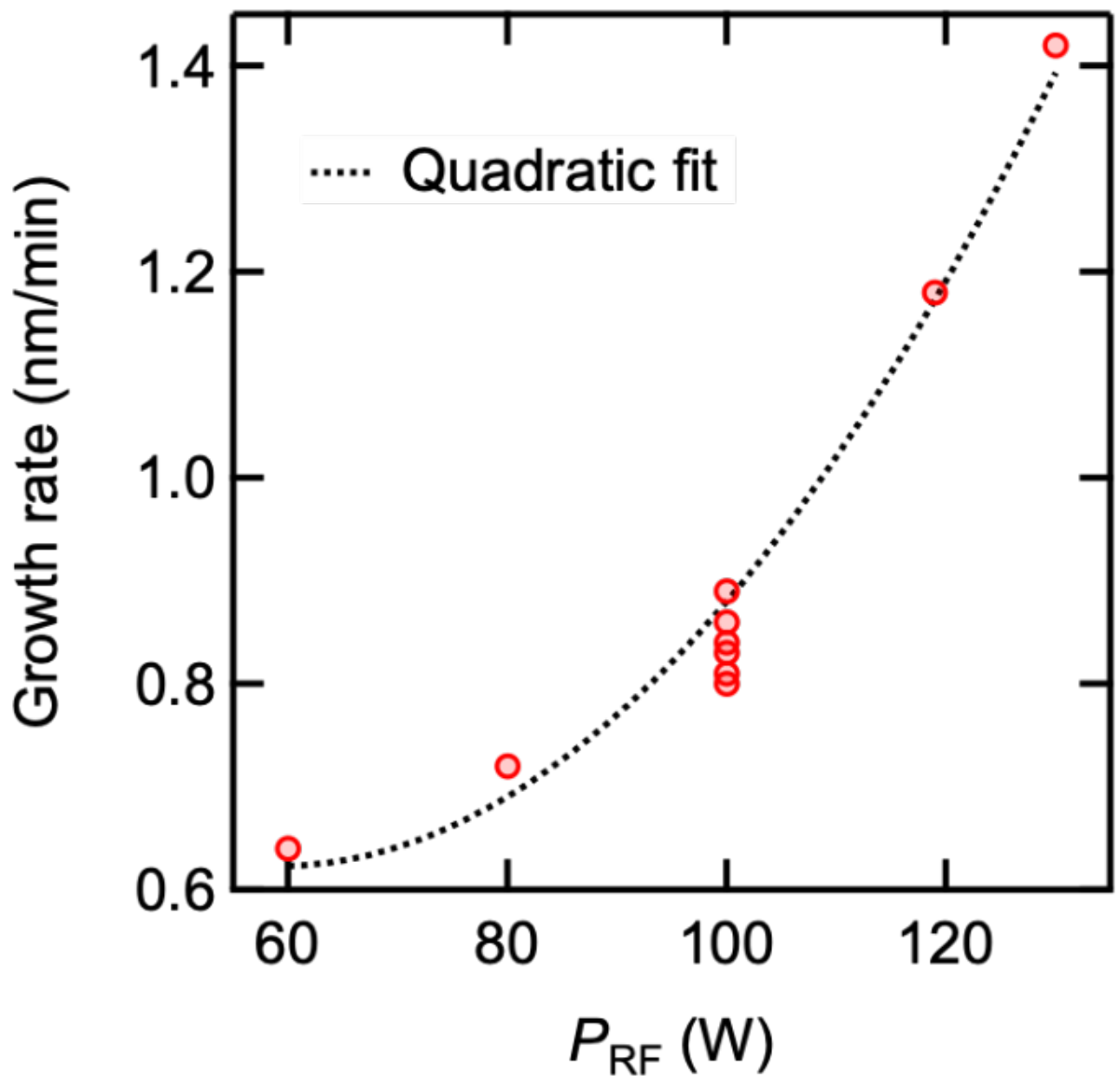

Fig. R2. Deposition-rate calibration used for nominal thickness control. Deposition rate as a function of P_{RF}, using the data in Table R2. The solid curve is the empirical quadratic fit.

Comment 6:

(5) It is unclear how the automated optical analysis eliminates the influence of thin-film interference fringes on the Urbach energy EU fitting.

Response:

We thank the reviewer for raising this important point. In the present analysis, the influence of thin-film interference on the extracted E_U is expected to be negligible. The β-Ga_2O_3 films used for the closed-loop optimization have a thickness of only 55 nm. Taking a representative refractive index of β-Ga_2O_3 in the visible/near-UV range, $n \approx 1.9$–2.0, the optical thickness of the 55-nm film is $2nd \approx 210$–220 nm[R1,R2], where d is the film thickness. The approximate number of interference oscillations (N) over a measured wavelength range can be estimated from the standard thin-film interference condition, $2nd = m\lambda$, where m is the interference order and λ is the wavelength, as $N \approx 2nd\,(1/\lambda_{min} - 1/\lambda_{max})$[R3]. Therefore, over the measured wavelength range of 190–600 nm, the expected interference modulation corresponds to less than one oscillation (N = 0.75–0.80), and within the narrow energy window used for the Urbach-tail fitting just below band gap energy (Eg), it appears only as a slowly varying background rather than as pronounced interference fringes. In fact, as

shown in the representative $ln[\alpha(h\nu)]$ spectrum in Fig. R3, $ln[\alpha(h\nu)]$ exhibits a clear linear dependence on photon energy $h\nu$ in the extracted Urbach-tail region immediately below E_g, as expected for an exponential Urbach tail, where $\alpha(h\nu)$ is the absorption coefficient. Therefore, we conclude that thin-film interference does not significantly affect the extracted E_U under the present experimental conditions. We have described this point in the revised Supplementary Information (page 10, line 10).

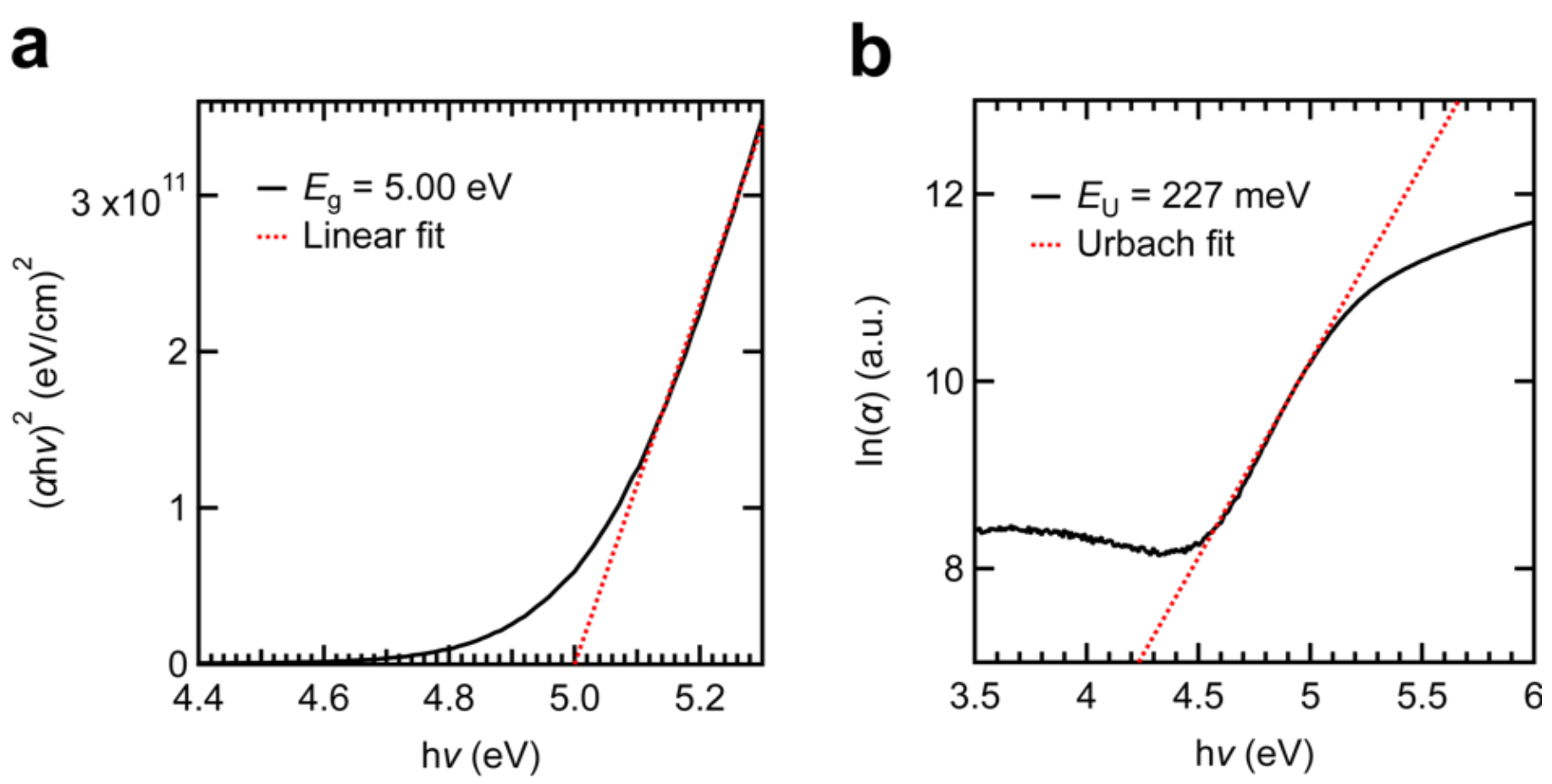

Fig. R3. Representative procedure for optical extraction of E_g and E_U of the β-Ga_2O_3 film with E_g = 5.00 eV and E_U = 227 meV on C-plane Al_2O_3. a Tauc plot $(\alpha h\nu)^2$ as a function of photon energy $h\nu$, with a linear fit used to estimate E_g. **b** Urbach energy analysis shown as $\ln(\alpha)$ versus $h\nu$ with a linear fit in the exponential tail region used to extract E_U.

[R1] Carrasco, D. et al. Temperature-Dependent Anisotropic Refractive Index in β-Ga_2O_3: Application in Interferometric Thermometers. *Nanomaterials* **13**, 1126 (2023).

[R2] Penman, L. T. et al. Comparative Study of the Optical Properties of α-, β-, and κ-Ga_2O_3. Phys. *Status Solidi B* **262**, 2400615 (2025).

[R3] Fox, M. Optical Properties of Solids 2nd edn. (Oxford Univ. Press, Oxford, 2010).

Comment 7:

(6) It is unclear whether a pre-sputtering step is included before the automated closed-loop triggers the next growth run; the Methods section should clarify this and provide more detailed experimental procedures and parameters.

Response:

Thank you for pointing that out. Following the reviewer's suggestion, we have clarified this point in the revised Methods section. Before each growth run, pre-sputtering, i.e., target conditioning, was performed for 1 min with the sputtering shutter closed. During this pre-sputtering step, the substrate temperature, Ar/O_2 gas flow rates, and RF power were set to the same values as those used for the corresponding growth run. After pre-sputtering, the shutter was opened and film growth was initiated under the same conditions. This pre-sputtering step was included in the automated growth sequence for every BO-suggested run. We have described this point in the revised manuscript (page 18, line 12).

Comment 8:

(7) Please provide the video of the experiment process.

Response:

Following the reviewer's suggestion, we have added a video showing the automated sputtering growth sequence, including wafer transfer and growth operation, as Supplementary Video.

Comment 9:

(8) Please provide the code of the modeling and controlling program; without it, it is difficult to evaluate.

(Remarks on code availability)

The authors only provide the code for analyzing data; the code for modeling and controlling is even more important. It is necessary to provide.

Response:

In response to this comment, we have additionally provided the source code used for the closed-loop BO workflow, including surrogate-model training, acquisition-function optimization, and next-condition proposal. In the original submission, we provided the code for optical spectral analysis, including EU extraction. Together, these codes enable

reproduction of the modeling and optimization workflow from the measured optical spectra to the BO-suggested next growth conditions.

The low-level equipment-control software for the automated sputtering and optical-measurement systems, including vendor-specific drivers, communication protocols, and proprietary instrument-control APIs, cannot be made publicly available. However, the closed-loop decision logic that determines the next growth condition has now been provided, and we have clarified the instrument model, automated growth sequence, pre-sputtering step, and detailed experimental procedures in the revised Methods [(page 17, line 32) in the revised manuscript]. These additions ensure that the modeling and optimization workflow, as well as the experimental sequence, can be evaluated and reproduced.

Response to the comments of the reviewer #2

Comment 1:

1) The work is "workflow novelty" rather than "scientific novelty."

This manuscript follows a now-common template: run a standard sputtering experiment, wrap it with Bayesian optimization, then apply a post-hoc "interpretable AI" step (random forest + PDP/interaction metrics) to produce human-readable trends. The main "contribution" is essentially a change in lab habit (automation + optimization + model explanation), not a new growth mechanism, new physics, or a new materials concept. The paper reads as an implementation report of established tools on a chosen case study rather than a discovery-driven scientific advance.

Response:

As the reviewer points out, Bayesian optimization (BO), automated experimentation, and post hoc surrogate-model interpretation are individually established methods. However, the advance of the present study is not the mere combination of these tools, but their use to realize a new sputter-epitaxy capability for β-Ga_2O_3 and to convert closed-loop optimization data into experimentally validated, human-executable growth rules. In this revision, we further strengthened this point by expanding the experimental dataset by approximately threefold and validating the extracted rules through human-executed re-optimization.

First, from the materials-science perspective, this framework realized a long-standing challenge in sputtering: high-quality epitaxial β-Ga_2O_3 growth, including single-crystalline β-Ga_2O_3 homoepitaxy by RF magnetron sputtering. RF sputtering is an industrially important, scalable deposition method, but sputtered Ga_2O_3 films have generally been limited to amorphous, polycrystalline, mixed-phase, or strongly textured films. In contrast, the present closed-loop optimization identified a low-disorder growth window that yielded β-Ga_2O_3 heteroepitaxial films with Urbach energy (E_U) = 182 meV, the lowest value reported for sputtered β-Ga_2O_3 films, together with high crystallographic quality. The same low-E_U window also enabled high-quality β-Ga_2O_3 homoepitaxy. Thus, the work is not simply an implementation of optimization workflow, but demonstrates a new sputter-epitaxy capability for an ultra-wide-bandgap semiconductor relevant to power-electronics applications.

Second, from the self-driving-laboratory perspective, the contribution is not merely that we applied a post hoc "interpretable AI" analysis to a BO dataset. The key point is that the closed-loop dataset was distilled into a human-executable growth strategy and that this strategy was then experimentally validated. Specifically, the surrogate-based analysis suggested a practical optimization strategy consisting of sequential one-dimensional tuning of individual growth parameters followed by focused two-dimensional refinement in the T–O_2 plane. In the revised manuscript, we have added a new human-executed re-optimization experiment based on this extracted strategy [(page 12, line 8) in the revised manuscript]. This experiment reproduced the low-E_U region identified by BO, reached the original BO optimum level of $E_U \leq 182$ meV, and finally achieved $E_U = 163$ meV, lower than the original BO optimum. This result demonstrates that the extracted rule is not merely a descriptive correlation in the BO dataset, but an actionable process strategy that can be executed by a human experimenter and used to guide further optimization. Together, these results show that the present work goes beyond a change in laboratory workflow: it provides both a materials-science advance in β-Ga_2O_3 sputter epitaxy and a self-driving-laboratory advance in translating autonomous optimization into experimentally validated, human-executable growth rules.

Comment 2:

2) The "interpretable growth rules" are largely trivial and not convincingly mechanistic. The extracted "rules" reduce to expected statements: temperature is the dominant knob; other parameters behave mostly additively; only a modest T–O_2 coupling appears. This is not surprising for sputtering/epitaxy and is not demonstrated to yield new mechanistic understanding (e.g., validated defect chemistry, phase stability arguments, kinetic/thermodynamic model, or independent experiments designed to test causal hypotheses). In other words, the interpretability layer summarizes correlations in a BO-biased dataset rather than producing genuinely new, physics-grounded insights.

Response:

We appreciate the reviewer's concern regarding the distinction between mechanistic interpretation and data-driven growth-rule extraction. The purpose of the explainable surrogate analysis in this study is not to derive a microscopic growth mechanism

deductively from defect chemistry, phase stability, or kinetic/thermodynamic modeling. Rather, its purpose is to quantify the experimentally observed high-dimensional sputter-growth landscape and to distill it into a human-executable strategy that can be experimentally implemented and validated.

In the original BO-acquired 66-run dataset, the key result was not simply that temperature matters, but that high-quality β-Ga_2O_3 sputter epitaxy is achieved within a multi-parameter window in which the effects of individual growth parameters are largely additive, with a residual T–O_2 coupling that further delineates the low-disorder region. We agree that experienced researchers may qualitatively anticipate this trend; however, such intuition does not by itself provide a quantitative map of the low-disorder growth window or a concrete optimization strategy in a four-dimensional process space. The random-forest surrogate analysis of the BO-acquired 66-run dataset provided this quantitative description and suggested a practical strategy for approaching the low-E_U region.

In the revised manuscript, we further addressed the stability of this interpretation by expanding the dataset from 66 to 215 runs through additional sampling and rule-based re-optimization experiments. This expanded dataset reduced the dependence on the original BO trajectory and led to a more balanced picture of the growth landscape, in which high-quality growth is governed by the combined effects of oxygen supply, RF power, substrate temperature, and Ar flow rather than by temperature alone, as described in detail in our response to Comment 3. Importantly, however, the central rule extracted from the original dataset—the largely additive structure of the growth-parameter dependence with a residual T–O_2 coupling—was retained in the expanded dataset. We have described this point in the revised manuscript (page 13, line 27).

To directly address the reviewer's concern that the extracted rule was not supported by independent experiments, we added a human-executed re-optimization experiment to validate the rule as an optimization strategy, rather than only describe correlations in a BO-biased dataset. Based on the distilled rule, a human experimenter carried out sequential one-dimensional optimization of the individual growth parameters followed by focused two-dimensional refinement in the T–O_2 plane. This human-executed strategy reproduced the low-E_U region identified by BO, reached the original BO optimum level of $E_U \leq 182$ meV, and finally achieved $E_U = 163$ meV, even lower than the original BO optimum (Fig. R4). These results show that the extracted growth rule is not merely a

descriptive correlation in the BO dataset, but an actionable and experimentally validated optimization strategy. We have described this point in the revised manuscript (page 12, line 8).

Thus, while the present study does not claim to establish a complete microscopic defect-formation mechanism, it provides experimentally constrained growth knowledge that can be executed by human researchers and can serve as a quantitative basis for future mechanistic studies integrating defect chemistry, phase stability, and surface-kinetic models.

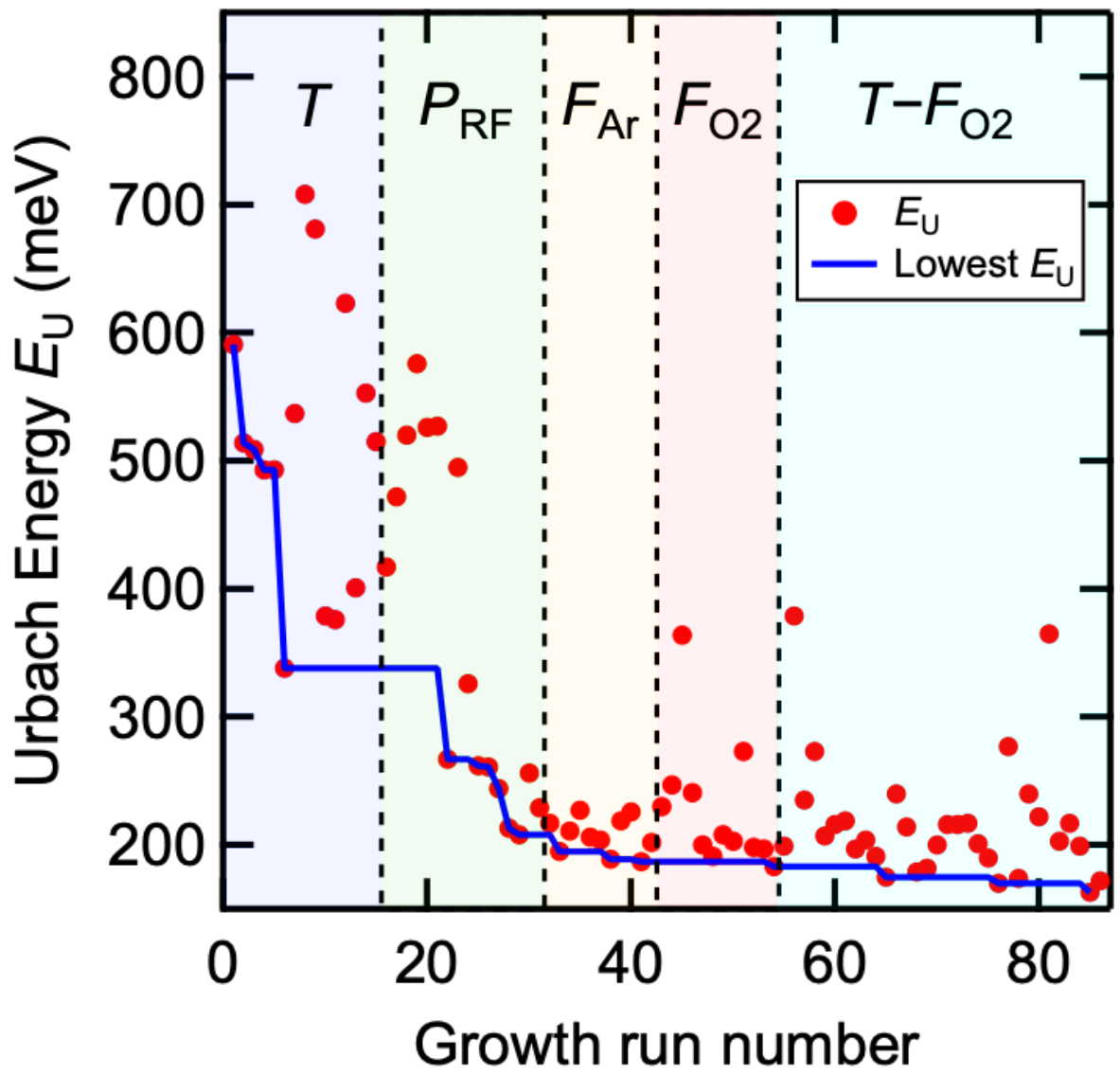

Fig. R4. Human-executed re-optimization following the extracted optimization strategy. A human experimenter performed sequential 1D optimization of T, P_{RF}, F_{Ar}, and F_{O2}, followed by 2D fine-tuning in the T-O_2 plane. The blue, green, yellow, red, and cyan shaded regions indicate the optimization stages for T, P_{RF}, F_{Ar}, F_{O2}, and T-O_2, respectively. Red circles represent the experimental E_U as a function of growth run number. The blue line indicates the lowest E_U achieved up to each run.

Comment 3:

3) Generalization/transfer claims are not strong enough to justify publication as a methodological advance.

The "transferability" demonstrated is narrow (heteroepitaxy window applied to homoepitaxy) and still within the same material system and tool. There is no convincing

evidence that the derived rules are robust across chambers, targets, thickness ranges, substrate miscut/polarity, or broader process windows. Additionally, the surrogate interpretability is trained on only 66 BO-selected samples (non-uniform sampling), which limits confidence that the reported additivity/interaction structure is stable rather than an artifact of the acquisition path. Overall, the manuscript does not establish broadly reusable methodology beyond what is already standard practice in SDL/BO literature, nor does it deliver a clear new scientific result commensurate with a high-impact publication.

Response:

We thank the reviewer for this important and critical comment. As the reviewer pointed out, the original 66-run dataset was acquired through a BO trajectory and was therefore non-uniformly sampled. We also agree that such a dataset alone cannot establish universal robustness of the extracted rules across different sputtering chambers, targets, thickness ranges, substrate miscuts/polarities, or substantially broader process windows. We have therefore revised the manuscript to avoid presenting the extracted growth window as a universally transferable recipe. Instead, we now distinguish between two levels of generalization: (i) the methodological generality of the workflow for extracting and validating human-usable growth rules, and (ii) the empirical transferability of the identified low-E_U growth window to β-Ga_2O_3 homoepitaxy. To strengthen these two points, we performed additional experiments, expanded analyses, and corresponding revisions to the manuscript, as detailed below.

To directly address the concern that the additivity/interaction structure might be an artifact of the initial BO acquisition path, we substantially expanded the experimental dataset from 66 to 215 runs by adding broader sampling of the growth-parameter space and rule-guided re-optimization experiments. This expanded dataset reduces the dependence on the original BO trajectory, as visualized in Fig. R5, which shows how the expanded sampling is distributed across the explored parameter space, and provides a denser basis for response-surface analysis. With the expanded 215-run dataset, the random-forest surrogate achieved an in-sample R^2 of 0.96 and, more importantly, the 5-fold cross-validated R^2 improved substantially to 0.65. Importantly, the central conclusion of the explainable analysis was retained in the expanded dataset. The 215-sample analysis again showed that the E_U landscape is described largely by additive single-parameter contributions with a

residual T–O_2 interaction. In the expanded dataset, the additive-only model reproduced the random-forest predictions only partially (R^2 = 0.72), whereas including a single T–O_2 interaction term markedly improved the approximation (R^2 = 0.85). Consistently, the Friedman H^2 interaction analysis identified the T–O_2 pair as the dominant interaction (H^2 = 0.294), while the other pairwise interactions were substantially smaller (≤ 0.063). Thus, the qualitative structure originally extracted from the 66-run BO dataset—predominantly additive parameter contributions with a residual T–O_2 coupling—is preserved after a more than threefold expansion of the dataset. These results support that the extracted rule is not simply an artifact of the original BO-biased sampling path. We have described this point in the revised manuscript (page 13, line 27).

We further tested whether the extracted rule is robust as an experimentally actionable optimization strategy. As described in our response to Comment 2, the rule distilled from the original dataset suggested a human-executable strategy consisting of sequential one-dimensional tuning of the individual growth parameters, followed by focused two-dimensional refinement in the T–O_2 plane. A human experimenter independently executed this strategy. This experiment reproduced the low-E_U region identified by BO, reached the original BO optimum level of $E_U \leq 182$ meV, and finally achieved E_U = 163 meV, lower than the original BO optimum. This result shows that the extracted rule is not merely a descriptive correlation in the BO dataset, but a practically executable strategy that can guide further optimization within the explored process space. We have described this point in the revised manuscript (page 12, line 8).

Furthermore, to strengthen the transferability claim to homoepitaxy, we also added controlled homoepitaxial growth experiments under multiple conditions. Specifically, we compared homoepitaxial films grown under two independently identified low-E_U conditions on C-plane Al_2O_3: the BO-identified condition, (T, P_{RF}, F_{Ar}, $F_{\mathrm{O_2}}$) = (507°C, 119 W, 26 sccm, 1 sccm), which yielded E_U = 182 meV, and the human-reoptimized condition following the extracted optimization strategy, (562°C, 97 W, 26.5 sccm, 1 sccm), which yielded E_U = 163 meV. Although these two conditions differ in T and P_{RF}, both lie within the same low-E_U window identified from heteroepitaxial growth. XRD θ–2θ scans showed that homoepitaxial films grown under both low-E_U conditions exhibited sharp β-Ga_2O_3 (020) reflections that were indistinguishable from the substrate peak (Fig. R6), indicating high-quality homoepitaxial growth. In contrast, a homoepitaxial film grown

under an off-window condition, (612°C, 97 W, 26.5 sccm, 0 sccm), which corresponded to a much larger E_U = 365 meV on C-plane Al_2O_3, exhibited a broad β-Ga_2O_3 (020) peak (Fig. R6), indicating degraded crystalline quality. These results demonstrate a correspondence between the heteroepitaxial E_U landscape and the crystalline quality of homoepitaxial films: low-E_U conditions on C-plane Al_2O_3 yield high-quality homoepitaxial films, whereas an off-window high-E_U condition leads to degraded homoepitaxial growth, supporting the cross-substrate transferability of the identified low-E_U window within the tested conditions. We have described this point in the revised manuscript [(page 3, lines 13), (page 7, lines 24), (page 13, lines 8), and (page 16, lines 20)].

Taken together, these additional experiments and expanded analyses justify the two revised claims: the workflow can extract and validate human-usable growth rules, and the identified low-E_U window shows empirical cross-substrate transferability to β-Ga_2O_3 homoepitaxy within the tested scope.

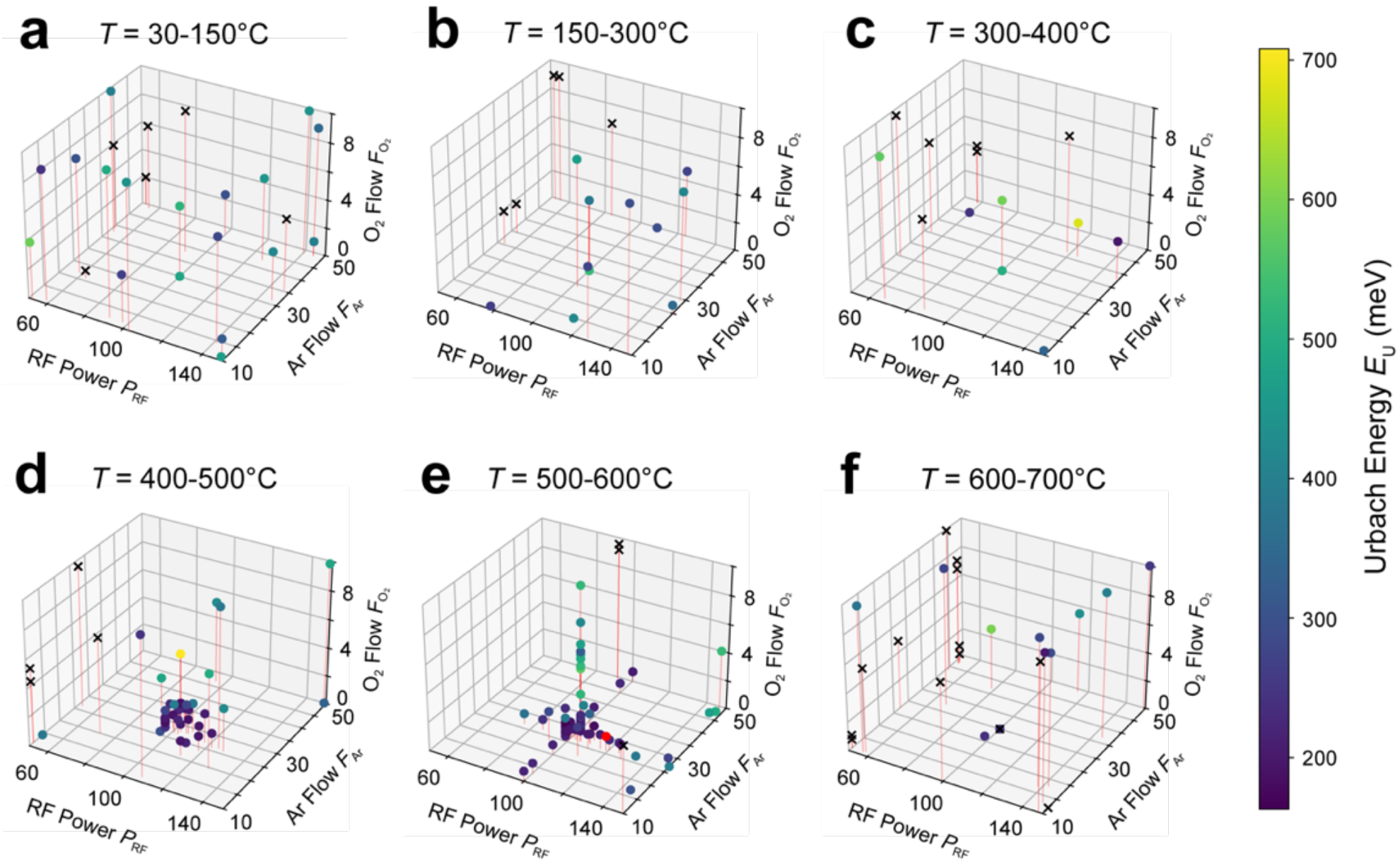

Fig. R5. Experimental E_U distribution in the expanded 215-sample dataset. The experimental E_U in the three-dimensional parameter space P_{RF}-F_{Ar}-F_{O_2} for different T windows of **a** 30-150°C, **b** 150-300°C, **c** 300-400°C, **d** 400-500°C, **e** 500-600°C, and **f** 600-700°C. Marker color represents E_U (color bar, meV), visualizing the distribution of the BO-acquired, additional sampling, and rule-guided re-optimization data. In **e**, the red circle represents the lowest E_U (163 meV) obtained in the rule-guided re-optimization. Black crosses indicate runs for which the E_U could not be extracted (NaN).

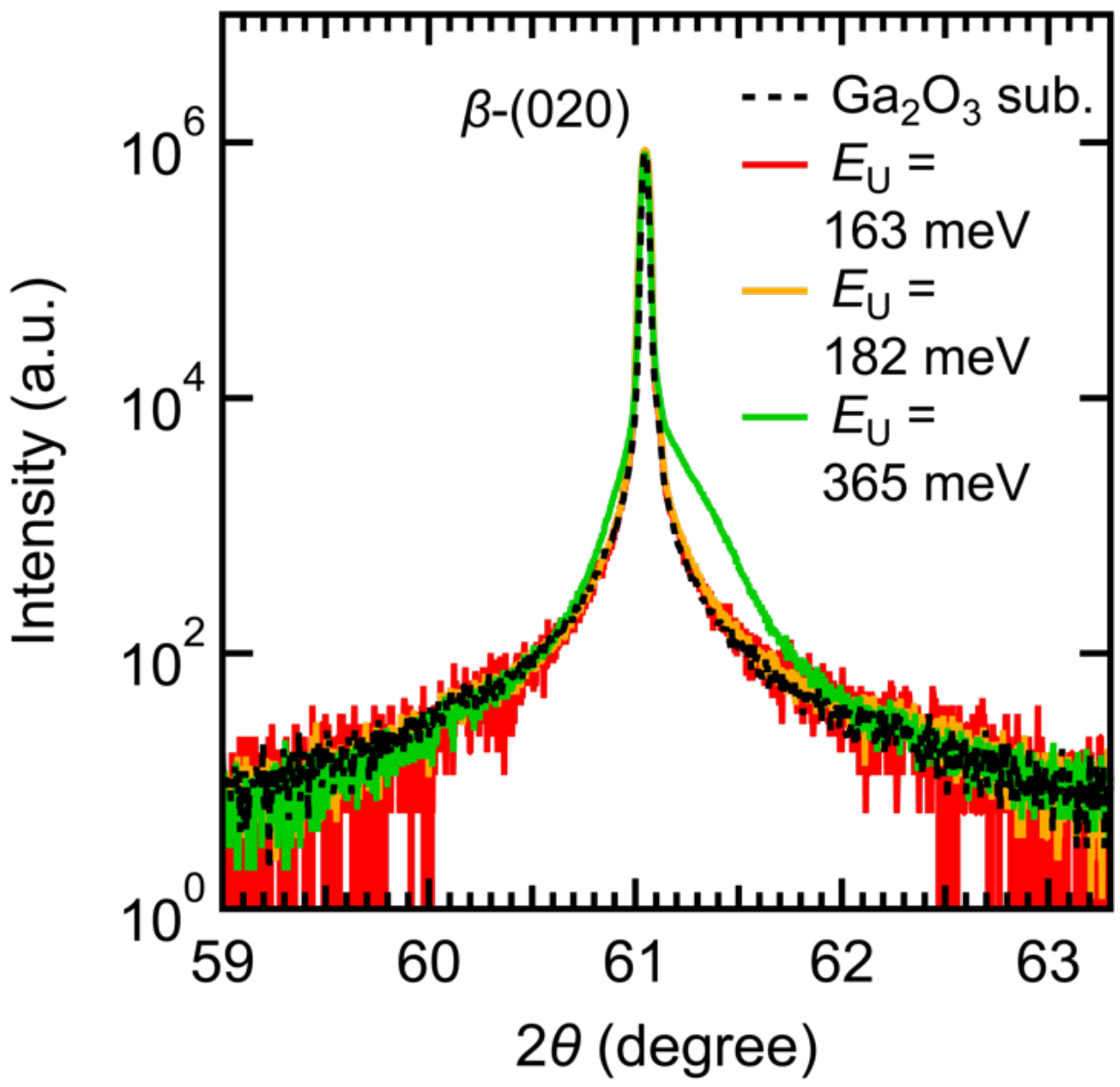

Fig. R6. XRD characterization of *β*-Ga_2O_3 homoepitaxial films. XRD θ-2θ scans of the *β*-(020) reflection for the *β*-Ga_2O_3 homoepitaxial films grown under low-E_U (163 and 182 meV) and high-E_U (365 meV) conditions, together with the substrate.

Response to the comments of the reviewer #3

Comment 1:

I suggest - Major revision.

The manuscript reports a closed-loop sputter epitaxy workflow for β-Ga_2O_3 that couples Bayesian optimization (BO) with automated optical analysis (Urbach energy, EU) and post hoc surrogate-based interpretability. The experimental results appear technically plausible and the study has potential significance for autonomous thin-film process development. However, several framing and transferability claims are currently overstated or insufficiently supported, and key experimental controls/metadata required for reproducibility and for validating the optical metric across a wide process space are not yet documented at the level expected for Nature Communications.

Response:

We sincerely thank the reviewer for the positive evaluation of the technical plausibility and potential significance of our study, as well as for the constructive comments. In response, we have improved the transparency, reproducibility, and validity of the study by adding substantial new experimental data and analyses, and we address each of the reviewer's specific comments in detail below.

Comment 2:

1) Noteworthy results

1. Closed-loop optimization in a 4D sputter parameter space (substrate temperature, RF power, Ar flow, O_2 flow) achieving a low-disorder optical metric (EU ~182 meV) on sapphire within a moderate campaign size (tens of runs).

2. Integration of interpretability through surrogate decomposition (e.g., partial dependence/feature importance/interaction analyses) to propose a human-usable "growth window," rather than reporting only the final optimum.

3. Attempted cross-substrate transfer from heteroepitaxy on sapphire to homoepitaxy on β-Ga_2O_3 substrates using the identified window, positioning the workflow as a practical route to high-quality epitaxial films.

These are meaningful technical outputs that could be valuable to both the sputter epitaxy

community and the broader self-driving laboratory (SDL)/autonomous process optimization community.

2) Significance to the field and positioning vs literature

The work is relevant to two active areas: (i) accelerated discovery/optimization of growth conditions for complex oxides and wide-bandgap semiconductors, and (ii) closed-loop experimentation for materials synthesis and processing. The manuscript is likely of interest to researchers in thin film growth (sputtering/oxide epitaxy), autonomous experimentation, and data-driven process control.

Response:

We thank the reviewer for clearly summarizing the noteworthy aspects, potential impact, and positioning of our work. We appreciate the reviewer's recognition of the relevance of our study to both sputter epitaxy and self-driving laboratory/autonomous process optimization communities. The reviewer's insightful comments helped us further clarify and strengthen the scientific basis and literature positioning of our contributions.

Comment 3:

That said, the manuscript's broader framing around "SDLs as black boxes" should be tightened. Many SDL efforts incorporate physics-informed modeling, constrained/structured BO, and interpretability-by-design. The authors should more precisely locate the "black-box" issue in the decision/optimization layer (and/or lack of distilled transferable rules), rather than implying SDLs as a system concept are intrinsically black-box.

Response:

As the reviewer pointed out, it was not our intention to imply that Self-driving laboratories (SDLs) are intrinsically black-box systems. Rather, our intended point was that, in closed-loop optimization workflows, the objective function $f(\boldsymbol{x})$ optimized by Bayesian optimization (BO) is often treated as a black-box response surface, and the resulting optimization process can yield an optimized recipe without explicitly distilling transferable,

human-usable rules. We have therefore revised the manuscript to more precisely locate the "black-box" issue in the decision/optimization layer and in the lack of post hoc rule distillation, rather than in the SDL concept itself [(page 2, line 6), (page 16, line 9), and (page 17, line 27) in the revised manuscript].

Comment 4:

3) Do the data support the conclusions and claims?

Partially. The dataset and demonstrated improvement in EU support the claim that the closed-loop workflow can efficiently identify high-quality growth conditions within the explored bounds. The interpretability analysis plausibly supports qualitative growth trends (e.g., which parameters matter most within the tested region). However, additional evidence is needed to support two central claims:

(A) "Acceleration/efficiency" beyond run count

The manuscript reports reaching a low EU after a certain number of runs, but does not provide a quantitative baseline comparison (time/experiments avoided) against conventional optimization practice. If "acceleration" is a major motivation, it should be explicitly quantified (e.g., implied size of a comparable grid/OFAT campaign within the same bounds; or a realistic expert-guided workflow) and reported as an acceleration factor or experiment/time reduction to achieve EU $\leq$ a defined threshold.

Response:

We agree with the reviewer that reporting only the run number does not fully quantify the acceleration or efficiency of the closed-loop workflow. In the revised manuscript, we therefore added a more explicit comparison in terms of both experimental cycles and working time. Specifically, the BO campaign presented in the original manuscript reached the original BO minimum E_U of 182 meV by the 56th run, corresponding to approximately 8 working days under the throughput of our SDL workflow (7-8 runs per day). As a practical baseline, we used the human-executable optimization strategy proposed in the original manuscript, which was derived from the interpretable analysis of the random-forest surrogate trained on the N = 66 BO-acquired dataset. This analysis revealed a largely additive structure among the four growth parameters, with the T–O_2 coupling defining the narrow window for high-quality growth. Based on this structure,

we additionally performed sequential 1D optimization in the order of T, P_{RF}, F_{Ar}, and F_{O2}, followed by 2D fine-tuning in the T-O_2 plane around the then-current optimum obtained from the 1D steps (Fig. R7). This human-executable strategy required 65 runs to reach $E_U \leq 182$ meV; under a conventional non-automated workflow with manual sputter growth and optical characterization at approximately 3 runs per day, this would correspond to approximately 22 working days. Thus, the present closed-loop SDL workflow reduces the working time required to reach the same E_U threshold by roughly a factor of 2.7. We have added this quantitative comparison to clarify the practical efficiency advantage of the proposed workflow, while noting that this is a conservative benchmark because the human-executable strategy already benefits from the growth rule distilled from the BO dataset [(page 12, line 25) in the revised manuscript]. Although the continued human-executable optimization eventually reached a lower minimum E_U of 163 meV after 85 runs (Fig. R7), we used the common threshold of $E_U \leq 182$ meV for the efficiency comparison to keep the acceleration metric well defined.

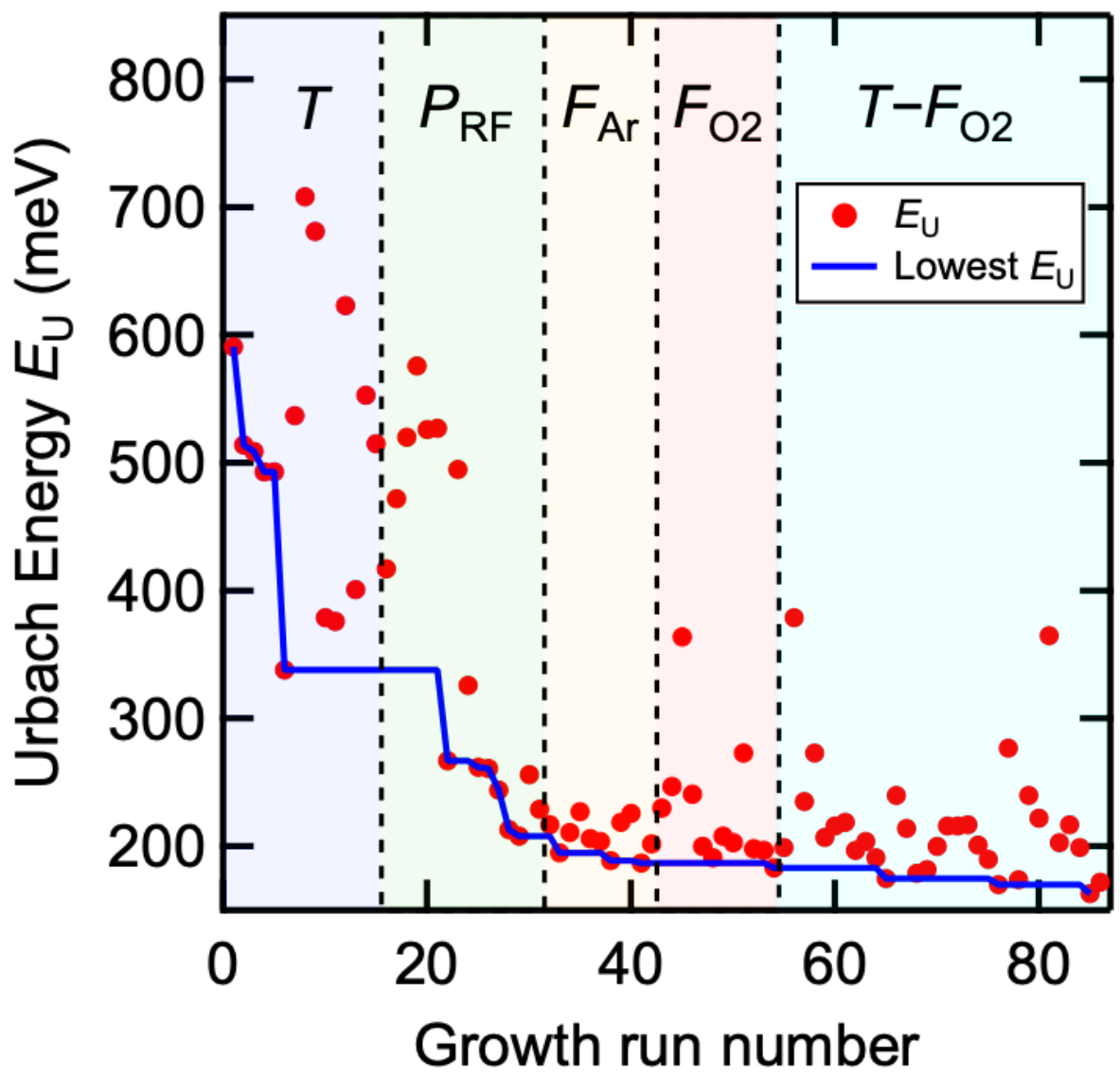

Fig. R7. Human-executed re-optimization following the extracted optimization strategy. A human experimenter performed sequential 1D optimization of T, P_{RF}, F_{Ar}, and F_{O2}, followed by 2D fine-tuning in the T-O_2 plane. The blue, green, yellow, red, and cyan shaded regions indicate the optimization stages for T, P_{RF}, F_{Ar}, F_{O2}, and T-O_2, respectively. Red circles represent the experimental E_U as a function of growth run number. The blue line indicates the lowest E_U achieved up to each run.

Comment 5:

(B) "Transferability" to homoepitaxy

The claim that the BO-identified window is "directly transferable" from sapphire to β-Ga_2O_3 substrates is potentially high-impact but currently under-controlled. Substrate identity is not an input variable in the model; therefore, successful growth on a different substrate does not automatically demonstrate that the learned "rules" are substrate-insensitive or mechanistically transferable. At minimum, the manuscript should include a small comparative set on β-Ga_2O_3 (e.g., perturbations around the claimed optimum in temperature and O_2 flow and/or one other knob) to demonstrate the robustness and local optimality of the window on the new substrate. Alternatively, the authors should soften the claim to an empirical observation ("the identified window was sufficient to enable homoepitaxy under our tool conditions") rather than a general statement of transferability. (Personal opinion is that the transferability is from the workflow Not from the conditions/windows).

Response:

We thank the reviewer for this important comment. To examine the transferability of the learned growth rules for homoepitaxial growth on β-Ga_2O_3 substrates, we have added a small comparative set of additional homoepitaxial growth experiments and revised the wording to more precisely describe the scope of the transferability claim. Specifically, we examined homoepitaxial films grown under two independently identified conditions within the same low-E_U window on C-plane Al_2O_3: the BO-identified condition, (T, P_{RF}, F_{Ar}, F_{O_2}) = (507°C, 119 W, 26 sccm, 1 sccm), which yielded E_U = 182 meV, and the human-reoptimized condition following the extracted optimization strategy, (562°C, 97 W, 26.5 sccm, 1 sccm), which yielded E_U = 163 meV. Although these two conditions differ in T and P_{RF}, they lie within the same low-E_U window identified from the heteroepitaxial optimization, with similar F_{Ar} and F_{O_2} values. XRD θ-2θ scans show that the β-Ga_2O_3 (020) peaks of both films are indistinguishable from the substrate peak, indicating highly coherent homoepitaxial growth under both low-E_U conditions (Fig. R8), consistent with the high-quality homoepitaxy observed by STEM (Fig. 4 in the original manuscript). In contrast, for a homoepitaxial film grown under an off-window condition, (612°C, 97 W,

26.5 sccm, 0 sccm), which corresponded to a much larger E_U = 365 meV on C-plane Al_2O_3, a broad β-Ga_2O_3 (020) peak was observed (Fig. R8). These additional results show a correspondence between the quality of β-Ga_2O_3 homoepitaxial films and the E_U landscape established from heteroepitaxy on C-plane Al_2O_3: low-E_U conditions yield high-quality homoepitaxial films, whereas an off-window high-E_U condition leads to degraded crystalline quality. We therefore revised the manuscript to state: "These results show that the identified low-E_U window was sufficient to enable high-quality β-Ga_2O_3 homoepitaxy under our tool conditions and support the cross-substrate transferability of the growth rules extracted from the BO-acquired dataset." We also agree with the reviewer that the broader methodological implication is the transferability of the workflow for extracting and validating growth rules, rather than universal transferability of specific growth conditions. We have revised the manuscript accordingly to clarify this distinction and to avoid implying universal substrate-independent transferability of a fixed recipe. We have described this point in the revised manuscript [(page 3, lines 13), (page 7, lines 24), (page 13, lines 8), and (page 16, lines 20)].

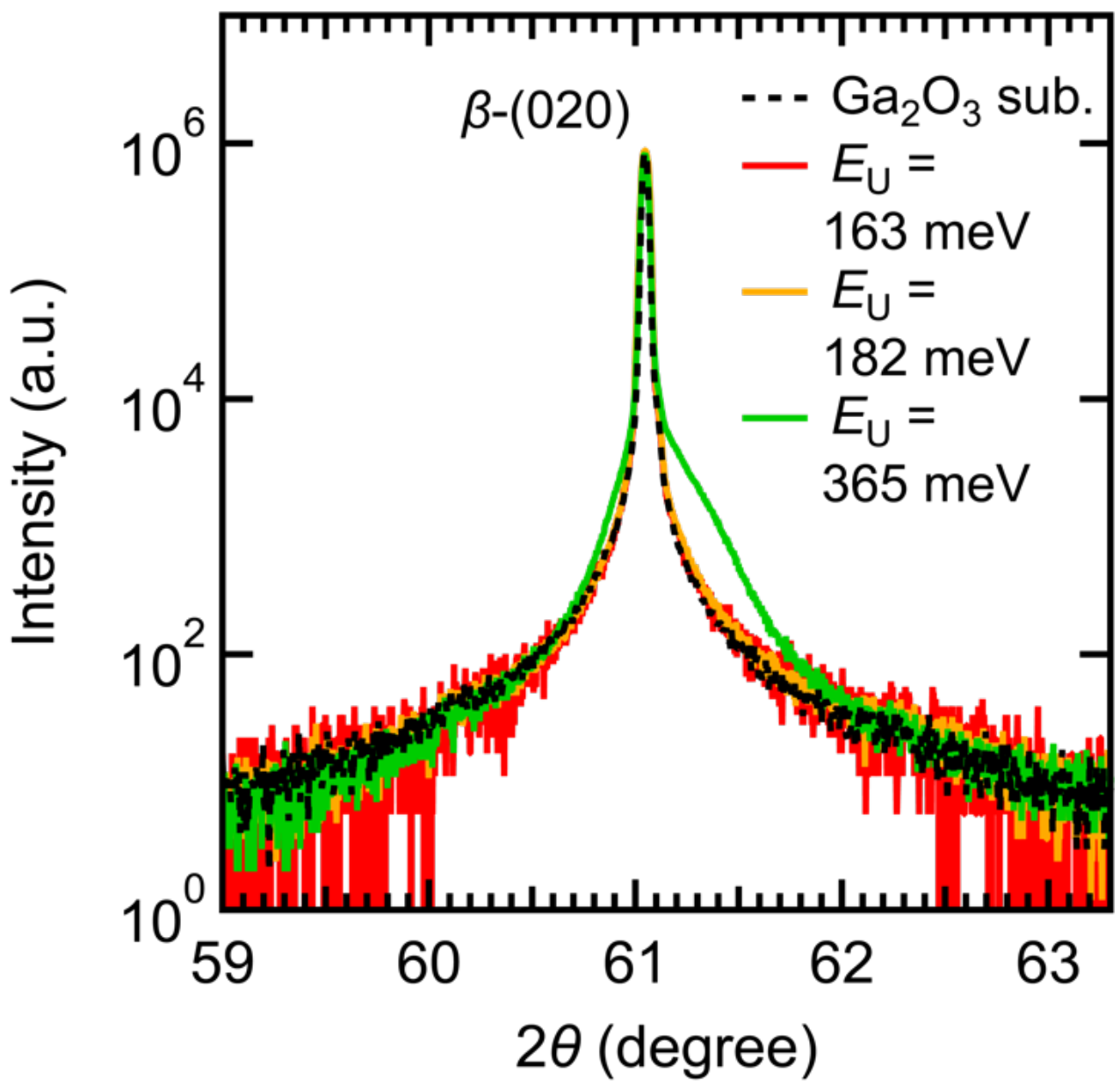

Fig. R8. XRD characterization of β-Ga_2O_3 homoepitaxial films. XRD θ-2θ scans of the β-(020) reflection for the β-Ga_2O_3 homoepitaxial films grown under low-E_U (163 and 182 meV) and high-E_U (365 meV) conditions, together with the substrate.

Comment 6:

4) Concerns about data analysis, interpretation, and conclusions

4.1 Overgeneralized "black-box SDL" statements

The manuscript would benefit from a careful rewrite of the SDL discussion. SDLs include automation, sensing, data infrastructure, and decision-making; the "black-box" critique is more appropriately applied to the BO/surrogate decision layer and to the lack of interpretable, transferable process knowledge in many reports. This distinction matters for technical accuracy and for fair positioning of the contribution.

Response:

As the reviewer pointed out, it was not our intention to imply that SDLs are intrinsically black-box systems. We agree that SDLs comprise multiple components, including automation, sensing, data infrastructure, and decision-making. Rather, our intended point was that, in many closed-loop optimization workflows, the BO/surrogate-based decision layer treats the process–property relationship $f(\mathbf{x})$ as a black-box response surface and can identify an optimized recipe without explicitly distilling transferable, human-usable process knowledge. We have therefore revised the relevant parts of the Introduction and Discussion to make this distinction clear and to avoid implying that SDLs as integrated experimental systems are intrinsically black-box [(page 2, line 6), (page 16, line 9), and (page 17, line 27) in the revised manuscript].

Comment 7:

4.2 Optical metric robustness vs thickness/optics artifacts

EU is extracted from optical data, and the manuscript indicates a nominal constant thickness (e.g., 55 nm) using pre-calibrated deposition rate to set growth time. But deposition rate can change with RF power and gas composition/flows. If thickness varies significantly across the BO campaign, EU extraction and cross-run comparability may be biased (e.g., interference fringes, absorption path length changes, or analysis assumptions). The authors should:

Provide thickness measurements for a representative subset across the explored parameter space (or ideally all runs) using profilometry/ellipsometry/XRR/cross-sectional microscopy;

Quantify thickness variation and evaluate whether EU correlates with thickness;
Clarify whether "55 nm" is measured or nominal, and how uncertainties propagate into EU.

Response:

We thank the reviewer for raising this important point. In the revised manuscript, we have clarified that the "55 nm" value denotes a target nominal thickness, rather than an independently measured thickness for every run [(page 18, line 17) in the revised manuscript]. Specifically, the deposition time was determined for each run so as to target a nominal thickness of 55 nm, using pre-calibrated deposition-rate data measured at room temperature. Table R3 summarizes the deposition rates calculated from the film thicknesses measured by a stylus profiler after deposition under various P_{RF}, F_{Ar}, and F_{O2} conditions. At P_{RF} = 100 W, the deposition rate does not depend strongly on F_{Ar} or F_{O2} and remains in the range of 0.80-0.89 nm/min. By contrast, as P_{RF} increases from 60 to 130 W, the deposition rate increases systematically from 0.64 to 1.42 nm/min. Therefore, we estimated the deposition rate from an empirical quadratic fit to the rate-versus-P_{RF} calibration curve and determined the growth time accordingly for each run (Fig. R9). In addition, regarding the effect of T, the actual thickness of the film grown under the growth condition (T, P_{RF}, F_{Ar}, F_{O_2}) = (507°C, 119 W, 26 sccm, 1 sccm) was estimated from STEM observations (Fig. 4 in the revised manuscript) to be 56 ± 2 nm, which is in good agreement with the target nominal thickness of 55 nm. This agreement suggests that, at least near the optimized high-quality growth window, temperature-dependent changes in the effective deposition rate do not significantly affect the nominal thickness control. We have described these points in the revised manuscript (page 18, line 17) and in the revised Supplementary Information (page 3, line 1).

We further note that the extracted E_U is intrinsically much less sensitive to a thickness error than the absolute absorption coefficient $\alpha(h\nu)$ as a function of photon energy $h\nu$. As described in the original manuscript, in our automated optical analysis, the absorption coefficient was estimated from the measured transmittance using the Beer–Lambert relation, and E_U was then extracted from the slope of $\ln[\alpha(h\nu)]$ in the Urbach-tail region. Therefore, a constant error in the assumed film thickness mainly produces a vertical offset in $\ln[\alpha(h\nu)]$, while leaving the fitted slope, and hence E_U, unchanged in principle. To experimentally verify that the E_U values used in this study are not dominated by thickness

variation, we additionally grew a thinner film under a representative low-E_U condition. For the condition (520°C, 97 W, 26.5 sccm, 0.4 sccm), the nominal 55-nm film yielded E_U = 216 meV. When the deposition time was intentionally reduced to obtain a 10% thinner film with a nominal thickness of 50 nm, the extracted E_U was 220 meV, which is close to the value obtained for the nominal 55-nm film. This result supports that a thickness difference of this magnitude does not significantly alter the extracted E_U. We have described this point in the revised manuscript (page 20, line 8).

P_{RF} (W)	F_{Ar} (sccm)	F_{O_2} (sccm)	Rate (nm/min)
60	9	0	0.64
80	9	0	0.72
100	6	0	0.84
100	9	0	0.89
100	12	0	0.86
100	30	0	0.83
100	30	1	0.8
100	30	5	0.81
119	26	1	1.18
130	30	0	1.42

Table R3. Pre-calibrated deposition-rate data used for nominal thickness control. The table summarizes the deposition rates estimated from film thicknesses measured by a stylus profiler after deposition under representative P_{RF}, F_{Ar}, and F_{O2} conditions.

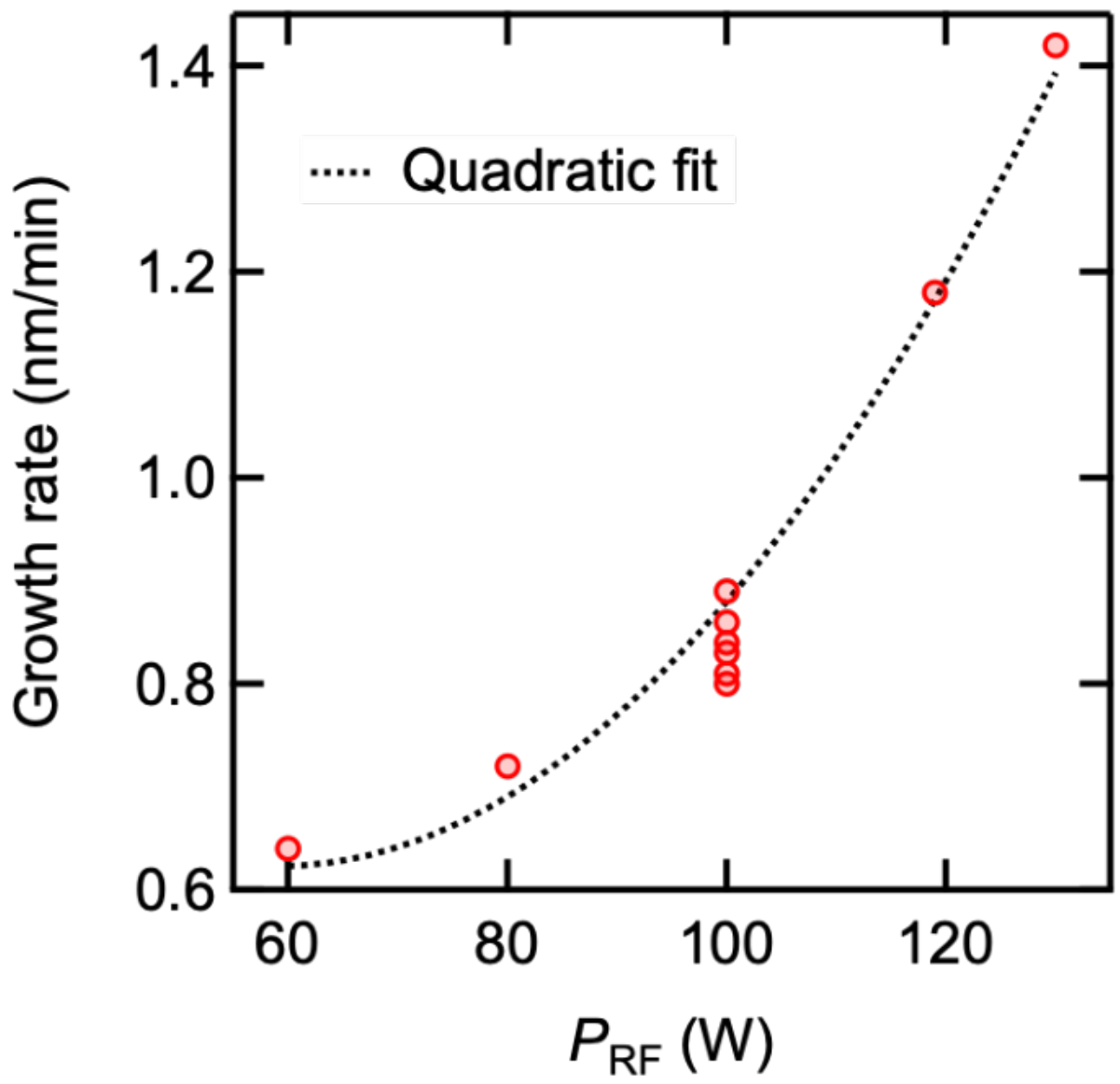

Fig. R9. Deposition-rate calibration used for nominal thickness control. Deposition rate as a function of P_{RF}, using the data in Table R3. The solid curve is the empirical quadratic fit.

Comment 8:

4.3 Interpretability vs “physics-informed” wording

The interpretability in this manuscript appears to come from post hoc surrogate interrogation (e.g., random forest feature importance and PDP analyses). That is valuable, but it is distinct from “physics-informed ML” (constraints/priors/structure embedded in the model). The authors should clearly state what is physics-informed (if anything) versus what is post hoc interpretability, and avoid conflating the two.

Response:

As the reviewer pointed out, in the BO algorithm used in this study, we did not adopt a physics-informed approach in which physics knowledge is embedded into the surrogate model or acquisition function in the form of constraints, priors, kernels, or other model structures. Instead, we treated the objective function $f(\boldsymbol{x})$ as a black-box response surface and obtained human-interpretable knowledge by evaluating the behavior of $f(\boldsymbol{x})$ purely through data-driven machine-learning predictions. We have clarified this point in the revised manuscript [(page 2, line 6), (page 16, line 9), and (page 17, line 27)]. As a future direction, it would also be possible to combine physics-informed machine learning

approaches with the post hoc interpretability used in this study. For example, one could embed prior physical knowledge into the model and then treat the discrepancy between the experimentally observed data and the physics-informed model as a black-box function, which could subsequently be optimized and analyzed using post hoc interpretability.

Comment 9:

5) Methodology soundness and expected standards

The overall methodology—closed-loop BO with an automated objective and subsequent interpretability analysis—is sound and aligned with current best practices. The experiment design and the multi-parameter search are appropriate. However, the manuscript should strengthen: Uncertainty quantification (replicate measurements and run-to-run variability, especially near the optimum); Objective validity (EU extraction details and robustness checks); Comparability controls (thickness verification, instrument calibration stability, and handling of measurement noise/outliers). we require revision to meet the standard of evidence for broad claims (efficiency + transferability).

Response:

We thank the reviewer for this constructive summary of the methodological issues that should be strengthened. As described below, we have revised the manuscript to address these points by strengthening uncertainty quantification, objective validity, comparability controls, and the evidence supporting the efficiency and transferability claims.

For uncertainty quantification, we added replicate growth experiments at representative conditions, including the BO-identified optimum, nearby low-E_U conditions, and an off-optimal high-E_U condition, as described in our response to Reviewer #1, Comment 3 [(page 2, line 20) in this response letter]. These experiments demonstrate small run-to-run variability near the optimum and confirm that this variability is much smaller than the systematic variation in E_U across the growth-parameter space.

For objective validity, we expanded the description of the automated E_U extraction procedure in the revised manuscript, and the corresponding analysis code is available via the Code availability section. The extracted E_U values were confirmed to be stable against reasonable changes in the fitting window and fitting criteria, supporting that the observed trends are not artifacts of a particular fitting choice.

For comparability controls, we clarified that 55 nm is a target nominal thickness, added deposition-rate calibration data, and included a thickness-control experiment in which a 10% thinner film yielded a similar E_U value, as described in our response to Comment 7 [(page 26, line 22) in this response letter]. To ensure cross-run comparability of the optical measurements, we also paid careful attention to instrument calibration stability: a C-plane Al_2O_3 substrate was measured as a daily reference to minimize artifacts originating from the measurement system and possible effects of temporal drift.

For noise and outlier handling, the automated fitting procedure used strict predefined quality criteria. In the analysis code, both the Tauc fit for band gap energy (Eg) and the Urbach-tail fit for E_U were required to satisfy a stringent fitting-quality criterion of $R^2 \geq 0.995$. The representative extraction shown in Fig. R10 demonstrates that both Eg and E_U are obtained with high fitting accuracy. Regarding the outlier handling, spectra for samples that did not correspond to successful β-Ga_2O_3 film growth, identified by a transmittance below 0.8 at 500 nm, were treated as failed/NaN evaluations rather than valid objective values. We have clarified this point in the revised manuscript (page 20, line 19). The treatment of NaN evaluations in the BO workflow is described in detail in the Methods section [(page 21, line 10) in the revised manuscript]. These procedures prevent noisy, ill-defined, or physically invalid spectra from biasing the optimization or the subsequent interpretability analysis.

For efficiency and transferability, we added additional analyses and experiments as described in our responses to Comments 4 [(page 22, line 6) in this response letter] and 5 [(page 24, line 2) in this response letter], respectively. For efficiency, we quantified the experimental cost in terms of both run count and working time, showing that the original BO campaign reached E_U = 182 meV by the 56th run, corresponding to approximately 8 working days with the SDL workflow, whereas the human-executable sequential optimization would require approximately 22 working days under a conventional non-automated workflow. For transferability, we added controlled homoepitaxial growth experiments showing that low-E_U conditions identified on C-plane Al_2O_3 yield high-quality β-Ga_2O_3 homoepitaxial films, whereas an off-window high-E_U condition leads to degraded crystalline quality.

Taken together, these revisions strengthen the methodological rigor of the study and the experimental support for the central claims. We appreciate the reviewer's

constructive comments, which helped us improve the manuscript to better meet the standards of Nature Communications.

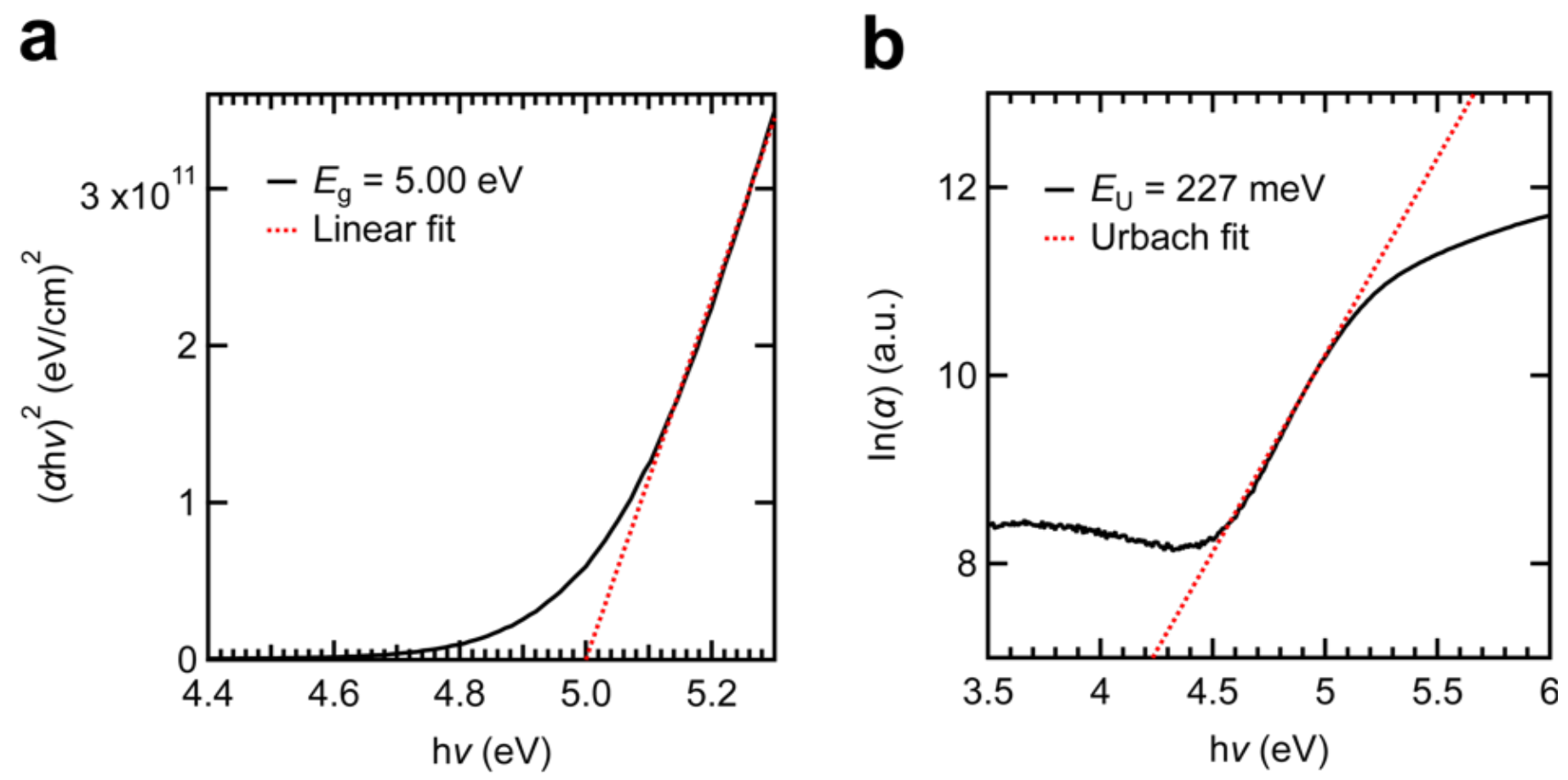

Fig. R10. Representative procedure for optical extraction of E_g and E_U of the β-Ga_2O_3 film with E_g = 5.00 eV and E_U = 227 meV on C-plane Al_2O_3. a Tauc plot $(\alpha h\nu)^2$ as a function of photon energy $h\nu$, with a linear fit used to estimate E_g. **b** Urbach energy analysis shown as $\ln(\alpha)$ versus $h\nu$ with a linear fit in the exponential tail region used to extract E_U.

Comment 10:

(Remarks on code availability)

Supporting code is provided with the submission; however, it is relatively general and does not substantially impact my evaluation of the experimental results or the main claims.

Response:

We thank the reviewer for checking the supporting code. The code was provided to ensure transparency and reproducibility of the data analysis and optimization workflow.

Response to the comments of the reviewer #4

Comment 1:

Authors demonstrate an SDL that optimizes the growth of very high quality (as measured by the Urbach energy) β-Ga2O3 films, achieving an E_U as low as ~180 meV. Authors also show that the optimal growth conditions for heteroepitaxial growth can be transferred to homoepitaxial conditions. These achievements are notable because the films are grown by RF sputtering, which is a much more scalable method compared to conventional CVD or MBE.

Overall, I find this manuscript to be of high quality, and commend the authors for its clarity. However, there are a few areas which could be improved.

Response:

We thank the reviewer for the positive evaluation of our manuscript and for recognizing the significance of achieving β-Ga_2O_3 films with low Urbach energy (E_U) by RF sputtering, as well as the transfer of the optimized growth conditions to homoepitaxial growth. We also appreciate the reviewer's constructive suggestions. In response to these comments, we have carried out additional experiments and analyses, which have substantially improved the robustness, clarity, and overall quality of the manuscript. Our point-by-point responses are provided below.

Comment 2:

1. Authors repeatedly state that BO models are "black boxes" to support their explainability work. This is misleading. Both RFS and GPs can be used in BO algorithms, and both ML algorithms are explainable: RFs through impurity, Gini, or SHAP scores; and GPs through lengthscales. In fact, the authors perform both these analyses in the manuscript.

Response:

We agree that it is inaccurate to imply that BO models or the surrogate models used in BO are intrinsically unexplainable. As the reviewer notes, both random forests and Gaussian processes can provide useful information: random forests can be analyzed through feature importance, impurity-based scores, while Gaussian processes can provide insight

through kernel length scales and predictive uncertainty. Our intended point was that, in closed-loop optimization workflows, the objective function $f(\boldsymbol{x})$ optimized by Bayesian optimization (BO) is often treated as a black-box response surface, and the resulting optimization process can yield an optimized recipe without explicitly distilling human-usable rules and optimization strategy. We have therefore revised the manuscript to clarify this distinction. Specifically, we now locate the "black-box" issue in the decision/optimization layer and in the absence of post hoc rule distillation, rather than in BO or Self-driving laboratories (SDLs) as a whole [(page 2, line 6), (page 16, line 9), and (page 17, line 27) in the revised manuscript]. In the revised manuscript, our contribution is framed as using the closed-loop BO dataset and explainable surrogate analyses to distill the optimized response surface into human-usable growth rules and a practical optimization strategy [(page 15, line 6) in the revised manuscript].

Comment 3:

2. Authors state that their approach constitutes "interpretable" ML. This is inaccurate. Interpretable models are those that are inherently understandable (e.g., linear regression, decision trees). These statements should be changed to "explainable" ML: post hoc analysis of models.

Response:

We thank the reviewer for this helpful clarification. We agree that "interpretable ML" is not the most accurate terminology for the present analysis, because the surrogate models used in this study are analyzed post hoc rather than being inherently interpretable models. In this study, post hoc explainable ML analyses of the closed-loop dataset were used to derive human-interpretable growth rules and a practical optimization strategy. We have therefore revised the manuscript to replace "interpretable ML" with "explainable ML" where appropriate. We now use "explainable" to describe the post hoc surrogate-model analyses and reserve "interpretable" for the distilled growth rules and human-usable optimization strategy. We have described these points in the revised manuscript [(page 5, line 26), (page 8, line 26), (page 22, line 1), (page 24, line 8), and (page 28, line 2)].

Comment 4:

3. I think that the transferability of growth conditions from heteroepitaxial to homoepitaxial should be explained more thoroughly, especially for a paper in a more generalist journal like Nat Comms. How likely is it that the transferability is simply coincidental, rather than the BO algorithm finding the best growth conditions regardless of substrate?

Response:

We thank the reviewer for raising this important point. To examine whether the observed transferability was merely coincidental, we carried out additional homoepitaxial growth experiments under multiple conditions, including both low-E_U conditions and an off-window high-E_U condition identified from heteroepitaxial growth on C-plane Al_2O_3. Specifically, we compared homoepitaxial films grown under two independently identified low-E_U conditions on C-plane Al_2O_3: the BO-identified condition, (T, P_{RF}, F_{Ar}, F_{O_2}) = (507°C, 119 W, 26 sccm, 1 sccm), which yielded E_U = 182 meV, and the human-reoptimized condition following the extracted optimization strategy, (562°C, 97 W, 26.5 sccm, 1 sccm), which yielded E_U = 163 meV. Although these two conditions differ in T and P_{RF}, they lie within the same low-E_U window identified from the heteroepitaxial optimization. XRD θ–2θ scans show that both films exhibit sharp β-Ga_2O_3 (020) reflections that are indistinguishable from the substrate peak, indicating high-quality homoepitaxial growth (Fig. R11). In contrast, a homoepitaxial film grown under an off-window condition, (612°C, 97 W, 26.5 sccm, 0 sccm), which corresponded to a much larger E_U = 365 meV on C-plane Al_2O_3, exhibited a broad β-Ga_2O_3 (020) peak, indicating degraded crystalline quality. These results suggest that the transfer to homoepitaxy is not simply a single coincidental success at one optimized recipe. Instead, they show a correspondence between the E_U landscape established from heteroepitaxial growth and the crystalline quality of homoepitaxial films: low-E_U conditions on C-plane Al_2O_3 yield high-quality homoepitaxial films, whereas an off-window high-E_U condition leads to degraded homoepitaxial growth. Although these experiments do not establish a one-to-one correspondence between the heteroepitaxial E_U landscape and the homoepitaxial growth outcome over the entire parameter space, the observed low-window/off-window correspondence supports the interpretation that the identified growth window reflects sputtering-process factors, including growth kinetics and oxygen supply, rather than being a recipe specific only to the Al_2O_3 substrate. We have described this point in the revised manuscript [(page 3, lines 13), (page 7, lines 24), (page

13, lines 8), and (page 16, lines 20)].

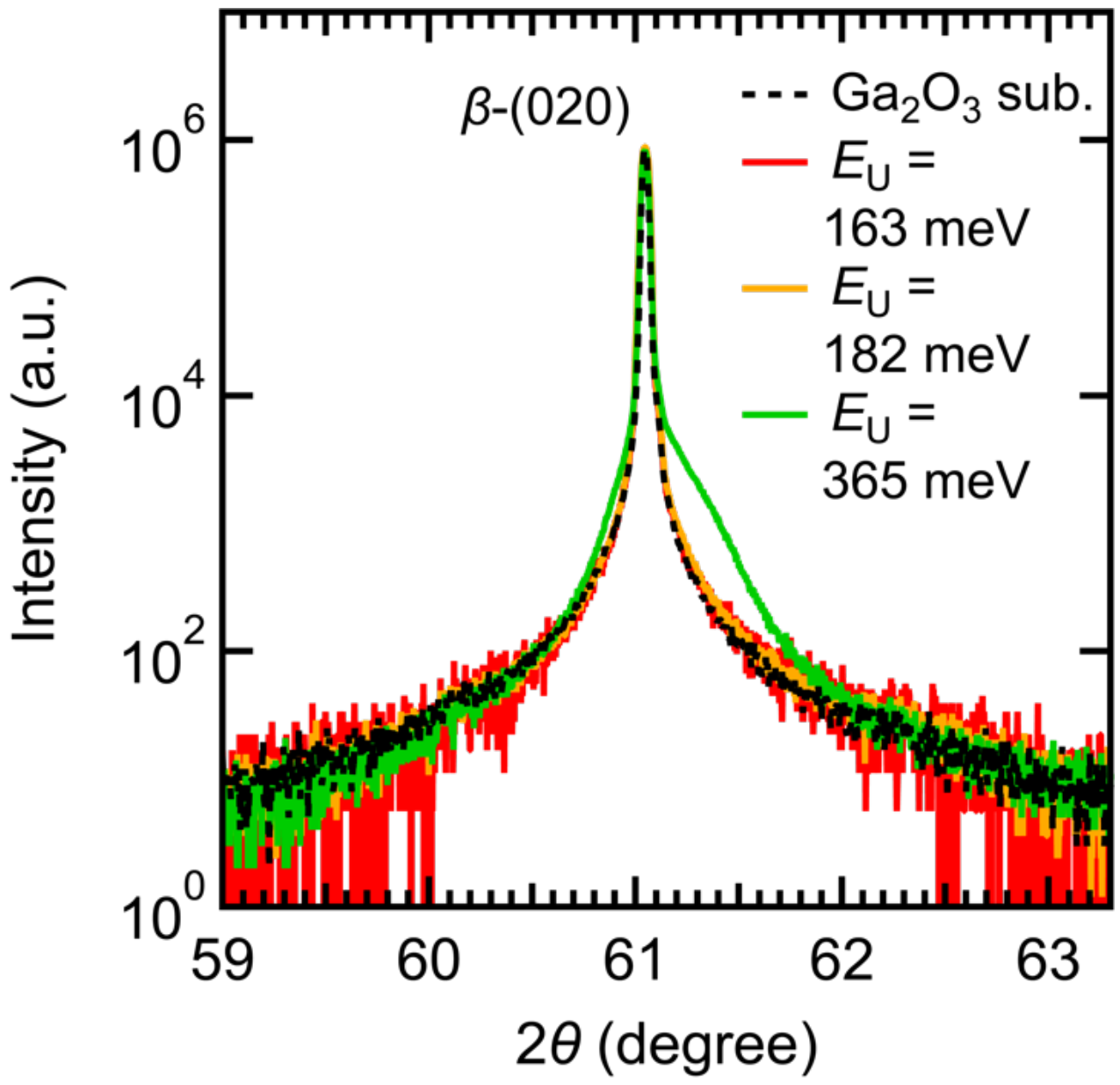

Fig. R11. XRD characterization of *β*-Ga_2O_3 homoepitaxial films. XRD θ-2θ scans of the *β*-(020) reflection for the *β*-Ga_2O_3 homoepitaxial films grown under low-E_U (163 and 182 meV) and high-E_U (365 meV) conditions, together with the substrate.

Comment 5:

4. How novel is the discovery that T is the most important parameter for growth of high-quality β-Ga2O3 films? I believe this is reasonably well-understood across different fields since higher temperatures provide more reorganization energy. Furthermore, the choice to do the first seven runs with fixed P_RF, F_Ar and F_O2 while varying T suggests that this may have been known ahead of time. I would imagine that if the response surface was truly unknown, then choosing a seed dataset by DOE would have been a stronger approach.

Response:

We agree that, based on the general understanding of thin-film and oxide growth, it is natural to expect substrate temperature to be an important parameter, because it affects adatom mobility, surface reorganization, and thermodynamic stability. Importantly, in response to this concern, we substantially expanded the dataset by more than threefold from 66 to 215 runs by adding new experiments designed to reduce the bias associated with the original BO trajectory, including broader sampling of the growth-parameter space and

human-executable re-optimization based on the extracted strategy. This expanded dataset led to a more refined conclusion than the simple expectation that temperature should dominate. The feature importances obtained from the expanded dataset are F_{O_2} = 0.358, P_{RF} = 0.318, T = 0.188, and F_{Ar} = 0.136. Thus, the improved surrogate indicates that high-quality β-Ga_2O_3 sputter growth is not controlled by temperature alone, but by a multi-parameter balance involving oxygen supply and sputtering/plasma conditions. The expanded dataset also improved the 5-fold cross-validated test score of the random-forest surrogate to 0.65, providing a more reliable basis for interpreting the response surface. Importantly, although the feature-importance ranking changed after dataset expansion, the key structural picture obtained from the original 66-run surrogate analysis was preserved in the expanded 215-run dataset: the E_U landscape can still be described by largely additive single-parameter contributions with a residual T–O_2 interaction. Therefore, the key contribution and novelty are not the statement that temperature is important, but the data-driven distillation of the four-dimensional sputtering response surface into human-usable growth rules and an experimentally executable optimization strategy. We note that the initial temperature sweep was chosen as a pragmatic commissioning step to confirm stable operation of the newly constructed sputtering workflow over a broad temperature range, rather than to impose the conclusion that temperature would be the dominant parameter. We have described these points in the revised manuscript (page 13, lines 27) and in the revised Supplementary Information (page 12, line 1).

As the reviewer pointed out, a space-filling DOE seed dataset could be a stronger initialization strategy, particularly when the response surface is completely unknown. A systematic benchmark of different seeding strategies, such as temperature-sweep, DOE-based, and random initialization, would be an important subject for future SDL studies.

Comment 6:

5. I don't fully understand why the authors chose to use a RF model for the explainability section of the paper. A GP can be "explained" just as well as an RF, and the GP is already what's used in the BO algorithm. So explaining what the GP learned would be more informative as to the BO process anyway. Authors justify the selection of the RF because it has a higher in-sample (i.e. training) R2 score (0.92 for RF vs 0.76 for GP). However, the RF's 5-fold CV test score is much work (0.43). This suggests that the RF is overfitting to

the data, which is more likely given that the tree depth hyperparameter is set to unlimited depth... What is the 5-fold CV test score of the GP? It may provide a stronger basis for explainability.

<u>Response:</u>

We thank the reviewer for this important and technically insightful comment. First, following the reviewer's suggestion, we evaluated the 5-fold cross-validated performance of the GP surrogate used in the BO workflow. The GP model yielded a 5-fold CV test R^2 of 0.20 on the original 66-run dataset, which is lower than that of the random-forest surrogate (R^2 = 0.43). Thus, for the original BO-acquired dataset, the GP did not provide a stronger cross-validated predictive basis than the random forest. Accordingly, the GP used for BO was employed as a model for sample-efficient acquisition and uncertainty-guided exploration, while the random forest was used as a post hoc surrogate for decomposing the experimentally sampled response surface and extracting human-executable growth rules. We have described this point in the revised manuscript (page 17, lines 10) and in the revised Supplementary Information (page 9, line 14).

The relatively low CV performance for both models reflects the limited size and non-uniform, BO-biased distribution of the original dataset, rather than a limitation specific to the random-forest analysis. To address this limitation directly, we substantially expanded the experimental dataset from 66 to 215 runs by adding broader sampling and rule-guided re-optimization experiments. This expanded dataset reduced the dependence on the original BO trajectory and provided a denser basis for response-surface analysis, as visualized in Fig. R12, which shows how the expanded sampling is distributed across the explored parameter space. With this expanded dataset, the random-forest surrogate achieved an in-sample R^2 of 0.96 and, more importantly, its 5-fold CV performance improved substantially to R^2 = 0.65. Importantly, this improvement was not a consequence of using unlimited tree depth: comparable performance was obtained for constrained-depth models, for example 5-fold CV R^2 = 0.64 with max_depth = 10.

Most importantly, the central conclusion of the explainable analysis does not depend on a particular random-forest model overfitted to the original 66-run dataset. The expanded 215-sample analysis preserves the same qualitative structure identified from the original dataset: the E_U landscape is described largely by additive single-parameter

contributions with a residual T–O_2 interaction. The additive-only model reproduced the expanded 215-sample random-forest predictions only partially (R^2 = 0.72), whereas including a single (T-F_{O_2}) interaction term markedly improved the approximation (R^2 = 0.85). To quantify interaction strength, we also computed the Friedman H^2 interaction index from PDPs. The dominant interaction was obtained for the T-F_{O_2} pair (H^2 = 0.294), while other pairwise interactions were substantially smaller (≤ 0.063). We have described these points in the revised manuscript (page 13, line 27). This result, together with the successful re-optimization achieved using the human-executable strategy derived from the 66-run dataset, as described in our response to Reviewer #2, Comment 2 [(page 13, line 19) in this response letter], demonstrates that the random-forest analysis captures an experimentally actionable structure of the response surface rather than an artifact of overfitting.

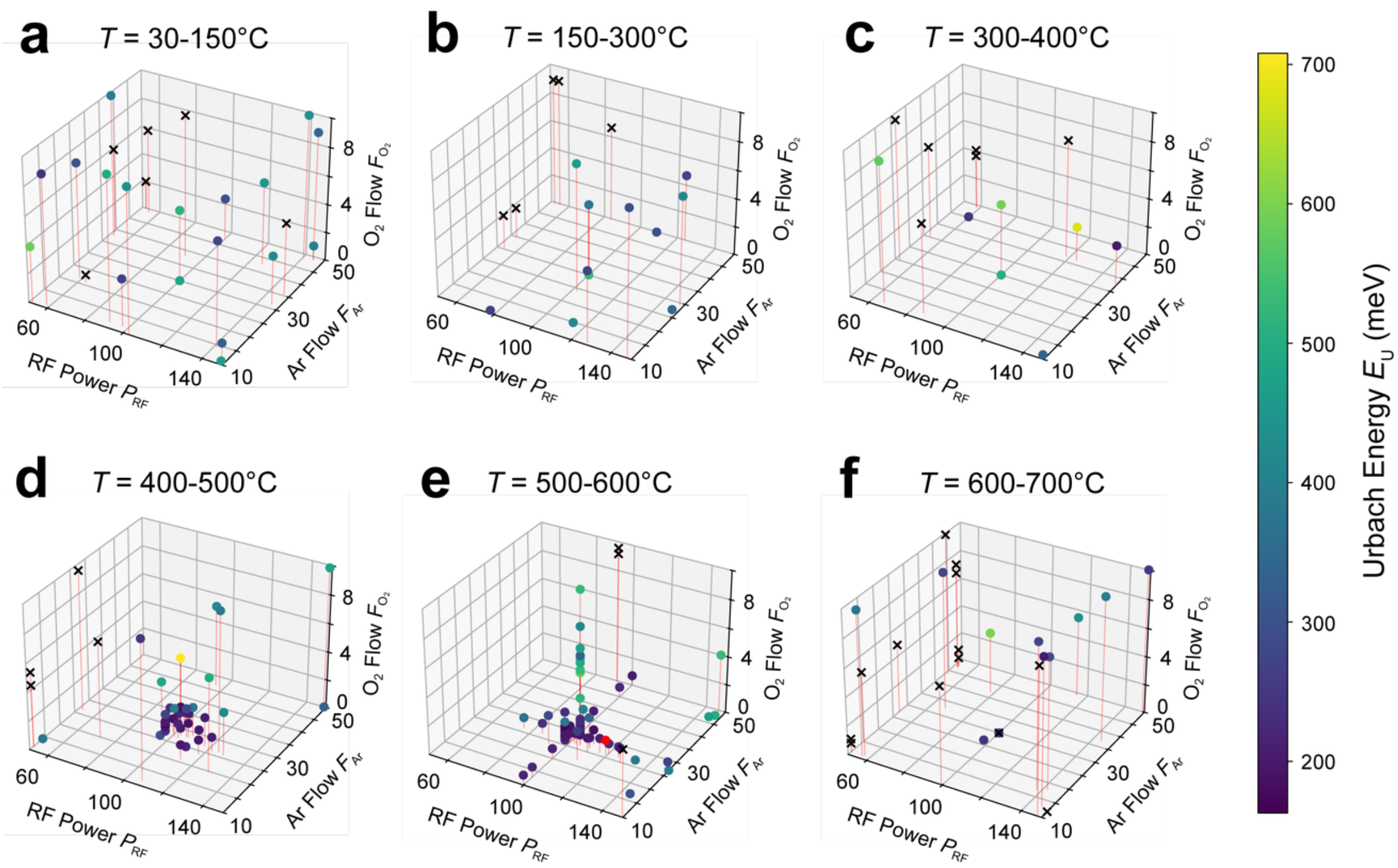

Fig. R12. Experimental E_U distribution in the expanded 215-sample dataset. The experimental E_U in the three-dimensional parameter space P_{RF}-F_{Ar}-F_{O_2} for different T windows of **a** 30-150°C, **b** 150-300°C, **c** 300-400°C, **d** 400-500°C, **e** 500-600°C, and **f** 600-700°C. Marker color represents E_U (color bar, meV), visualizing the distribution of the BO-acquired, additional sampling, and rule-guided re-optimization data. In **e**, the red circle represents the lowest E_U (163 meV) obtained in the rule-guided re-optimization. Black crosses indicate runs for which the E_U could not be extracted (NaN).

Comment 7:

Minor note:

The plots in Fig 6a-c are difficult to interpret in 3D. I suggest replacing them with 2D plots where PDP of E_U is represented by a perceptually uniform color scale.

Response:

Following the reviewer's suggestion, we have replaced these 3D surface plots with 2D heatmap representations, in which the PDP of E_U is represented using the perceptually uniform viridis color scale (Fig. R13). We also overlaid contour lines to further facilitate quantitative reading of the response surface. This revised presentation of Fig. 6 makes the response trends and interaction structure easier to interpret.

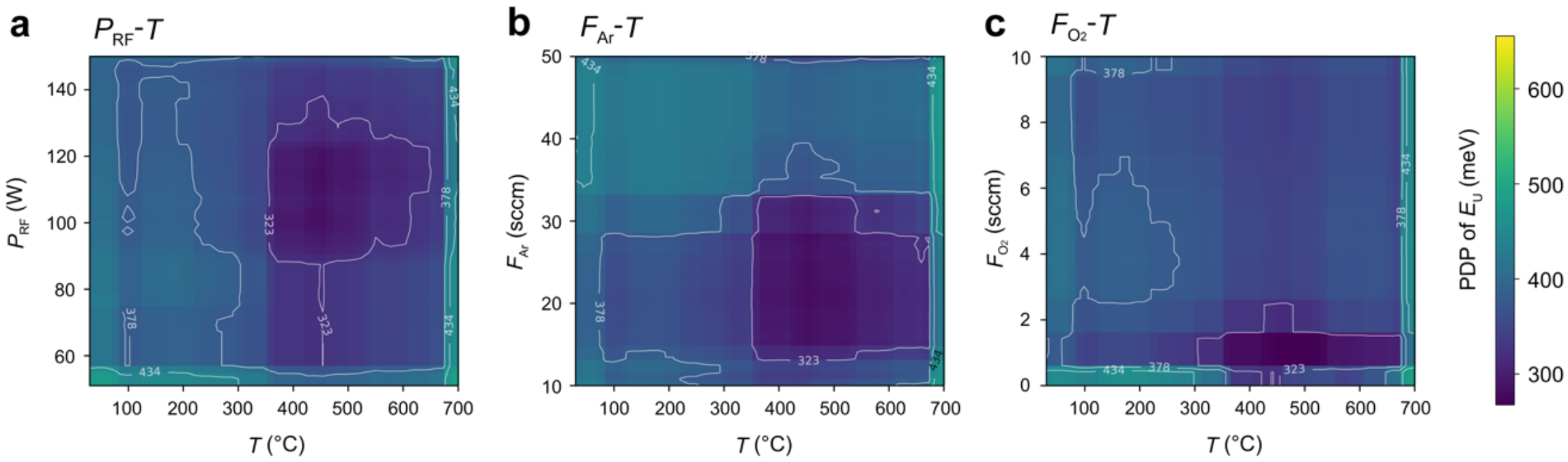

Fig. R13. Visualizing and quantifying pairwise interactions in the E_U landscape based on the original BO-acquired 66-run dataset. a-c 2D PDPs of E_U as a function of T and each other parameter: **a** P_{RF}, **b** F_{Ar}, and **c** F_{O_2}.

Comment 8:

(Remarks on code availability)

The data and code for the explainable ML portion of the paper is clear and easy to follow. However, only the optical analysis code and data from the SDL portion of the paper is provided. I strongly encourage the authors to share at least their Bayesian optimization code. Also sharing the code for running their SDL would make it much easier to repeat/reproduce such work in the future, and continue to help the community grow.

Response:

In response to this comment, we have additionally provided the source code used for the closed-loop BO, including surrogate-model training, acquisition-function optimization, and next-condition proposal. In the original submission, we provided the code for optical spectral analysis, including E_U extraction. Together, these codes enable reproduction of the modeling and optimization workflow from the measured optical spectra to the BO-suggested next growth conditions.

The low-level control software for the automated sputtering system and optical-measurement system, including vendor-specific equipment-control modules and APIs, cannot be made publicly available because it depends on proprietary instrument-control software and hardware-specific interfaces. However, we have clarified the instrument model, automated growth sequence, pre-sputtering step, and detailed experimental procedures in the revised Methods. These additions, together with the newly provided BO code and the optical-analysis code, should facilitate reproduction and extension of the workflow by the community.

Response letter

We would like to express our gratitude for considering our manuscript (#NCOMMS-26-010832A-Z) for publication in Nature Communications. We are also thankful to the reviewers for the careful reading of our manuscript and the valuable comments that helped us improve its quality. We have revised the manuscript in accordance with the reviewers' comments; the revised parts are indicated in red letters in the revised manuscript. The following are our point-by-point responses to the reviewer's comments.

Response to the comments of the reviewer #1

Comment 1:

The authors demonstrated an interpretable self-driving laboratory framework that transforms autonomous optimization data into human-executable growth rules. I have read the updated manuscript, and I still have the following major concerns for the paper:

1. Is it still not clear how substrate temperature T, RF power PRF, and gas flows and FAr/FO_2 were decided by the machine learning model.

Response:

We thank the reviewer for pointing out that the parameter-selection decision-making process was not sufficiently clear. Because the comment could refer either to the closed-loop BO decision-making process or to the subsequent human-executed re-optimization guided by the extracted rule, we have clarified both procedures as described below.

First, the BO procedure used to select sputter-growth parameters in the self-driving sputtering system is summarized in Supplementary Algorithm 1. The optimization objective was to minimize the Urbach energy E_U. The four-dimensional input vector was defined as $\boldsymbol{x} = (T,\ P_{RF},\ F_{Ar},\ F_{O_2})$, where T is the substrate temperature, P_{RF} is the RF power, and F_{Ar} and F_{O_2} are the Ar and O_2 flow rates, respectively. The first seven runs were used as seed experiments by sweeping T while fixing P_{RF}, F_{Ar}, and F_{O_2} at a pragmatic baseline condition. After these seed experiments, all subsequent conditions were selected as four-dimensional recipes by the BO loop. This procedure clarifies that, from run 8 onward, the BO loop selected T, P_{RF}, F_{Ar}, and F_{O_2} simultaneously as a four-

dimensional recipe. The random-forest surrogate used for partial-dependence and interaction analyses was not part of this self-driving decision-making loop; it was trained after the BO campaign to interpret the accumulated dataset and extract human-executable growth rules. We have described these points in the revised manuscript (page 4, line 18) and the revised Supplementary Information (page 3, line 1).

Supplementary Algorithm 1. Bayesian-optimization procedure used in the self-driving sputtering system.

Input: initial dataset $D_n = \{(\boldsymbol{x}_i, E_{\mathrm{U,i}})\}_{i=1}^{n=N_{\mathrm{init}}}$, total experimental budget N

Output: best parameter $\boldsymbol{x}_{i^*}$ such that $i^* = \arg\min_{1 \le i \le N} E_{\mathrm{U},i}$

```
n ← N_init
while n < N do
    Convert data D_n into D̃_n = {(x_i, ỹ_i)}_{i=1}^n by applying worst-value imputation by Eq. (7)
    fit Gaussian process using D̃
    calculate the expected improvement EI(x̃) over the normalized 4D parameter space x̃
    n ← n + 1
    find next parameter x̃_n = arg max_x̃ EI(x̃)
    rescale to original parameter x_n from x̃_n
    send x_n to the sputtering-control workflow
    grow the film under x_n
    measure the optical transmittance spectrum
    extract the Urbach energy E_U,n from the measured spectrum
    add the new result to raw dataset D_n ← D_{n−1} ∪ {(x_n, E_U,n)}
end while
```

Second, we have clarified how the extracted rule was implemented in the human-executed re-optimization. This re-optimization was performed manually by a human experimenter without using BO or another ML optimizer. Following the extracted growth rule derived from the random-forest analysis, the experimenter first performed sequential one-dimensional sweeps. Specifically, P_{RF}, F_{Ar}, and $F_{\mathrm{O_2}}$ were initially fixed at 100 W, 30 sccm, and 5 sccm, respectively, and T was swept over the allowed range. The experimenter then sequentially swept P_{RF}, F_{Ar}, and $F_{\mathrm{O_2}}$ while fixing the previously optimized parameters. The number of trials in each one-dimensional sweep was determined

empirically by the experimenter based on the observed E_U trends, rather than by an ML model. After this sequential one-dimensional procedure reached a low-E_U (183 meV) condition, (T, P_{RF}, F_{Ar}, F_{O_2}) = (512°C, 97 W, 26.5 sccm, 0.7 sccm), the experimenter performed a final restricted two-dimensional refinement in the T–F_{O_2} plane, with P_{RF} and F_{Ar} fixed, because the random-forest analysis identified T–F_{O_2} as the dominant residual interaction. This final step explored within restricted ranges of T = 462–612°C and F_{O_2} = 0–1.5 sccm. We have described this point in the revised manuscript [(page 12, line 14) and (page 12, line 17)]. This sequential 1D and 2D re-optimization could also be implemented using BO, but here it was intentionally performed manually to test whether the extracted rule could guide human-executable process optimization without relying on an autonomous decision layer.

Comment 2:

2. I agree with Reviewer 2 that the interpretability layer summarizes correlations in a BO-biased dataset rather than producing genuinely new, physics-grounded insights. It is not physically interpretable but mathematically interpretable.

Response:

We thank the reviewer for this important comment. We agree that the post hoc interpretability layer should not be presented as providing a complete microscopic physical mechanism. The random-forest partial-dependence and interaction analyses are data-driven and mathematically explainable tools for summarizing the experimentally sampled response surface. They do not, by themselves, establish mechanistic insight.

To avoid overstatement, we have revised the manuscript to clarify the scope of the extracted rules. Specifically, in the revised manuscript, we now describe the analysis as a post hoc explainable surrogate analysis that distills the closed-loop BO dataset into mathematically interpretable and human-executable process rules, rather than as a complete physical interpretation of the growth mechanism (page 17, line 12 in the revised manuscript).

At the same time, we would like to emphasize that, in the previous revision, we had taken this concern seriously because a related point was raised by Reviewer #2. In response to that earlier comment, we expanded the dataset from the original 66 BO-acquired

runs to 215 total growth runs, including additional space-filling experiments and human-executed re-optimization runs. The central response-surface structure, namely predominantly additive single-parameter contributions with a residual T-F_{O_2} interaction, was retained in this expanded dataset. We also experimentally tested the extracted rule as a human-executable optimization strategy. Sequential one-dimensional tuning followed by focused two-dimensional refinement in the T-F_{O_2} plane reproduced the low-E_U region and ultimately achieved E_U = 163 meV, lower than the original BO optimum. We note that Reviewer #2, who raised this related concern in the previous round, stated in the present round that they had no further questions. Thus, we have revised the manuscript to make clear that the contribution is not the discovery of a complete microscopic growth mechanism, but the conversion of autonomous BO data into a mathematically explainable and experimentally validated process rule. We believe this revised framing directly addresses the reviewer's concern while preserving the central advance of the work.

Comment 3:

3. The "transferability" demonstrated is indeed narrow. No other material system or deposition tools were involved.

Response:

As the reviewer pointed out, the transferability demonstrated in the present study should be defined and described carefully. We did not experimentally extend the extracted rule to a different material system or a different deposition tool. However, we did experimentally validate cross-substrate transferability within the same β-Ga_2O_3 sputtering system, by testing whether the low-E_U window identified from heteroepitaxial growth on C-plane Al_2O_3 also yields high-quality homoepitaxial β-Ga_2O_3 films.

In the previous revision, we strengthened this point by adding controlled homoepitaxial validation experiments. Homoepitaxial films grown under two independently identified low-E_U conditions both showed sharp β-Ga_2O_3 (020) reflections, whereas a film grown under an off-window, high-E_U condition showed a broad β-Ga_2O_3 (020) peak (Fig. R1). These results support a cross-substrate correspondence between the heteroepitaxial E_U landscape and homoepitaxial crystalline quality within the tested process space.

We had also clarified in the Discussion in the previous revision (page 17, line 3 in the revised manuscript) that the broader generality of the work lies not in the universality of the specific numerical recipe, but in the workflow for converting closed-loop optimization data into experimentally testable, human-executable process rules. This wording explicitly states the validation scope and avoids overgeneralization while preserving the demonstrated cross-substrate transferability.

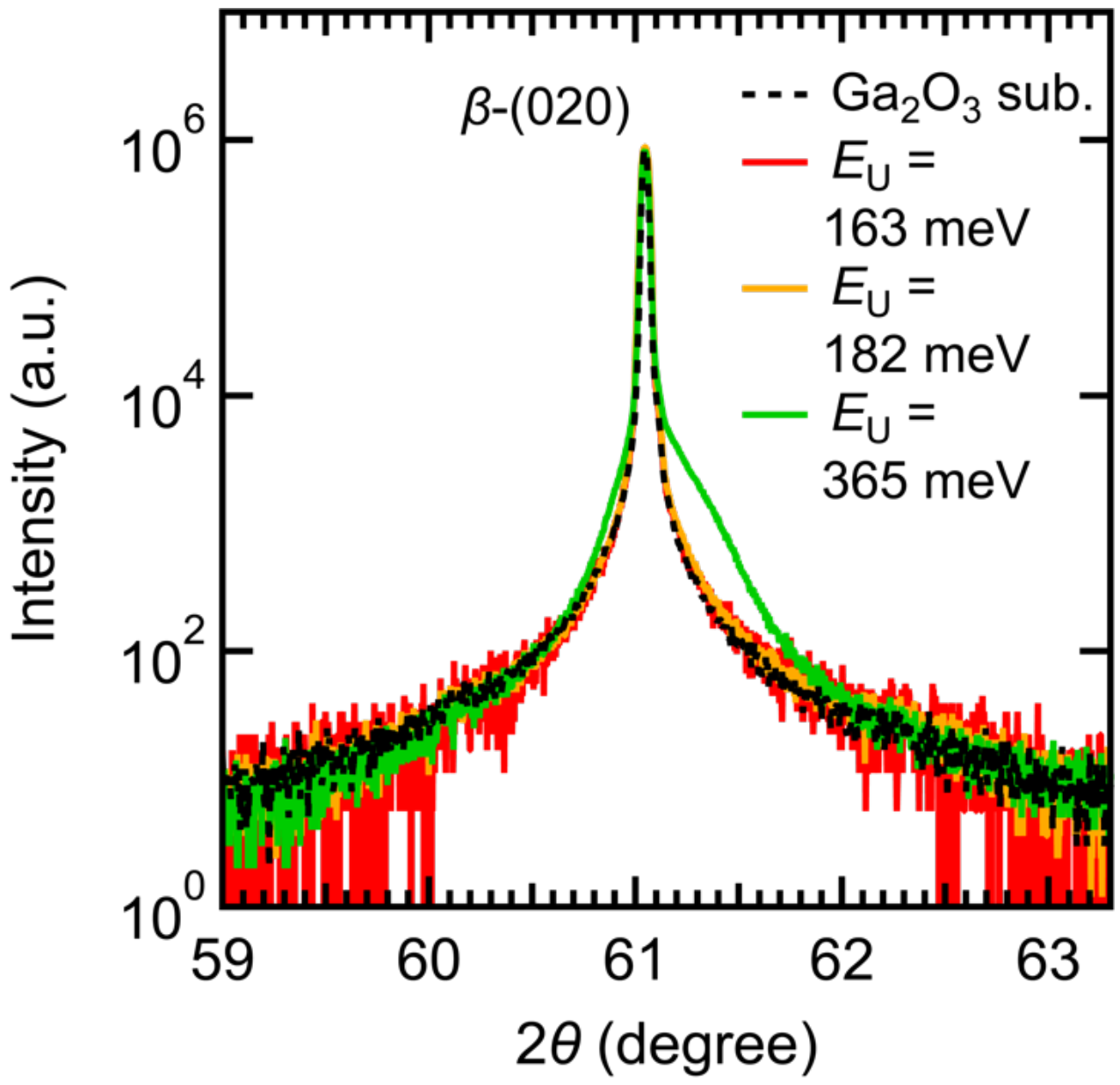

Fig. R1. XRD characterization of *β*-Ga_2O_3 homoepitaxial films. XRD *θ*-2*θ* scans of the *β*-(020) reflection for the *β*-Ga_2O_3 homoepitaxial films grown under low-E_U (163 and 182 meV) and high-E_U (365 meV) conditions, together with the substrate.

Comment 4:

4. The authors choose to do the first seven runs with fixed P_RF, F_Ar, and F_O2 while varying T, which already suggests that T is the most important parameter for growth.

Response:

We thank the reviewer for raising this point. The initial *T* sweep was chosen as a pragmatic commissioning step to confirm stable operation of the newly constructed sputtering system over a broad temperature range, rather than to impose the assumption that *T* would be the dominant parameter. We have described this point in the revised manuscript (page 5, line 5). After these seed experiments, all subsequent runs were selected by the BO

loop as four-dimensional recipes, $\boldsymbol{x} = (T,\ P_{\mathrm{RF}},\ F_{\mathrm{Ar}},\ F_{\mathrm{O_2}})$, so from run 8 onward the four parameters were optimized simultaneously by acquisition-function maximization. Furthermore, the expanded 215-run dataset added in the previous revision does not support a temperature-only interpretation. The response-surface analysis showed predominantly additive contributions from multiple growth parameters with a residual T-$F_{\mathrm{O_2}}$ interaction, and the expanded-dataset random-forest analysis identified $F_{\mathrm{O_2}}$ as a highly important parameter. These results indicate that the low-E_{U} window is governed by a multi-parameter balance rather than by T alone.

Comment 5:

Given these major concerns, I think the paper is not yet at the level expected by Nature Communications.

Response:

We thank the reviewer for the constructive comments. In response to the concerns raised above, we have revised the manuscript accordingly. We believe that these revisions substantially strengthen the manuscript and bring it to the high standard expected for publication in Nature Communications.

Comment 6:

I have run the codes myself, the results can fit the ones mentioned in the manuscript.

Response:

We sincerely thank the reviewer for running the codes and confirming that the results reproduce those reported in the manuscript. We appreciate this careful verification, which supports the reproducibility of the modeling and analysis workflow provided with the manuscript.

Response to the comments of the reviewer #3

Comment 1:

The authors have significantly improved the manuscript by adding new experiments, expanded analysis, and clearer methodological details. I think the revised work is technically sound, but a few claims should be tightened before acceptance.

Response:

We sincerely thank the reviewer for the positive assessment of the revised manuscript and for recognizing the technical soundness of our work. We have further revised the manuscript to tighten the remaining claims, as detailed in our responses below.

Comment 2:

First, the "long-standing challenge" should be framed more specifically. High-quality β-Ga2O3 epitaxy is already well established by other methods, so the novelty should be stated as achieving low-disorder, single-crystalline β-Ga2O3 epitaxy by scalable RF magnetron sputtering.

Response:

We thank the reviewer for this helpful suggestion. We agree that the challenge should be framed specifically in the context of sputtering epitaxy rather than β-Ga_2O_3 epitaxy in general. Accordingly, we revised the Abstract to state that the present work addresses "the long-standing challenge in sputtering epitaxy: realizing high-quality β-Ga_2O_3 heteroepitaxy and single-crystalline β-Ga_2O_3 homoepitaxy." This revision more precisely defines the novelty of the work as the achievement of high-quality β-Ga_2O_3 epitaxy by sputtering.

Comment 3:

Second, the manuscript should better separate automation throughput from algorithmic acceleration. The BO workflow reaches EU ≤ 182 meV at run 56, while the human-executed strategy reaches a similar level at about run 65. This suggests that the run-count advantage is modest, and the main practical gain may come from automation-enabled throughput.

Response:

As the reviewer pointed out, the run-count advantage of the BO workflow over the rule-guided human-executed strategy is modest. We have therefore revised the relevant text to separate the modest run-count difference from the practical working-time reduction enabled by automation throughput (page 13, line 13 in the revised manuscript). Specifically, we now clarify that the BO workflow reached $E_U \leq 182$ meV in 56 runs, whereas the human-executed strategy reached the same threshold in 65 runs, and that the approximately 2.7-fold reduction in working time mainly reflects the higher throughput of the automated self-driving workflow, rather than a large run-count advantage.

Comment 4:

Finally, the wording around "self-driving laboratories" and "black-box" optimization should be more precise. SDLs are not inherently black boxes; the black-box aspect mainly comes from the BO/surrogate decision layer. I suggest revising the abstract and related text to focus on closed-loop BO workflows rather than SDLs in general. especially the first sentence is difficult to understand and very confusing.

Response:

We thank the reviewer for pointing out the need for more precise terminology. We agree that self-driving laboratories themselves are not inherently black boxes, and that the relevant black-box aspect in the present work lies in the Bayesian optimization decision layer, where the process–property relationship is treated as an objective function for selecting subsequent experimental conditions. To clarify this point, we revised the first sentence of the Abstract as follows: "Self-driving laboratories are powerful tools for navigating high-dimensional process spaces, yet Bayesian-optimization decision layers optimize black-box objective functions without distilling transferable process understanding." This revision further aligns the Abstract with the framing used in the Introduction and Discussion, where our critique is directed at closed-loop Bayesian optimization workflows that identify optimized recipes without necessarily converting the acquired data into transferable, human-usable growth rules.

Comment 5:

After these relatively minor wording and framing revisions, I would support publication.

<u>Response:</u>

We thank the reviewer for the supportive comment. We have revised the wording and framing accordingly and hope that the revised manuscript is now suitable for publication.
