# Supplementary material for: Interpretable self-driving sputtering epitaxy reveals human-usable growth rules for β-Ga2O3 films
Source: Nat Commun. 2026 Aug 13;17:9714. doi: 10.1038/s41467-026-76533-0 (PMC13562525; doi:10.1038/s41467-026-76533-0)
Supplement: Supplementary file 5 [file 41467_2026_76533_MOESM3_ESM.zip › Supplementary_Data/License.docx]

**SOFTWARE LICENSE AGREEMENT FOR EVALUATION**

This SOFTWARE LICENSE AGREEMENT FOR EVALUATION (this "Agreement") is a legal contract between a person who uses or otherwise accesses or installs the Software (“User(s)”), and NTT, Inc.

READ THE TERMS AND CONDITIONS OF THIS AGREEMENT CAREFULLY BEFORE INSTALLING OR OTHERWISE ACCESSING OR USING NTT'S PROPRIETARY SOFTWARE ACCOMPANIED BY THIS AGREEMENT (the "SOFTWARE"). THE SOFTWARE IS COPYRIGHTED AND IT IS LICENSED TO USER UNDER THIS AGREEMENT, NOT SOLD TO USER. BY INSTALLING OR OTHERWISE ACCESSING OR USING THE SOFTWARE, USER ACKNOWLEDGES THAT USER HAS READ THIS AGREEMENT, THAT USER UNDERSTANDS IT, AND THAT USER ACCEPTS AND AGREES TO BE BOUND BY ITS TERMS. IF AT ANY TIME USER IS NOT WILLING TO BE BOUND BY THE TERMS OF THIS AGREEMENT, USER SHOULD　TERMINATE THE INSTALLATION PROCESS, IMMEDIATELY CEASE AND REFRAIN FROM ACCESSING OR USING THE SOFTWARE AND DELETE ANY COPIES USER MAY HAVE. THIS AGREEMENT REPRESENTS THE ENTIRE AGREEMENT BETWEEN USER AND NTT CONCERNING THE SOFTWARE.

**BACKGROUND**

A. NTT is the owner of all rights, including all patent rights, copyrights and trade secret rights, in and to the Software and related documentation except OSS listed in Exhibit A to this Agreement.
B. User wishes to obtain a royalty free license to use the Software to enable User to evaluate, and NTT wishes to grant such a license to User, pursuant and subject to the terms and conditions of this Agreement.
C. As a condition to NTT's provision of the Software to User, NTT has required User to execute this Agreement.

In consideration of these premises, and the mutual promises and conditions in this Agreement, the parties hereby agree as follows:

# 1. Grant of Evaluation License. NTT hereby grants to User, and User hereby accepts, under the terms and conditions of this Agreement, a royalty free, nontransferable and nonexclusive license to use the Software internally for the non-commercial purposes of testing, analyzing, and evaluating the methods or mechanisms as shown in the research paper submitted by NTT to a certain academy or technical contest, etc. ("academy"). User may make a reasonable number of backup copies of the Software solely for User's internal use pursuant to the license granted in this Section 1.

2.　Shipment and Installation. NTT will ship or deliver the Software by any method that NTT deems appropriate. User shall be solely responsible for proper installation of the Software.

# 3.　Term. This Agreement is effective whichever is earlier (i) upon User’s acceptance of the Agreement, or (ii) upon User’s installing, accessing, and using the Software, even if User has not expressly accepted this Agreement. Without prejudice to any other rights, NTT may terminate this Agreement without notice to User (i) if User breaches or fails to comply with any of the limitations or other requirements described herein, and (ii) if NTT receives a notice from the academy stating that the research paper would not be published, and in any such case User agrees that NTT may, in addition to any other remedies it may have at law or in equity, remotely disable the Software. User may terminate this Agreement at any time by User’s decision to terminate the Agreement to NTT and ceasing use of the Software. Upon any termination or expiration of this Agreement for any reason, User agrees to uninstall the Software and either return to NTT the Software and all copies thereof, or to destroy all such materials and provide written verification of such destruction to NTT.

# 4. Proprietary Rights

(a)	The Software and Data are the valuable, confidential, and proprietary property of NTT, and NTT shall retain exclusive title to this property both during the term and after the termination of this Agreement. Without limitation, User acknowledges that all patent rights, copyrights and trade secret rights in the Software except OSS shall remain the exclusive property of NTT at all times. User shall use not less than reasonable care in safeguarding the confidentiality of the Software and the Data.

(b)	NTT shall not be subject to the obligation of licensing the copyright, patent rights, etc. of author when user hope commercial / noncommercial use of the published / provided software, etc.

## (c) USER SHALL NOT, IN WHOLE OR IN PART, AT ANY TIME DURING THE TERM OF OR AFTER THE TERMINATION OF THIS AGREEMENT: (i) SELL, ASSIGN, LEASE, DISTRIBUTE, OR OTHERWISE TRANSFER THE SOFTWARE TO ANY THIRD PARTY; (ii) EXCEPT AS OTHERWISE PROVIDED HEREIN, COPY OR REPRODUCE THE SOFTWARE IN ANY MANNER; (iii) DISCLOSE THE SOFTWARE TO ANY THIRD PARTY, EXCEPT TO USER'S EMPLOYEES WHO REQUIRE ACCESS TO THE SOFTWARE FOR THE PURPOSES OF THIS AGREEMENT; (iv) MODIFY, DISASSEMBLE, DECOMPILE, REVERSE ENGINEER OR TRANSLATE THE SOFTWARE; OR (v) ALLOW ANY PERSON OR ENTITY TO COMMIT ANY OF THE ACTIONS DESCRIBED IN (i) THROUGH (iv) ABOVE.

## (d) User shall take appropriate action, by instruction, agreement, or otherwise, with respect to its employees permitted under this Agreement to have access to the Software to ensure that all of User's obligations under this Section 4 shall be satisfied.

# 5.　 Indemnity. User shall defend, indemnify and hold harmless NTT, its agents and employees, from any loss, damage, or liability arising in connection with User's improper or unauthorized use of the Software. NTT SHALL HAVE THE SOLE RIGHT TO CONDUCT DEFEND ANY ACTTION RELATING TO THE SOFTWARE.

# 6. Disclaimer. THE SOFTWARE IS LICENSED TO USER "AS IS," WITHOUT ANY TRAINING, MAINTENANCE, OR SERVICE OBLIGATIONS WHATSOEVER ON THE PART OF NTT. NTT MAKES NO EXPRESS OR IMPLIED WARRANTIES OF ANY TYPE WHATSOEVER, INCLUDING WITHOUT LIMITATION THE IMPLIED WARRANTIES OF MERCHANTABILITY, OF FITNESS FOR A PARTICULAR PURPOSE AND OF NON-INFRINGEMENT ON COPYRIGHT OR ANY OTHER RIGHT OF THIRD PARTIES. USER ASSUMES ALL RISKS ASSOCIATED WITH ITS USE OF THE SOFTWARE, INCLUDING WITHOUT LIMITATION RISKS RELATING TO QUALITY, PERFORMANCE, DATA LOSS, AND UTILITY IN A PRODUCTION ENVIRONMENT.

# 7. Limitation of Liability. IN NO EVENT SHALL NTT BE LIABLE TO USER OR TO ANY THIRD PARTY FOR ANY INDIRECT, SPECIAL, INCIDENTAL, OR CONSEQUENTIAL DAMAGES, INCLUDING BUT NOT LIMITED TO DAMAGES FOR PERSONAL INJURY, PROPERTY DAMAGE, LOST PROFITS, OR OTHER ECONOMIC LOSS, ARISING IN CONNECTION WITH USER'S USE OF OR INABILITY TO USE THE SOFTWARE, IN CONNECTION WITH NTT'S PROVISION OF OR FAILURE TO PROVIDE SERVICES PERTAINING TO THE SOFTWARE, OR AS A RESULT OF ANY DEFECT IN THE SOFTWARE. THIS DISCLAIMER OF LIABILITY SHALL APPLY REGARD­LESS OF THE FORM OF ACTION THAT MAY BE BROUGHT AGAINST NTT, WHETHER IN CONTRACT OR TORT, INCLUDING WITHOUT LIMITATION ANY ACTION FOR NEGLIGENCE. USER'S SOLE REMEDY IN THE EVENT OF ANY BREACH OF THIS AGREEMENT BY NTT SHALL BE TERMINATION PURSUANT TO SECTION 3.

# 8. No Assignment or Sublicense. Neither this Agreement nor any right or license under this Agreement, nor the Software, may be sublicensed, assigned, or otherwise transferred by User without NTT's prior written consent.

# 9. General

# (a) If any provision, or part of a provision, of this Agreement is or becomes illegal, unenforceable, or invalidated, by operation of law or otherwise, that provision or part shall to that extent be deemed omitted, and the remainder of this Agreement shall remain in full force and effect.

## (b) This Agreement is the complete and exclusive statement of the agreement between the parties with respect to the subject matter hereof, and supersedes all written and oral contracts, proposals, and other communications between the parties relating to that subject matter.

## (c) Subject to Section 8, this Agreement shall be binding on, and shall inure to the benefit of, the respective successors and assigns of NTT and User.

## (d) If either party to this Agreement initiates a legal action or proceeding to enforce or interpret any part of this Agreement, the prevailing party in such action shall be entitled to recover, as an element of the costs of such action and not as damages, its attorneys' fees and other costs associated with such action or proceeding.

## (e) This Agreement shall be governed by and interpreted under construed in accordance with the laws of Japan, without reference to conflicts of law principles. All disputes arising out of or in connection with this Agreement shall be finally settled by arbitration in Tokyo in accordance with the Commercial Arbitration Rules of the Japan Commercial Arbitration Association. The arbitration shall be conducted by three (3) arbitrators and in Japanese. The award rendered by the arbitrators shall be final and binding upon the parties. Judgment upon the award may be entered in any court having jurisdiction thereof.

(f)　　	NTT shall not be liable to the User or to any third party for any delay or failure to perform NTT’s obligation set forth under this Agreement due to any cause beyond NTT’s reasonable control.

**EXHIBIT A**

All the files in the GitHub repository.
